# Immunogenicity and safety of an inactivated whole-virus COVID-19 vaccine (VLA2001) compared with the adenoviral vector vaccine ChAdOx1-S in adults in the UK (COV-COMPARE): interim analysis of a randomised, controlled, phase 3, immunobridging trial

**DOI:** 10.1016/S1473-3099(22)00502-3

**Published:** 2022-12

**Authors:** Rajeka Lazarus, Benedicte Querton, Irena Corbic Ramljak, Shailesh Dewasthaly, Juan Carlos Jaramillo, Katrin Dubischar, Michael Krammer, Petronela Weisova, Romana Hochreiter, Susanne Eder-Lingelbach, Christian Taucher, Adam Finn, Valneva phase 3 trial group, Claire Bethune, Marta Boffito, Marcin Bula, Fiona M Burns, Rebecca Clark, Dileep Dasyam, Simon Drysdale, Saul Faust, Effrossyni Gkrania-Klotsas, Christopher Green, Hana Hassanin, Paul Heath, Amardeep Heer, Toby Helliwell, Anil Hormis, Philip Kalra, Rajeka Lazarus, Ed Moran, John Ndikum, Iain Page, David Price, Nick Probert, Mahadev Ramjee, Tommy Rampling, Harpal S Randeva, Stephen Ryder, John Steer, Emma Thompson, David Torku

**Affiliations:** a University Hospitals Bristol and Weston NHS Foundation Trust, Bristol, UK; b Valneva Austria, Vienna, Austria; c School of Population Health Sciences and School of Cellular and Molecular Medicine, University of Bristol, Bristol, UK

## Abstract

**Background:**

The Valneva COVID-19 vaccine (VLA2001; Valneva Austria, Vienna, Austria) is an inactivated whole-virus, adjuvanted SARS-CoV-2 vaccine. We aimed to assess the safety and immunogenicity of primary vaccination with VLA2001 versus the ChAdOx1-S (Oxford-AstraZeneca) adenoviral-vectored vaccine.

**Methods:**

In this immunobridging phase 3 trial (COV-COMPARE), participants aged 18 years and older who were medically stable (as determined by an investigator) were enrolled at 26 sites in the UK. In the double-blind, randomised, controlled arm of the trial, participants aged 30 years and older were randomly assigned (2:1) to receive two doses of VLA2001 (0·5 mL; with 33 antigen units [AU] per dose) or ChAdOx1-S (0·5 mL; with 2·5 × 10^8^ infectious units per dose) on days 1 and 29. In another arm, participants aged 18–29 years received two doses of VLA2001 (same dose) open label on days 1 and 29. The primary immunogenicity outcome was the immune response of a two-dose schedule of VLA2001 on day 43, in adults aged 30 years and older, versus two doses of ChAdOx1-S via superiority of geometric mean titres (GMTs) of neutralising antibodies (GMT ratio of >1 at a two-sided significance level of 5%) and non-inferiority of the seroconversion rate (non-inferiority margin of –10% for the lower limit of the 95% CI for the difference between groups). The primary safety outcome was the frequency and severity of any adverse events in all participants up to day 43. Safety was assessed in all participants who received at least one dose of vaccine. GMTs were assessed in a subset of participants aged 30 years and older who were seronegative at baseline, had at least one evaluable antibody titre measurement after vaccination, and had no confirmed COVID-19 during the study (immunogenicity population); and seroconversion was assessed in the per-protocol population, which comprised the immunogenicity population but excluding any participants with major protocol violations. For each timepoint, only participants with available data were included in the analysis. This study is registered with ClinicalTrials.gov, NCT04864561, and is ongoing.

**Findings:**

Between April 28 and June 3, 2021, 4181 individuals were screened and 4017 enrolled, of whom 2975 (74%) were aged 30 years or older and randomly assigned to receive VLA2001 (n=1978) or ChAdOx1-S (n=997), and 1042 (26%) were aged 18–29 years (all received open-label VLA2001). 4012 participants received at least one dose of vaccine (1040 in the open-label VLA2001 group, 1977 in the randomised VLA2001 group, and 995 in the ChAdOx1-S group). The immunogenicity population comprised 492 participants in the randomised VLA2001 group and 498 in the ChAdOx1-S group; three participants in the VLA2001 group were excluded from the per-protocol population. VLA2001 induced higher neutralising GMTs than did ChAdOx1-S (803·5 [95% CI 748·5–862·6] *vs* 576·6 [543·6–611·7]; GMT ratio 1·39 [95% CI 1·25–1·56]; p<0·0001), and non-inferior seroconversion rates (444 [97·4%] of 456 participants *vs* 444 [98·9%] of 449; difference –1·5% [95% CI –3·3 to 0·2]. Any adverse event was reported in 963 (92·6%) participants in the open-label VLA2001 group, 1755 (88·8%) in the randomised VLA2001 group, and 976 (98·1%) in the ChAdOx1-S group. Most adverse events reported were mild or moderate in severity.

**Interpretation:**

VLA2001 has a favourable tolerability profile and met superiority criteria for neutralising antibodies and non-inferiority criterion for seroconversion rates compared with ChAdOx1-S. The data presented here formed the basis of successful marketing approval for use of VLA2001 in primary vaccination in the EU, the UK, Bahrain, and United Arab Emirates.

**Funding:**

UK Department of Health and Social Care and Valneva Austria.

**Research in context**

**Evidence before this study**

We searched PubMed for research articles, using no filters or language restrictions, published from database inception up to June 15, 2022, using the terms “pivotal” AND “comparative” AND “immunogenicity” AND “COVID-19” AND “vaccine”. We identified nine reports, all of which referred to mRNA vaccines in different specific populations (eg, adolescents, children aged <5 years, people aged >50 years, allogeneic haematopoietic cell transplant recipients, and patients with cirrhosis). Repeating the search using the terms (“COVID-19” OR “SARS-CoV-2”) AND “immuno-bridging” AND “vaccine” identified three reports. One study compared ChAdOx1-S with the whole virus inactivated vaccine Covaxin (manufactured by the Serum Institute of India), and highlighted the non-inferior immune response of Covaxin compared with ChAdOx1-S. One study reported on MVC-COV1901 (Medigen Vaccine Biologics, Taiwan), an adjuvanted protein-subunit vaccine, which has been granted emergency use authorisation in Taiwan on the basis of a non-inferior immunobridging analysis against ChAdOx1-S (recombinant). The third paper was an immunobridging study on non-inferiority of the immune response in adolescents compared with the adult population for the BBIBP-CorV vaccine (Sinopharm, China). The safety and optimal dose of VLA2001 was previously assessed in a phase 1/2 study, which found that VLA2001 was well tolerated in all tested dose groups. The highest dose group (35 antigen units per dose) showed significantly stronger immunogenicity than lower-dose groups, with similar tolerability and safety, and so was selected for phase 3 clinical development. A schedule of two doses given 28 days apart (vaccination given on days 1 and 29) was chosen on the basis of the schedule for the licensed comparator, ChAdOx1-S.

**Added value of this study**

This study represents the first pivotal immunobridging comparison of the inactivated COVID-19 vaccine VLA2001 to a previously authorised vaccine, ChAdOx1-S, in Europe, with full marketing authorisation granted by the European Medicines Agency, conditional Marketing Authorisation granted by the Medicines and Healthcare Products Regulatory Agency in the UK, and emergency use authorisation granted by the United Arab Emirates and Bahrain on the basis of these results. VLA2001's safety and tolerability profile were favourable, and VLA2001 induced higher levels of neutralising antibodies (nAb) against SARS-CoV-2 than ChAdOx1-S, a vaccine that has proven efficacy against COVID-19, at 43 days (14 days after the second dose). Furthermore, VLA2001 is the only vaccine licensed in the EU that induces a T-cell response targeting other antigenic epitopes in addition to the spike protein.

**Implications of all the available evidence**

Production of VLA2001 uses a traditional manufacturing technology that has been used for other licensed vaccines that are on the market. This type of vaccine reduces logistical complexity and eases vaccine supply in remote global locations because it can be refrigerated for several months. In light of emerging SARS-CoV-2 variants of concern, a vaccine that induces a broad immune response targeting additional antigenic epitopes that are less prone to mutations is desirable. The positive results presented here support regulatory recognition, and worldwide use of VLA2001. Future research is required to examine the antibody persistence over time and the timing, safety, and immunogenicity of booster vaccinations with VLA2001.

## Introduction

According to the WHO COVID-19 dashboard, as of Aug 9, 2022, the COVID-19 pandemic has caused more than 6·4 million deaths worldwide. Inactivated viral vaccines have been successfully used in immunisation programmes for decades and are seen as safe and reliable. For example, alum-adjuvanted inactivated vaccines against COVID-19 effectively produce a strong immune response, significantly reduce symptomatic COVID-19 risk, and have been widely used in global efforts to control the pandemic.^1^, ^2^, ^3^, ^4^, ^5^, ^6^ Because these vaccines contain the whole virus, inactivated vaccines can induce a broader immune response than vaccines that only feature one particular viral component. With this goal in mind, Valneva Austria (Vienna, Austria) developed VLA2001, a whole-virus SARS-CoV-2 vaccine produced in Vero cells and inactivated by β-propiolactone, preserving the native surface structure of the virus spike (S) protein. The viral strain used was derived from a Chinese tourist from Hubei province who was diagnosed in a hospital in Rome, Italy.^7^ It shares more than 99% sequence homology with the ancestral Wuhan reference sequence.^8^ VLA2001 is adjuvanted with cytosine phosphoguanine (CpG) 1018, an adjuvant also contained in the approved Heplisav B vaccine against hepatitis B,^9^ and with aluminium hydroxide, an ancillary vaccine component used since the 1930s.^10^ VLA2001 has been found to have a good safety profile in a phase 1/2 trial,^11^ and has been investigated as a heterologous third-dose booster.^12^

Because placebo-controlled COVID-19 efficacy trials are no longer deemed ethically acceptable, given that a number of effective and safe vaccines are available, appropriately designed immunobridging studies have become an accepted approach to regulatory approval.^13^ Thus, the objective of this phase 3 clinical study was to show superiority of VLA2001 in terms of neutralising antibodies (nAbs) over another SARS-CoV-2 vaccine for which efficacy has previously been found. Because no other whole-virus inactivated vaccine was available for clinical studies (ie, licensed in the EU or UK and accessible for conduct of studies), the comparator chosen in consultation with the study cofunder—the UK Department of Health and Social Care—was the COVID-19 vaccine ChAdOx1-S (recombinant; Oxford-AstraZeneca), a replication-deficient chimpanzee adenoviral vector containing the SARS-CoV-2 structural surface glycoprotein antigen (S protein; nCoV-19) gene.^14^ ChAdOx1-S showed high immunogenicity and efficacy against COVID-19 and a reduction of symptomatic cases compared with placebo of 74% against the Wuhan and alpha variant (B.1.1.7) strains.^15^ As of Aug 7, 2022, ChAdOx1-S is approved for administration in 180 countries, more countries than any other vaccine.^16^ Here, we present the primary interim analysis, with immunogenicity results obtained up to day 43 after baseline (2 weeks after the second dose) and safety data up to the defined timepoints (7 days for solicited adverse events, day 43 for the primary safety endpoint, and data cutoff (Oct 14, 2021) for secondary safety endpoints).

## Methods

### Study design and participants

In this randomised, controlled, parallel-arm, multicentre, immunobridging, phase 3 trial (COV-COMPARE), key exclusion criteria were acute illness (including SARS-CoV-2 infection) within 48 h before vaccination, pregnancy, known allergies to any vaccine component, and any immunosuppressive condition or receipt of immunosuppressive therapy. Full eligibility criteria are listed in the protocol (appendix 1).

The study had an independent data safety monitoring board tasked with monitoring the accruing safety data. The study was approved by the Medicines and Healthcare Products Regulatory Agency (MHRA) UK and the Leeds West research ethics committee (IRAS project identification number 294164). This clinical trial is closed to new participants but ongoing for follow-up until day 365 (12 months after first vaccination) or until 6 months after booster vaccination. The study is being conducted in accordance with the Good Clinical Practice guidelines of the International Council for Harmonization and the principles of the Declaration of Helsinki. Written informed consent was provided by each participant before enrolment. A randomised, placebo-controlled part of the study in adolescents (aged ≥12 to <18 years) and a booster phase, in which all participants except those who had already received a licensed COVID-19 vaccine outside of the study have been offered a booster dose of VLA2001, were introduced to the study in later protocol versions. Data collection in these groups is still ongoing. Therefore, the data presented here are restricted to the primary immunisation of the adult participants.

### Randomisation and masking

Eligible participants aged 30 years and older were randomly assigned in an overall 2:1 ratio to receive two intramuscular doses of either VLA2001 or ChAdOx1-S 28 days apart (ie, on days 1 and 29). The overall randomisation ratio of 2:1 was obtained as follows: participants were assessed for their COVID-19 serostatus at screening by rapid antibody test (AbC-19 Rapid Test, Abingdon Health, York, UK). Seronegative participants were randomly assigned in a 1:1 ratio to receive either VLA2001 or ChAdOx1-S, until a total of 1200 participants had been randomly assigned (these individuals comprised the immunogenicity subset, from whom the immunogenicity population for analysis of the primary endpoint was derived). Remaining seronegative participants, and all seropositive participants aged 30 years and older, were randomly assigned in a 7:2 ratio to VLA2001 or ChAdOx1-S. We used block randomisation, with block sizes of multiples of two (for the initial 1:1 ratio) and nine (for the subsequent 7:2 ratio), and the randomisation number scheme consisted of five digits. Vaccine group allocation was concealed from the study personnel involved in the outcome assessments. Each site had a masked and an unmasked study team, and only the masked team was involved in outcome assessment. Designated personnel involved in obtaining randomisation codes and preparing the vaccines were not masked to treatment allocation. All other laboratory and medical personnel and participants were masked to treatment assignment. The randomisation scheme was generated using SAS (version 9.4 or higher).

Participants aged 18–29 years were allocated to an open-label treatment group and received two doses of VLA2001 on days 1 and 29. Participants in this age range were not assigned to the comparator vaccine ChAdOx1-S because it was not recommended in this age group in the UK at the time of study enrolment.

### Procedures

Vaccines were administered at the study sites on the basis of the randomisation codes. The vaccination schedule consisted of two 0·5 mL vaccine doses given 28 days apart for each study participant, administered by intramuscular injection in the deltoid region of the non-dominant arm. VLA2001 is an inactivated, SARSCoV-2 vaccine adjuvanted with CpG and Alum (33 antigen units [AU] per dose; lot number CL00003). This dose was selected on the basis of the results of a phase 1/2 study (VLA2001-201),^17^ and the administration schedule was adjusted from 21 days to 28 days to match the ChAdOx1-S schedule. The recommended registered dose for ChAdOx1-S, as approved in the UK, was used (0·5 mL; with 2·5 × 10^8^ infectious units per dose; lot number PV46677).

At baseline (day 0), participants had a physical examination and medical history was obtained. Participants received their first vaccination on day 1, their second vaccination on day 29, and returned for an on-site visit on day 43. Symptom-driven physical examinations were done at all visits. Blood samples (20 mL) were collected from all participants on day 1 (before first vaccination), day 29 (before second vaccination), and day 43, and serum separation was done at the clinical sites according to a standardised protocol defined in the laboratory manual. Serum samples were shipped to central laboratories for wild-type microneutralisation assay (WT-MNA) and ELISA analyses. At five sites, all participants in the immunogenicity subset also provided 50 mL whole blood for isolation of peripheral blood mononuclear cells (PBMCs) on days 1, 29, and 43. These sites were selected on the basis of proximity to the central laboratory where PBMC isolation and analysis were done.

SARS-CoV-2-specific neutralising antibody responses in serum samples were measured using a live WT-MNA against the Victoria strain by the UK Health Security Agency (Porton Down, UK).^18^ The virus was isolated from a 58-year-old man from Wuhan, China, after his arrival in Melbourne in January, 2020.^19^ Neutralising titres are expressed as the serum dilution at which 50% of virus is neutralised compared with a negative control (ND_50_). Values below the limit of quantification of the WT-MNA (ND_50_= 62; ie, titre of <62) were replaced by the lower limit of quantification divided by 2, which equates to 31. Reported values were replaced with the highest sample dilution tested (SDUL) for results given as ND_50_ greater than the SDUL.

Additionally, serum samples were analysed for SARS-CoV-2 anti-S IgG (S protein ELISA) at Nexelis (Laval, QC Canada). Values below the quantitation limit of the ELISA (50·3 ELISA laboratory units [ELU] per mL) were replaced by 25 ELU per mL. Values above the quantitation limit (15 798 ELU per mL) were replaced by 15 798 ELU per mL. Assay variability was determined to be less than 25% of the coefficient of variation.

For the assessment of cell-mediated responses, PBMCs were stimulated in vitro with S, nucleocapsid (N), and membrane (M) proteins using T-Spot Discovery SARS-CoV-2 (Oxford Immunotec, Oxford, UK). T-cell responses were classified as reactive if 6 or more spot-forming units (SFU) per 2 × 10^5^ PBMCs were present. SFUs were determined as single values for each stimulation and were normalised to the respective unstimulated control by subtracting the number of SFUs in the medium-only control. Assay variability was determined to be less than 25% of the coefficient of variation.

Participants recorded solicited local (injection-site pain, itching, tenderness, redness, and swelling or induration) and systemic (fever and body temperature, fatigue, headache, nausea and vomiting, and muscle pain) adverse events in electronic diaries for 7 days after each vaccination, starting on the day of vaccination. Additionally, serious adverse events and adverse events of special interest (including complications associated with COVID-19, because of the theoretical risk for disease enhancement, and immune-mediated disorders due to the addition of the CpG 1018 adjuvant) were collected and reported by the investigator according to applicable regulations during the entire study period. Investigators followed up all adverse events and, for unsolicited adverse events, assessed causal associations with study vaccines on the basis of their clinical judgement. Severity grading of unsolicited adverse events was carried out by investigators using their clinical expertise and judgment and the criteria described in the protocol (appendix 1 p 68).

Participants who developed COVID-19-related symptoms after randomisation were advised to request PCR testing for SARS-CoV-2 at the clinical site or, if available, in the community, and to contact the study site immediately if they had high fever (≥38·0°C) or shortness of breath, or if milder symptoms (eg, sore throat, chills, cough, body aches, new loss of taste or smell, runny nose, nausea, or diarrhoea) persisted for at least 2 consecutive days.

### Outcomes

The primary immunogenicity endpoint was the immune response measured after completion of a two-dose immunisation schedule with VLA2001, as determined in adults aged 30 years and older by the geometric mean titre (GMT) ratio and seroconversion of SARS-CoV-2-specific nAbs on day 43. Secondary endpoints were GMTs and seroconversion of SARS-CoV-2-specific nAbs on days 8 (only in participants aged >55 years) and 29; GMTs and seroconversion rates of anti-S IgG on days 8 (only in participants aged >55 years), 29, and 43; and T-cell responses (Th1/Th2 polarisation) from PBMCs. For both nAbs and anti-S IgG, seroconversion was defined as an at least four-times increase in antibody titres over baseline (ie, an at least four times greater titre than their respective prevaccination titre) as described previously.^3^ This definition was confirmed in formal scientific advice by MHRA and the European Medicines Agency (EMA).^20^ Day 29 data are not available, and so not reported here; these data will be reported elsewhere.

The primary safety endpoint was the frequency and severity of any adverse events up to day 43 after baseline in all participants. Secondary safety outcomes were the frequency and severity of solicited adverse events within 7 days after each vaccine dose, frequency and severity of any unsolicited adverse events and any unsolicited vaccine-related adverse events (including medically attended adverse events) after completion of two-dose immunisation scheduled with VLA2001, and the frequency and severity of any adverse event, with attention to serious adverse events, adverse events of special interest, throughout the duration of the study. Severe unsolicited adverse events were reported up to Aug 11, 2021, which includes an observation period of more than 43 days for some participants.

An exploratory outcome was the number of PCR-confirmed symptomatic and asymptomatic cases of COVID-19 per treatment group starting from 14 days after the second vaccine dose up to data cutoff (Oct 14, 2021). Analyses of additional endpoints defined in the protocol concerning the adolescent study population and the booster vaccination are ongoing and are therefore not included in this interim report.

### Statistical analysis

We did a primary interim analysis after all participants were vaccinated and had completed the day 43 follow-up visit, the data for which are presented here (safety data until data cutoff [Oct 14, 2021]). A second interim analysis is planned when all participants have completed the day 208 follow-up visit, and a final analysis is planned once the last participant has completed the study.

We selected the sample size for this study to establish a comprehensive safety database and to characterise the safety profile of VLA2001. We determined that inclusion of 3000 participants vaccinated with VLA2001 would allow for the detection of unusual events (with an incidence rate of ≥1 per 1000 participants) with a probability of 94%. We estimated proportions of participants in each group with adverse events along with exact Clopper-Pearson 95% CIs. We planned to assess immunogenicity in 1200 participants aged 30 years and older (ie, 600 per treatment group), which would allow for a statistical power of 90% to detect superiority in terms of the day 43 GMT ratio of VLA2001 to ChAdOx1-S, with an expected ratio of 1·3, an SD of 0·6 (on a log_10_ scale) of the WT-MNA test, an expected drop-out rate of 10%, and a two-sided significance level of 5%.

All baseline, safety, and tolerability analyses were in the safety population, which included all participants who entered the study and received at least one study vaccination; they were to be analysed as treated. The data cut-off for inclusion of safety data was Oct 14, 2021.

We used the immunogenicity population for determination of antibody GMTs. This population comprised participants aged 30 years and older who were randomly assigned to the immunogenicity subset (ie, negative for SARS-CoV-2 infection by rapid antibody test) who were seronegative at baseline as determined by WT-MNA, and had at least one evaluable post-baseline antibody titre measurement after vaccination. Participants with confirmed COVID-19 during the study were excluded from the immunogenicity population.

The per-protocol population comprised the immunogenicity population but excluded participants with major protocol violations that might affect the immune response (eg, receiving fewer than two vaccinations) and was used for analysis of seroconversion. The PBMC population comprised approximately 200 randomly selected participants (100 per treatment group) from the immunogenicity population, and was used to analyse T-cell responses.

Post-hoc, we included participants aged 18–29 years in the open-label VLA2001 group in immunogenicity analyses (GMT and seroconversion of neutralising and IgG antibodies). For this population, immunogenicity was analysed in a random selection of participants who were baseline seronegative by WT-MNA and all baseline seropositive participants from that age group. Participants were randomly selected using an SAS program, using the *RANUNI* function.

For the primary immunogenicity analysis, we tested superiority using the day 43 GMT ratio of VLA2001 versus ChAdOx1-S in the immunogenicity population using a two-sided *t* test applied to neutralisation titres after log_10_ transformation. We postulated that VLA2001 would be superior to ChAdOx1-S if the day 43 GMT ratio was significantly different between the groups and greater than 1. For the primary endpoint assessment, non-inferiority of seroconversion was tested on the percentage difference between day 43 seroconversion rates (VLA2001 *vs* ChAdOx1-S) in the per-protocol population. For the difference, we calculated the two-sided exact 95% Clopper-Pearson CI, and non-inferiority of VLA2001 was postulated if the lower limit of the 95% CI was greater than –10%. Non-inferiority of seroconversion rates was also assessed using a –5% margin. The primary endpoint was met for VLA2001 if the requirements of both tests–superiority of GMT and non-inferiority of seroconversion rate–were met. All immunogenicity analyses were calculated for both immunogenicity and per-protocol populations as part of prespecified secondary endpoints.

In sensitivity analyses, we estimated GMTs along with corresponding 95% CIs by applying ANOVA for log_10_-transformed nAb titres, including the factors of treatment group and study site. We back-transformed (anti-log_10_) estimates (least square means, least square mean differences, and the corresponding 95% CIs) obtained from ANOVA to obtain the estimates of GMT and corresponding 95% CI. To test how a set of independent variables affects GMTs, we present the number and proportion of participants with nAb seroconversion at day 43 along with exact 95% Clopper-Pearson CIs for both study groups. As a post-hoc analysis, we stratified immunogenicity data by baseline serostatus (as determined by WT-MNA and by age group (18–29 years, 30–55 years, >55 years) in the VLA2001 groups (data are not presented for the ChAdOx1-S group; the immune response to ChAdOx1-S in seropositive or previously infected individuals has been described in detail elsewhere^21^).

For the exploratory outcome of occurrence of asymptomatic and symptomatic cases of COVID-19 per treatment group, we calculated the hazard ratio (HRs) of PCR-confirmed SARS-CoV-2 infection 14 days after the second vaccination between the randomised treatment groups. In a post-hoc analysis, we compared the immunogenicity of VLA2001 in those aged 18–29 years versus those aged 30 years and older. For this analysis, we randomly selected participants aged 18–29 years in the open-label VLA2001 group who were seronegative at screening (results for seropositive participants are described separately) and compared them with the immunogenicity population aged 30 years and older. Non-inferiority was postulated if the lower limit of the CI for the GMT ratio was above 0·67. Superiority of GMT was postulated in case of a significant GMT ratio of more than 1.

We did all analyses using SAS (version 9.4 or higher). This study is registered with ClinicalTrials.gov, NCT04864561.

### Role of the funding source

Valneva Austria designed the protocol, supervised study conduct, and analysed the study results, which have also undergone detailed review by MHRA and EMA. Valneva Austria, in collaboration with the other authors, had a role in data collection, data analysis, data interpretation, writing of the report, and the decision to submit the manuscript. The UK Department of Health and Social Care had no role in study design, data collection, data analysis, data interpretation, or writing of the report.

## Results

**Figure 1:**
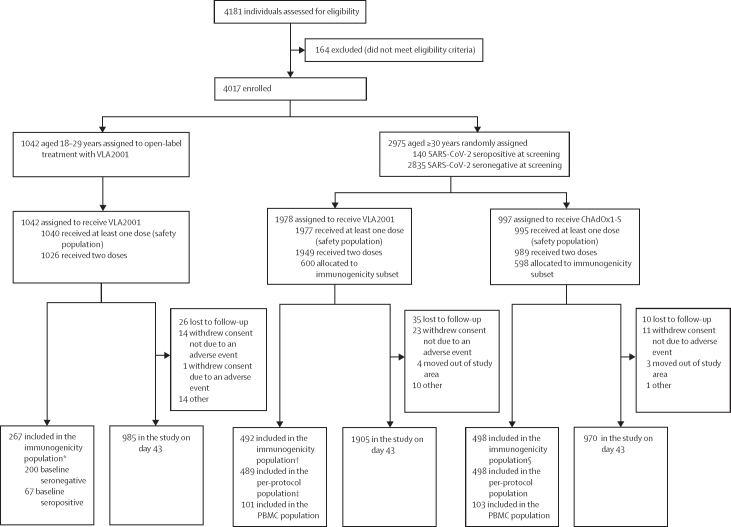
Study profile PBMC=peripheral blood mononuclear cells. *Of 302 randomly selected, 35 participants were excluded from the immunogenicity analysis due not receiving vaccine (n=1), missing sample on day 43 or no baseline tire (n=25), or SARS-CoV-2 infection (n=9). †108 (18%) of 600 participants in the immunogenicity subset were excluded from the immunogenicity population for the following reasons: positive by WT**-**MNA at baseline (n=84; 14%); SARS-CoV-2 infection (n=13; 2%); and missing sample on day 43 or no baseline tire (n=11; 2%). ‡Three participants in the immunogenicity population were excluded from the per-protocol population for the reason of not having received the second vaccination. §100 (16·7%) of 598 participants in the immunogenicity subset were excluded from the immunogenicity population for the following reasons: positive by WT**-**MNA at screening (n=79; 13%); SARS-CoV-2 infection (n=11; 2%); and missing sample on day 43 or no baseline tire (n=10; 2%).

Between April 28 and June 3, 2021, 4181 individuals were screened and 4017 were enrolled. 1042 (25·9%) participants were aged 18–29 years and assigned to the open-label VLA2001 group and 2975 were aged 30 years and older and assigned to the randomised VLA2001 (1978 [49·2%]) and ChAdOx1-S (997 [24·8%]) groups (figure 1). Overall, five participants were enrolled but not vaccinated; as such, 4012 (99·9%) participants were included in the safety population. 1198 participants aged 30 years and older were randomly assigned into the immunogenicity subset (600 in the VLA2001 group and 598 in the ChAdOx1-S group), of whom 208 (17·4%) were later excluded from the immunogenicity population (figure 1). Hence, 990 (24·6%) of 4017 participants aged 30 years and older were included in the immunogenicity population. Three participants in the immunogenicity population were excluded from the per-protocol population, leaving 987 (24·6%) participants aged 30 years and older in the per-protocol population (figure 1). The PBMC subset of the immunogenicity population included 204 (5·1%) participants (figure 1). In the 18–29 years age group, 200 randomly selected participants who were seronegative at baseline by WT-MNA and all 67 who were seropositive at baseline by WT-MNA**,** were included in the post-hoc non-inferiority comparison with the VLA2001 age 30 years and older immunogenicity population group (figure 1).

Overall, among participants who received at least one dose of vaccine, participant demographics were similar between the VLA2001 age 30 years and older group and the ChAdOx1-S group (table 1). The most common major protocol deviation was procedures (ie, visits) conducted outside of the defined visit window (data not shown). As of data cutoff (Oct 14, 2021), participants had been followed-up for a mean of 151·4 days (SD 19·3).

**Table 1:** Baseline demographic and clinical characteristics, safety population

	**Open-label VLA2001 group (age 18–29 years; N=1040)**	**Randomised VLA2001 group (age ≥30 years; N=1977)**	**ChAdOx1-S group (age ≥30 years; N=995)**
**Age at time of informed consent, years**			
Mean (SD)	24·4 (3·23)	35·4 (5·02)	35·6 (4·81)
Median	25·0	34·0	35·0
Minimum–maximum	18–29	30–68	30–71
IQR	22·0–27·0	32·0–38·0	32·0–38·0
18–29	1040 (100%)	0	0
30–55	0	1958 (99·0%)	990 (99·5%)
>55	0	19 (1·0%)	5 (0·5%)
**Sex**			
Male	555 (53·4%)	1135 (57·4%)	567 (57·0%)
Female	483 (46·4%)	839 (42·4%)	427 (42·9%)
Diverse	2 (0·2%)	3 (0·2%)	1 (0·1%)
**BMI at screening, kg/m^2^**			
n	1037	1975	993
Mean (SD)	25·44 (5·05)	27·25 (5·37)	27·43 (5·54)
Median	24·40	26·20	26·50
Minimum–maximum	16–49	16–80	17–58
IQR	21·8–28·0	23·5–30·0	23·4–30·3
Ethnicity*			
White	955 (91·8%)	1844 (93·3%)	927 (93·2%)
Mixed	39 (3·8%)	38 (1·9%)	23 (2·3%)
Asian	23 (2·2%)	54 (2·7%)	22 (2·2%)
**COVID-19 test result at screening**†			
Seropositive	52 (5·0%)	108 (5·5%)	32 (3·2%)
Seronegative	988 (95·0%)	1869 (94·5%)	963 (96·8%)

Data are n (%), unless otherwise stated.

* Most frequently reported (≥2% incidence) ethnicities are included.

† By rapid antibody test.

The incidences of any adverse event up to day 43 were 963 (92·6%) of 1040 participants in the open-label VLA2001 group, 1755 (88·8%) of 1977 in the randomised VLA2001 group, and 976 (98·1%) of 995 in the ChAdOx1-S group (table 2). Since only a small number of adverse events were defined as severe, we assume that most adverse events reported were mild or moderate in severity (table 2), with proportions of severe solicited adverse events being slightly higher in the ChAdOx1-S group than in the open-label and randomised VLA2001 groups and proportions of severe unsolicited adverse events (reported up to Aug 11, 2021) being similar across groups (table 2). The incidences of serious adverse events, medically attended adverse events, and adverse events of special interest up to day 43 were similar between all treatment groups (table 2).

**Table 2:** Overall summary of adverse events up to day 43 in the safety population

		**Open-label VLA2001 group (age 18–29 years; N=1040)**	**Randomised VLA2001 group (age ≥30 years; N=1977)**	**ChAdOx1-S group (age ≥30 years; N=995)**
Any adverse event		963 (92·6%)	1755 (88·8%)	976 (98·1%)
Solicited adverse events				
	At least one systemic reaction after any vaccination	800 (76·9%)	1387 (70·2%)	906 (91·1%)
	At least one injection-site reaction after any vaccination	841 (80·9%)	1448 (73·2%)	906 (91·1%)
	Any severe systemic reaction*	5 (0·5%)	19 (1·0%)	46 (4·6%)
	Any severe injection-site reaction*	0	1 (0·1%)	8 (0·8%)
	Any serious adverse event	0	0	0
	Any medically attended adverse event	3 (0·3%)	3 (0·2%)	9 (0·9%)
	Any adverse event ongoing beyond diary period	13 (1·3%)	21 (1·1%)	19 (1·9%)
Unsolicited adverse events				
	Any	300 (28·8%)	566 (28·6%)	349 (35·1%)
	Any severe adverse event†	15 (1·4%)	30 (1·5%)	14 (1·4%)
Any treatment-related adverse event‡		955 (91·8%)	1719 (86·9%)	975 (98·0%)
Any serious adverse event		2 (0·2%)	6 (0·3%)	3 (0·3%)
Any medically attended adverse event		78 (7·5%)	138 (7·0%)	72 (7·2%)
Any adverse event of special interest		2 (0·2%)	1 (0·1%)	2 (0·2%)

Data are n (%). Data shown here are up to day 43 for the primary dafety endpoint, and up to Aug 11, 2021 for severe unsolicited adverse events.

* Solicited systemic reactions are counted as severe if they prevent daily activity, or are potentially life-threatening and require admission to hospital. Fever (≥30·0°C) is graded as severe. Injection site reactions are counted as severe if they prevent daily activity or require use of narcotic pain reliever (pain), cause significant discomfort at rest (tenderness), or if redness, induration or swelling exceeds diameters specified in the protocol or cause necrosis, or if an emergency room visit or admission to hospital is required. Participants who had more than one episode of the same reaction are summarised only once under maximum severity grade. No solicited adverse event was graded as potentially life-threatening.

† Unsolicited adverse events are counted as severe if they make the participant incapable of work or usual activity and require medical intervention or are potentially life-threatening. Participants who had more than one episode of the same reaction are summarised only once under maximum severity grade.

‡ All solicited adverse events are counted as related; unsolicited adverse events are counted as related if causality was determined to be probable or possible.

Notably, significantly fewer participants in the randomised VLA2001 (age ≥30 years) group than in the ChAdOx1-S group reported solicited adverse events up to 7 days after the first vaccination, both with regards to local injection-site reactions (1180 [59·7%] of 1977 *vs* 877 [88·1%] of 995; p<0·0001; appendix 2 p 1) and systemic reactions (1187 [60·0%] *vs* 876 [88·0%]; p<0·0001; appendix 2 p 4). Injection-site tenderness and fatigue were the most frequently reported solicited reactions after the first and second vaccine dose in all groups (appendix 2 pp 1–2, 4–5). No significant differences were observed between groups for any of the injection-site reactions after the second vaccine dose (appendix 2 p 2). Significantly more participants reported solicited systemic reactions in the ChAdOx1-S group than in the randomised VLA2001 (age ≥30 years) group after the second vaccine dose (501 [50·7%] of 989 *vs* 908 [46·6%] of 1949]; p=0·037; appendix 2 p 5). Fewer participants who received VLA2001 (age ≥30 years) reported solicited injection-site or systemic reactions within 7 days of either vaccination than did those who received ChAdOx1-S (appendix 2 pp 3, 6). No serious solicited adverse events were reported in any treatment group.

Unsolicited adverse events were reported by 300 (28·8%) of 1040 participants in the open-label VLA2001 group, 566 (28·6%) of 1977 in the randomised VLA2001 group, and 349 (35·1%) of 995 in the ChAdOx1-S group, with a significant difference in the incidence between the randomised VLA2001 and ChAdOx1-S groups (p=0·0003; appendix 2 pp 7–8). By day 43, the only unsolicited adverse events occurring in more than 2% of all participants overall were oropharyngeal pain (135 [3·4%] of 4012 participants) and headache (112 [2·8%]). The incidence of these unsolicited adverse events were similar across all groups. During the entire safety follow-up period, only 31 (0·8%) of 4012 participants reported a serious unsolicited adverse event (appendix 2 pp 9–10). The incidences of unsolicited serious adverse events were similar between all treatment groups; 0·7% in both VLA2001 groups (seven of 1040 in the open-label VLA2001 group and 14 of 1977 in randomised VLA2001 group) and ten (1·0%) of 995 in the ChAdOx1-S group (appendix 2 pp 12–13). None of the serious unsolicited adverse events were considered to be related to the vaccination.

**Figure 2:**
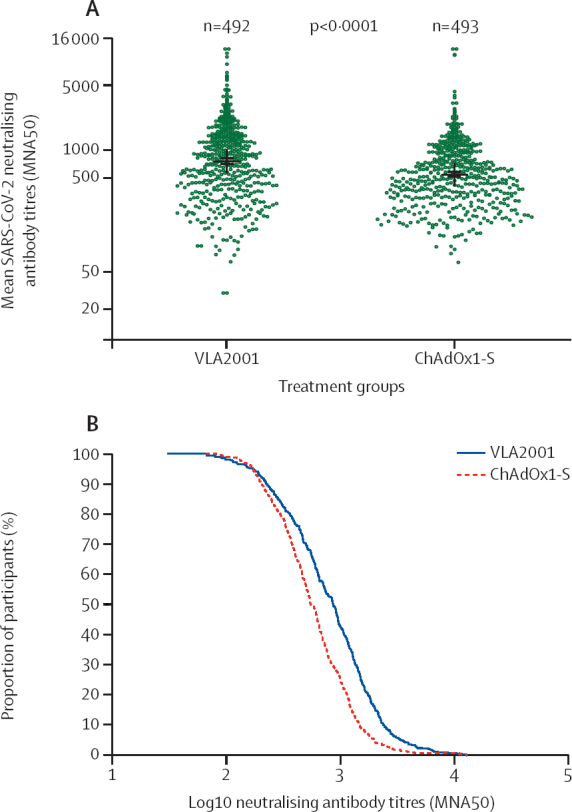
SARS-CoV-2 neutralising antibodies on day 43 (A) and reverse cumulative distribution function of SARS-CoV-2 neutralising antibodies on day 43 (B) in participants aged 30 years and older (immunogenicity population) In panel A, the whiskers show the mean neutralising antibody titres and 95% CI, and the datapoints show actual distribution of titres. In panel B, data are shown for 492 participants in the VLA2001 group and 493 in the ChAdOx1-S group who had available data on day 43. ND50=50% virus neutralisation titre measured in a microneutralisation assay.

On day 43, in the immunogenicity population, GMTs in the randomised VLA2001 group were significantly higher than those in the ChAdOx1-S group (803·5 [95% CI 748·48–862·59] *vs* 576·6 [543·59–611·66]; p<0·0001; table 3). Superiority of VLA2001 was confirmed with a GMT ratio of 1·39 (95% CI 1·25–1·56). A scatterplot of SARS-CoV-2 nAbs (ND_50_) over time by study group is presented in figure 2A (immunogenicity population); the reverse cumulative distribution function for SARS-CoV-2 nAb titres (ND_50_) for day 43 by treatment group for the immunogenicity population is presented in figure 2B, showing the distribution of neutralising antibody titres across participants and superiority of the neutralising antibody response in the randomised VLA2001 group compared with the ChAdOx1-S group. Results for the per-protocol population were similar to those of the immunogenicity population (data not shown).

**Table 3:** SARS-CoV-2 neutralising antibody titres (ND_50_) on day 43, in the immunogenicity population

	**Open-label VLA2001 group (age 18–29 years; N=200)**	**Randomised VLA2001 group (age ≥30 years; N=492)**	**ChAdOx1-S group (age ≥30 years; N=498)**	GMT ratios (95% CI; p value)*
n	200	492	493	..
GMT (95% CI)	1043·4 (926·6–1174·9)	803·5 (748·5–862·6)	576·6 (543·6–611·7)	..
Median	1118·5	867·0	553·0	..
Minimum–maximum	87–11036	31–12800	66–12800	..
IQR	657·0–1841·0	439·0–1520·0	340·0–1001·0	..
VLA2001 (age ≥30 years) *vs* ChAdOx1-S (age ≥30 years)	..	..	..	1·39 (1·25–1·56; p<0·0001)
VLA2001 (age 18–29 years) *vs* VLA2001 (age ≥30 years; post-hoc analysis)	..	..	..	1·3 (1·1–1·5; p=0·0008)

Data on day 43 were only available for 493 of 498 participants in the ChAdOx1-S group who were in the immunogenicity population. GMT=geometric mean titre. ND_50_=50% neutralising dilution.

* p value and 95% CI were calculated using a two-sided *t* test applied to log_10_-transformed data.

In the per-protocol population, the proportion of participants with seroconversion on day 43 was 97·4% (444 of 456 participants) among VLA2001 recipients aged 30 years and older and 98·9% (444 of 449) among ChAdOx1-S recipients (table 4). No significant difference was noted (p=0·091); non-inferiority was confirmed with a lower bound of the 95% CI for the difference between the randomised groups of –3·3%. Results for the immunogenicity population were similar to those of the per-protocol population (data not shown).

**Table 4:** SARS-CoV-2 neutralising antibody seroconversion rate on day 43, in the per-protocol population

	**Open-label VLA2001 group (age 18–29 years; N=200)**	**Randomised VLA2001 group (age ≥30 years; N=489)**	**ChAdOx1-S group (age ≥30 years; N=498)**	**Difference in seroconversion rate (95% CI; p value)**
Assessable population	198	456	449	..
Number who seroconverted	195 (98·5%)	444 (97·4%)	444 (98·9%)	..
95% CI*	95·6 to 99·7	95·4 to 98·6	97·4 to 99·6	..
VLA2001 (age ≥30 years) *vs* ChAdOx1-S (age ≥30 years)	..	..	..	−1·5% (−3·3 to −0·2; 0·0911)

Data on day 43 were not available for all members of the per-protocol population. Seroconversion was defined as four-times or greater increase in SARS-CoV-2-specific neutralising antibody titres between day 1 and post-vaccination sample collection timepoints.

* Exact 95% Clopper-Person CI for proportion.

In a post-hoc analysis by age group, in three VLA2001 recipients in the immunogenicity population who were older than 55 years, the nAb GMT on day 43 was 611·4 (158·91–2352·01); there were no participants in this age group in the ChAdOx1-S group. No data are shown for day 8, because all samples had titers below the limit of detection. For the age 30–55 years group, the nAb GMT on day 43 was 804·9 (95% CI 749·5–864·3) in the VLA2001 group. No participants in the ChAdOx1-S group were above 55 years of age.

Among individuals aged 30 years in the immunogenicity subset who received VLA2001 and were seropositive at baseline, on day 1 the GMT of nAbs was 269·2 (95% CI 226·4–320·0), with a range of 62·0 to 6738·0. By day 43, the nAb GMT had increased to 1478·6 (1245·6–1755·1). Additionally, nAb titres on day 43 in VLA2001 recipients, regardless of age (18–29 years or ≥30 years), who were WT-MNA positive at baseline were higher than in participants who were seronegative at baseline (table 3; appendix 2 p 11).

In a post-hoc analysis, we compared nAb GMTs of a randomly selected population of VLA2001 recipients aged 18–29 years with those aged 30 years and older. At day 43, GMTs of nAbs were significantly higher in the VLA2001 age 18–29 years group (1043·4 [95% CI 926·6–1174·9]) than in the 30 years and older age group (803·5 [748·5–862·6]). Thus, non-inferiority of the VLA2001 aged 18–29 years group was confirmed, with the lower limit of the CI above the predefined non-inferiority margin value of 0·67. Moreover, superiority of this age group was shown with a GMT ratio of 1·3 (95% CI 1·1–1·5; p=0·0008).

Binding-antibody GMTs (95% CI) against SARS-CoV-2 S protein on day 43 in the immunogenicity population were 2361·7 (95% CI 2171·08–2569·11) in the randomised VLA2001 group and 2126·4 (1992·42–2269·45) in the ChAdOx1-S group. In the open-label VLA2001 group, the GMT of anti-S IgG on day 43 was 3121·5 (2699·3–3609·7; appendix 2 p 15). In the per-protocol population, 447 (98·0%) of 456 participants in the randomised VLA2001 group and 445 (98·9%) of 450 in the ChAdOx1-S group had seroconverted at day 43. The difference between the treatment groups of 0·9% (95% CI –2·5 to 0·7) was not significant (p=0·29; appendix 2 p 13). Notably, the S-protein binding antibody response to VLA2001 after the first priming dose (at day 29) was lower than after ChAdOx1-S (appendix 2 pp 12–13). No data are shown for day 8 because all samples had titers below the limit of detection.

Cellular immune responses were analysed in the PBMC population. The numbers of reactive IFN-γ-producing T cells detected after stimulation with peptide panels of S protein N-terminus and full sequence N and M proteins are shown in the appendix 2 (p 14). As expected, in recipients of ChAdOx1-S, a cellular immune response was seen only after stimulation with S protein peptides. On day 43, 55 (74·3%) of 74 participants with available samples for this timepoint in the VLA2001 group and 64 (86·5%) of 74 participants in the ChAdOx1-S group had a T-cell response against peptide pools spanning the full-length S protein detected. In the VLA2001 group, 34 (45·9%) of 74 participants had responses to the N protein and 15 (20·3%) had responses to the M protein, compared with none of 74 to the N protein and one (1·4%) of 74 to the M protein in the ChAdOx1-S group (appendix 2 p 14).

The exploratory analysis of COVID-19 cases from 14 days after the second vaccine dose up to data cutoff (Oct 14, 2021) revealed similar numbers of cases across the treatment groups: 69 (7·3%) of 951 participants in the open-label VLA2001 group, 99 (4·9%) of 1794 in the randomised VLA2001 group, and 42 (4·5%) of 941 in the ChAdOx1-S group with available data. No severe SARS-CoV-2 infections were reported. Furthermore, the hazard ratio for the difference in PCR-confirmed COVID-19 cases starting 14 days after the second vaccination between the randomised groups of participants aged 30 years and older was not significant (hazard ratio 0·98, 95% CI 0·68–1·42; appendix 2 p 18).

## Discussion

We found that VLA2001 is a well tolerated vaccine with significantly lower reactogenicity than ChAdOx1-S, both with regards to solicited injection-site and systemic reactions. VLA2001 was highly immunogenic, with a SARS-CoV-2 nAb GMT ratio on day 43 of 1·39 versus ChAdOx1-S, showing immunological superiority over the comparator. Although the observed drop-out rate was higher than anticipated, the standard deviation was lower than expected (observed SD was 0·5), which resulted in a statistical power of 96·2% for this analysis. In terms of seroconversion rates, VLA2001 was non-inferior to ChAdOx1-S. Notably, the superiority of VLA2001 after two doses was not reflected in antibody responses after the first dose, emphasising the importance of receipt of both priming doses. In a small number of participants who had detectable nAbs before immunisation, presumably due to undocumented infection, VLA2001 appeared to be at least as immunogenic as in participants who were seronegative. This finding is similar to data described in the literature, in which administration of ChAdOx1-S to people who had previously had a SARS-CoV-2 infection provided a strong antibody response.^21^

This study has some limitations. First, due to the high coverage of the UK national vaccination campaign in older age groups at the time of this study, the number of participants older than 55 years who were willing and able to enrol was too small to allow meaningful conclusions to be drawn in this age group. Second, young adults aged 18–29 years could only be enrolled in an open-label group due to regulatory guidance not to vaccinate participants in this age group with ChAdOx1-S. Nevertheless, the safety and immunogenicity data for the aged 18–29 years group were similar to the older age group and were equal or superior to that of adults aged 30 years and older who received VLA2001. Further limitations are that only healthy individuals and individuals with stable conditions were selected for participation, and all study sites were in the UK, where successful engagement of people from minority ethnic groups in clinical research unfortunately remains low, resulting in a largely White and thus ethnically unrepresentative study sample. Finally, at present, the available immunogenicity data only cover a short period up to day 43 after the first vaccination and do not cover immunogenicity against variants of concern.

Despite the absence of an established individual serological correlate of protection, nAb and S-protein-binding antibody GMTs are associated with vaccine efficacy and can thus act as predictors for immune protection. This has been shown for mRNA-based, vector-based, and inactivated SARS-CoV-2 vaccines.^22^, ^23^, ^24^ The most comprehensive published data on the association of nAb concentrations with vaccine efficacy are available for ChAdOx1-S.^14^ 80% efficacy against symptomatic infection with SARS-CoV-2 (majority alpha variant) after vaccination with ChAdOx1-S correlated with a normalised neutralisation titre (ND50) of 247,^25^ which corresponds to a WT-MNA GMT of 488 in the same live-virus neutralisation assay we used in this study. The clear correlation between nAb GMTs and efficacy led to the communication from European regulators that, when attempting an immunobridging approach, a superiority design instead of a non-inferiority design would be required. This approach aims to ensure that a new vaccine candidate performs better than a licensed vaccine, with no margin for non-inferiority with regards to antibody titres. This immunobridging study forms the core of full market authorisation of VLA2001 in the EU, conditional marketing authorisation in the UK and emergency use authorisation in Bahrain and the United Arab Emirates, and is expected to support emergency use authorisation of VLA2001 in other countries in need of additional COVID-19 vaccine options.

Most of the advanced and licensed COVID-19 vaccines are based on the SARS-CoV-2 S protein. However, the virus particle consists of three more structural proteins, the M, N, and envelope (E) proteins. In a natural infection, the immune system recognises all these proteins to varying degrees. Here, we found that VLA2001 as a whole-virus vaccine promotes broad T-cell responses, also including the N and M proteins. Analyses of serum samples from convalescent patients and T-cell responses have shown that there are long-lasting antibody and T-cell responses against the N protein after disease recovery.^26^ Other licensed vaccines in the EU and UK, which rely on the S protein only, do not elicit this type of response. Further studies of VLA2001 will aim to investigate whether this broader immune response will translate into longer-lasting immune responses than other available vaccines and into effectiveness of VLA2001 against more recent variants. Although our study was not designed or powered to show vaccine efficacy, SARS-CoV-2 infections were closely monitored and, if possible, sequenced (data not shown). The occurrence of COVID-19 cases, an exploratory endpoint, was similar between treatment groups in participants aged 30 years and older, and no cases of severe COVID-19 occurred during observation period up to data cutoff, when SARS-CoV-2 infections were caused predominantly by the delta virus variant in the UK, as supported by our sequencing data (data not shown).

In summary, VLA2001 is an adjuvanted, inactivated, whole-virus vaccine that has been developed using a well known manufacturing platform. This technology is used to make numerous vaccines that have been approved by regulatory authorities worldwide,^27^ and contains well established adjuvants that are known for creating a strong immune responses.^28^ VLA2001 showed a superior humoral response, a broader T-cell epitope coverage, and a superior tolerability and safety profile than ChAdOx1-S. It is the first and only inactivated whole-virus COVID-19 vaccine with marketing authorisation in the EU. Additionally, VLA2001 offers a tremendous advantage of straightforward refrigeration conditions for transportation and storage, reducing logistical complexities. We expect VLA2001 to contribute to the global effort to combat the COVID-19 pandemic through vaccination.

## Data sharing

Anonymised individual participant data will be made available when the study is complete, on reasonable requests made to the corresponding author. Proposals will be reviewed and approved by the sponsor, investigator, and collaborators on the basis of scientific merit. After approval of a proposal, data can be shared through a secure online platform. All data will be made available for a minimum of 5 years from the end of the trial.

For **WHO COVID-19 dashboard** see https://covid19.who.int/

## Declaration of interests

AF is a member of the Joint Committee on Vaccination and Immunisation, chair of the WHO European Technical Advisory Group of Experts on Immunisation, member of the WHO Working Group on COVID19 vaccines and is an investigator or provides consulting advice on clinical trials and studies of COVID-19 vaccines produced by AstraZeneca, Janssen, Valneva, Pfizer, Moderna, and Sanofi, and of other vaccines from these and other manufacturers, including GlaxoSmithKline, VPI Pharmaceuticals, Takeda, and Bionet Asia; he receives no personal remuneration or benefits for any of this work. ICR, KD, SD, SE-L, RH, JCJ, MK, BQ, CT, and PW are employees of Valneva Austria GmbH. RL has received grants or worked on clinical trials funded by Valneva, Moderna, Janssen, and AstraZeneca and has received support for meeting attendance, lectures, or writing from AstraZeneca.
