# Supplementary material for: Immunogenicity and safety of an inactivated whole-virus COVID-19 vaccine (VLA2001) compared with the adenoviral vector vaccine ChAdOx1-S in adults in the UK (COV-COMPARE): interim analysis of a randomised, controlled, phase 3, immunobridging trial
Source: Lancet Infect Dis. 2022 Dec;22(12):1716–27. doi: 10.1016/S1473-3099(22)00502-3 (PMC9444237; doi:10.1016/S1473-3099(22)00502-3)
Supplement: Supplementary appendix 1 [file mmc1.pdf]

THE LANCET
Infectious Diseases

Supplementary appendix 1

This appendix formed part of the original submission and has been peer reviewed. We post it as supplied by the authors.

Supplement to: Lazarus R, Querton B, Corbic Ramljak I, et al. Immunogenicity and safety of an inactivated whole-virus COVID-19 vaccine (VLA2001) compared with the adenoviral vector vaccine ChAdOx1-S in adults in the UK (COV-COMPARE): interim analysis of a randomised, controlled, phase 3, immunobridging trial. *Lancet Infect Dis* 2022; published online Sept 5. https://doi.org/10.1016/S1473-3099(22)00502-3.

Clinical Study Protocol

DRUG SUBSTANCE(S)	VLA2001
STUDY CODE	VLA2001-301
VERSION NO.	7.0
DATE	29 December 2021

A RANDOMIZED, OBSERVER-BLIND, CONTROLLED, SUPERIORITY STUDY TO COMPARE THE IMMUNOGENICITY AGAINST COVID-19, OF VLA2001 VACCINE TO AZD1222 VACCINE, IN ADULTS INCLUDING A RANDOMIZED, OBSERVER-BLIND, PLACEBO CONTROLLED PART IN ADOLESCENTS (≥12 TO <18 YEARS)

Short Title

COV-COMPARE Immunogenicity of vaccine VLA2001 compared to AZD1222.

Phase 3 study

PROTOCOL NUMBER: **VLA2001-301**

Sponsor

Valneva Austria GmbH
Phone: +43 1 20620
Fax: +43 1 20620 800
E-mail: info@valneva.com
Campus Vienna Biocenter 3
A-1030 Vienna, Austria

NOTE: The information contained in this document is the intelligence property of Valneva Austria GmbH. The privileged and confidential information is intended only for the use of the individual or entity to whom it has been sent or to whom it has been given by a member of Valneva's staff. If the reader of this document is not the intended recipient, you are hereby notified that any dissemination, distribution or copying of this item is strictly prohibited. If you have received this item in error, please notify us by telephone, and immediately return the original and all enclosures to us at Valneva Austria GmbH, Campus Vienna Biocenter 3, 1030 Vienna, Austria.

1. PROTOCOL SIGNATURE PAGE

A RANDOMIZED, OBSERVER-BLIND, CONTROLLED, SUPERIORITY STUDY TO COMPARE THE IMMUNOGENICITY AGAINST COVID-19, OF VLA2001 VACCINE TO AZD1222 VACCINE, IN ADULTS INCLUDING A RANDOMIZED, OBSERVER-BLIND, PLACEBO CONTROLLED PART IN ADOLESCENTS (≥12 TO <18 YEARS)

Protocol Number: VLA2001-301

With their signature, Investigators and the Sponsor agree to conduct this study in accordance with the Protocol, International Conference on Harmonization (ICH), Good Clinical Practice (GCP) guidelines and with the applicable local regulatory requirements. Moreover, the site will keep all information obtained from the participation in this study confidential unless otherwise agreed in writing.

**Name & Function**	**Signature**	**Date**
Principal Investigator		
Adam Finn, MD, PhD<br>Chief Investigator		
Michael Krammer<br>Clinical Operations, Valneva Austria GmbH		
Christian Taucher, PhD<br>Clinical Strategy, Valneva Austria GmbH		
Juan-Carlos Jaramillo, MD<br>Sponsor’s Responsible Medical Officer,<br>Valneva Austria GmbH		

2. STUDY PERSONNEL

Clinical Operations

Bénédicte Querton

Valneva Austria GmbH

Phone: +43 1 206 20 1275

Fax: +43 (0)1 20620 81275

e-mail: benedicte.querton@valneva.com

Sponsor's Medical / Safety Officer

Juan Carlos Jaramillo, MD

Valneva Austria GmbH

Phone: +43 1 20620 1106

Fax: +43 (0) 1 20620 81106

e-mail: juancarlos.jaramillo@valneva.com

Study Medical Monitor

Neda Vesic, MD
Mila Shuvalova, MD

Pharm-Olam International Deutschland GmbH

Phone: +49 89 3750 899 45 (49144)
Fax: +49 (0) 89 3750 899 20

Neda.Vesic@pharm-olam.com

2.1 Study Organization

The contact details of the organisation/individuals involved in the study (e.g.; Investigator(s), Sponsor's representative(s), laboratories, oversight committees (including ethics committees [ECs], as applicable) will be maintained by the Sponsor and provided to the Investigator.

3. ADVERSE EVENTS AND ADVERSE EVENTS OF SPECIAL INTEREST

The Investigator will comply with applicable laws/requirements for reporting serious adverse events (SAEs). For information on the definition and assessment of adverse events (AEs), refer to Section 15.8.1.

All adverse events of special interest (AESIs) require expedited reporting, similar to SAEs. For information on the definition of AESIs, refer to Section 15.8.1.

All SAEs and AESIs should be reported on the SAE/AESI Report Form in the electronic case report form (eCRF) within 24 hours of the Investigator becoming aware of the event. Under certain circumstances, the initial notification could be done by phone; nevertheless, a written SAE/AESI Report Form has to be submitted within 24 hours to:

Pharm-Olam International	
Fax:	+44 (0)208 338 0380
Email:	POISafetyDesk@pharm-olam.com
Safety Hotline:	+44 (0) 808 189 3104

4. PREGNANCY REPORTING

The Investigator will comply with applicable laws/requirements for reporting pregnancies. For information on the definition and assessment of pregnancies, refer to Section 15.8.2.

All Pregnancies* should be reported on the Pregnancy Report Form to Pharm-Olam International by fax or email within 24 hours of the Investigator becoming aware of the event.

Safety Desk	
Fax:	+44 (0)208 338 0380
Email:	POISafetyDesk@pharm-olam.com
Safety Hotline:	+44 (0) 808 189 3104

* A pregnancy is not considered an SAE. If a seriousness criterion applies in addition to the pregnancy (e.g., hospitalisation, congenital anomaly/birth defect) the pregnancy qualifies as an SAE. In such case a Pregnancy Report Form **and** an SAE Report Form have to be filled out.

5. CLINICAL STUDY SYNOPSIS

**TITLE:**	A Randomized, Observer-blind, Controlled, Superiority Study to Compare the Immunogenicity against COVID-19, of VLA2001 Vaccine to AZD1222 Vaccine, in Adults including a Randomized, Observer-Blind, Placebo Controlled Part in Adolescents (≥12 to <18 years).
**SHORT TITLE:**	**COV-COMPARE** Superiority of Immunogenicity of vaccine VLA2001 compared to AZD1222.
**PHASE:**	3
**PRIMARY OBJECTIVES:**	**Immunogenicity**:<br>1) To demonstrate the superiority of VLA2001 (Wuhan strain) compared to AZD1222 administered in a 2-dose immunization schedule 4 weeks apart, in terms of Geometric Mean Titre (GMT) ratio as well as non-inferiority in terms of seroconversion rate, of neutralising antibodies at 2 weeks after the second vaccination (Day 43) in adults aged 30 years and older.<br>2) To examine the immunogenicity of VLA2001 administered in a 2-dose immunization schedule 4 weeks apart, in terms of GMT and non-inferiority in terms of seroconversion rate, of neutralising antibodies at 2 weeks after the second vaccination (Day 43) in adolescents aged ≥12 to <18 years compared to adults 18-29 years of age.<br>**Safety**:<br>3) To evaluate the safety and tolerability of VLA2001 at 2 weeks after the second vaccination (Day 43) in adults and adolescents aged ≥12 years and older.
**SECONDARY OBJECTIVES:**	**Immunogenicity:**<br>1) To assess immunogenicity of a 2-dose primary immunization schedule with VLA2001 in adults aged 18 years and above.<br>2) To assess immunogenicity of a 2-dose primary immunization schedule with VLA2001 in adolescents aged ≥12 to <18 years.<br>3) To determine the immunogenicity of a single booster dose with VLA2001 in adults and adolescents.<br>4) To evaluate cellular immune responses following administration of VLA2001 in adults and adolescents.<br>**Safety:**<br>5) To compare the safety of VLA2001 to AZD1222 up to Day 43 in adults aged 30 years and older, following a 2-dose immunization schedule 4 weeks apart.<br>6) To compare the safety of VLA2001 to placebo up to Day 71 in adolescents aged ≥12 to <18 years, following 2-dose primary immunization with VLA2001.<br>7) To describe the safety and tolerability of VLA2001 in adults who received 2 doses of VLA2001 up to 12 months after the first vaccination.

	8) To describe the safety and tolerability of VLA2001 in adolescents aged ≥12 to <18 years following a 2-dose primary immunization schedule with VLA2001 up to 12 months after second vaccination.<br>9) To determine the safety and tolerability of a single booster dose with VLA2001 in adults and adolescents up to 6 months post booster dose.
**EXPLORATORY OBJECTIVE:**	1) To assess the efficacy of VLA2001 in the prevention of COVID-19 in an adult population, aged 30 years and older.<br>2) To assess the efficacy of VLA2001 in the prevention of COVID-19 in an adolescent population, aged ≥12 to <18 years.
**PRIMARY ENDPOINTS:**	**Immunogenicity:**<br>1) Immune response measured after completion of a 2-dose immunization schedule with VLA2001, as determined by the GMT ratio in adults and GMT in adolescents, as well as seroconversion (defined as 4-fold increase from baseline) of SARS-CoV-2-specific neutralising antibodies on Day 43.<br>**Safety:**<br>2) Frequency and severity of any Adverse Events (AE) up to Day 43 post-vaccination with VLA2001.
**SECONDARY ENDPOINTS**:	1. Proportion of adult participants with seroconversion after receipt of 2 doses of study vaccination on Day 8 (age 55+ only), Day 29, Day 71, Day 208. Seroconversion is defined as ≥ 4-fold increase in SARS-CoV-2 neutralising antibody titre against the Wuhan strain and IgG antibodies directed against the S-protein of the Wuhan strain between Day 1 and the defined post-vaccination time points.<br>2. Proportion of adolescents, aged ≥12 to <18 years with seroconversion following a 2-dose primary immunization with VLA2001 at visits V3a and V4ab as well as V6p and V8p<br>3. Immune response in adults on Day 8 (age 55+ only), Day 29, Day 71, and Day 208, as determined by the GMT of SARS-CoV-2-specific neutralising antibodies.<br>4. Immune response in adolescents, aged ≥12 to <18 years, as determined by the GMT of SARS-CoV-2-specific neutralising antibodies following a 2-dose immunization schedule with VLA2001 at V3a and V4ab as well as V6p and V8p.<br>5. GMT ratio of SARS-CoV-2-specific neutralizing antibodies in the adolescent population vaccinated with VLA2001 at Day 43 (i.e. Visit 3a) and the adult population above 30 years (i.e. Visit 4).<br>6. Immune response in adults (age 55+ only) on Day 8 (age 55+ only), Day 29, Day 43, Day 71 and Day 208 , as determined by the GMT of IgG antibodies to SARS-CoV-2 S-protein.<br>7. Immune response in adolescents aged ≥12 to <18 years, as determined by the GMT of IgG antibodies to SARS-CoV-2 S-protein following a 2-dose immunization schedule with VLA2001 at V3a and V4ab as well as V6p and V8p.

	8. GMT ratio of IgG antibodies to SARS-CoV-2 S-protein in the adolescent population vaccinated with VLA2001 at Day 43 (i.e. Visit 3a) and the adult population above 30 years (i.e. Visit 4).<br>9. Assessment of T-cell responses (Th1/Th2 polarization) from PBMCs on selected time points in a subset of participants after in vitro stimulation with SARS-CoV-2 antigens using e.g. ELISpot (IFNγ) or intracellular cytokine staining (IL-2, IL-4, IL-5, IL-13, TNFα, IFNγ)<br>**Safety:**<br>10. Frequency and severity of solicited injection site and systemic reactions within 7 days after each and after any vaccination (primary vaccination series).<br>11. Frequency and severity of any AE during the entire study period.<br>12. Frequency and severity of any unsolicited AE following the completion of the 2-dose immunization schedule with VLA2001.<br>13. Frequency and severity of any unsolicited vaccine-related AE following the completion of the 2-dose immunization schedule with VLA2001.<br>14. Frequency and severity of any serious adverse event (SAE) during the entire study period.<br>15. Frequency and severity of any adverse event of special interest (AESI) during the entire study period.<br>**Additional immunogenicity endpoints for the subset of adult participants who received a single Booster dose of VLA2001:**<br>B1) Geometric mean fold rise (GMFR) from pre-booster time point (day of booster vaccination, Visit B1) to 14 days (Visit B2) after booster dose with regards to SARS-CoV-2-specific neutralizing antibodies.<br>B2) GMT of SARS-CoV-2-specific neutralizing antibodies as measured by MNA50, at Visits B1, B2 and B3 including formal non-inferiority testing on the GMT ratio Visit 4/ Visit B2 for the booster subgroup who had received 2 doses of VLA2001 for primary immunization schedule.<br>B3) Proportion of participants with 4-fold increase from pre-booster time point (day of booster vaccination, Visit B1) to 14 days (Visit B2) after booster dose with regards to SARS-CoV-2-specific neutralizing antibodies.<br>B4) GMFR from pre-booster time point (day of booster vaccination, Visit B1) to 14 days (Visit B2) after booster dose with regards to S-protein binding antibodies.<br>B5) Proportion of participants with 4-fold increase from pre-booster time point (day of booster vaccination, Visit B1) to 14 days (Visit B2) after booster dose with regards to S-protein binding antibodies.<br>B6) GMT measured as IgG antibodies against SARS-CoV-2 as determined by ELISA, at Visits B1, B2 and B3.

	B7) Assessment of T-cell responses from PBMCs in a subset of participants after in vitro stimulation with SARS-CoV-2 antigens using ELISpot (IFNγ) at Visits B1, B2 and B3.<br><br>**Additional Safety endpoints for adult booster dose group:**<br>B8) Frequency and severity of solicited injection site and systemic reactions within 7 days after booster vaccination in a subset of participants.<br>B9) Frequency and severity of any unsolicited AE up to 6 months after booster dose.<br>B10) Frequency and severity of any vaccine-related up to 6 months after booster dose.<br>B11) Frequency and severity of any serious adverse event (SAE) up to 6 months after booster dose.<br>B12) Frequency and severity of any adverse event of special interest (AESI) up to 6 months after booster dose.<br><br>**Additional immunogenicity endpoints for the subset of adolescent participants who received a single Booster dose of VLA2001:**<br>B1) Geometric mean fold rise (GMFR) from pre-booster time point (day of booster vaccination, V5ab) to 14 days after booster dose (V6ab) with regards to SARS-CoV-2-specific neutralizing antibodies.<br>B2) GMT of SARS-CoV-2-specific neutralizing antibodies as measured by MNA50, at Visits V5ab, V6ab and V7ab<br>B3) Proportion of participants with 4-fold increase from pre-booster time point (day of booster vaccination, V5ab) to 14 days after booster dose (V6ab) with regards to SARS-CoV-2-specific neutralizing antibodies.<br>B4) GMFR from pre-booster time point (day of booster vaccination, V5ab) to 14 days after booster dose (V6ab) with regards to S-protein binding antibodies.<br>B5) Proportion of participants with 4-fold increase from pre-booster time point (day of booster vaccination, V5ab) to 14 days after booster dose (V6ab) with regards to S-protein binding antibodies.<br>B6) GMT measured as IgG antibodies against SARS-CoV-2 as determined by ELISA, at Visits V5ab, V6ab and V7ab<br>B7) Assessment of T-cell responses from PBMCs in a subset of participants after in vitro stimulation with SARS-CoV-2 antigens using ELISpot (IFNγ) at Visits V5ab, V6ab and V7ab.

	**Additional Safety endpoints for adolescent booster dose group:**<br>B8) Frequency and severity of solicited injection site and systemic reactions within 7 days after booster vaccination<br>B9) Frequency and severity of any unsolicited AE up to 6 months after booster dose.<br>B10) Frequency and severity of any vaccine-related up to 6 months after booster dose.<br>B11) Frequency and severity of any serious adverse event (SAE) up to 6 months after booster dose.<br>B12) Frequency and severity of any adverse event of special interest (AESI) up to 6 months after booster dose.
**EXPLORATORY ENDPOINTS**:	1) Occurrence of PCR confirmed SARS-CoV-2 infection starting from 2 weeks after the second dose

STUDY DESIGN:

This is a Randomized, Observer-blind, Controlled, Superiority Study to compare the immunogenicity of VLA2001 to AZD1222 in terms of GMT of SARS-CoV-2-specific neutralising antibodies. Furthermore, VLA2001 will be compared to placebo in an adolescent population.

Figure 1: Study Overview - Adults

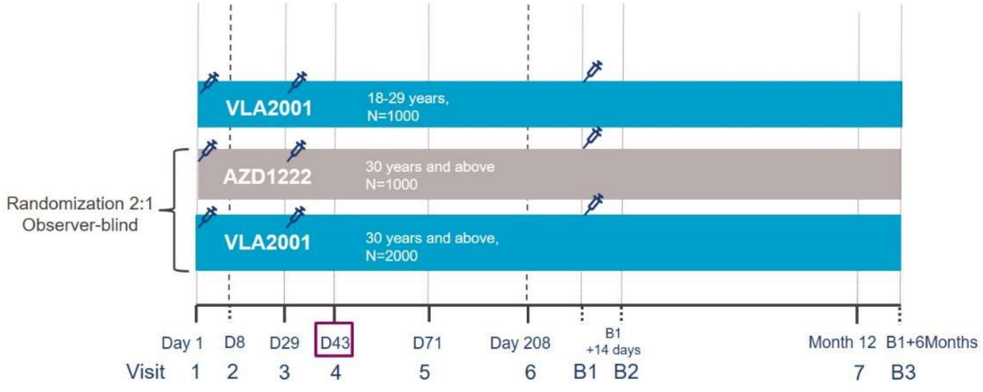

Note: For adults Day 8 visit is only applicable for participants of the immunogenicity subset.

Adult Population

In the adult population, approximately 4,000 adults will be recruited into the study. Participants will be adults aged ≥18 years older who are either generally healthy or are with a stable medical condition. Immunogenicity and safety of VLA2001 will be assessed up to Month 12 after the first vaccination.

Approximately 3,000 participants ≥30 years of age and older will be randomized overall in a 2:1 ratio to receive 2 intramuscular doses of either VLA2001 (n=2,000) or AZD1222 (n=1,000) at the recommended dose level, 28 days apart, on Days 1 and 29.

For immunogenicity analyses, samples from approximately 1,200 adult participants (600 per group) who have been tested sero-negative for SARS-CoV-2 at screening will be analysed.

Approximately 1,000 participants that are under 30 years of age will be placed in a non-randomized treatment group and receive VLA2001 at the recommended dose level, 28 days apart, on Days 1 and 29.

The first 10 participants who are older than 55 years at the time of signing the ICF, will be treated as a ‘sentinel’ group and will be observed for at least 60 minutes after each vaccination. Thereafter, participants will be observed for immediate AEs and/or reactogenicity for at least 30 minutes after the administration of the vaccine.

VLA2001 Booster Group in Adults

All participants – except those who already received a licensed COVID-19 vaccine outside of the study - will be offered a booster dose with VLA2001. All eligible and willing participants will receive a booster vaccination with VLA2001at Visit B1 and will have a follow-up visit 14 days (Visit B2) after the booster dose. The participants will have 1 more follow-up visit 6 months after the booster vaccination (i.e., Visit B3 which replaces Visit 7(Month 12) for those participants who received a booster dose). All participants who do not receive a booster dose with VLA2001 will continue with Visit 7 (Month 12).

	Blood samples for immunogenicity analysis will be taken in a subset of adults who had received the primary vaccination with 2 doses of VLA2001 (N=330) as well as a subset of participants who had received 2 doses of AZD1222 for primary immunization (N=110). The timing of the booster dose administration will be scheduled after Visit 6 (Day 208) approximately between January and February 2022.<br><br>**Adolescent Population**<br><br>For safety reasons, the first 16 adolescents will be enrolled in an open label, non-randomized manner. The sentinel dosing is aimed to be done at a single site to ensure permanent oversight on safety data by one principal investigator during the recruitment of the 16 sentinel participants.<br><br>If, for logistical reasons, a second site needs to be involved in the recruitment of the sentinel participants, vaccinations will be limited to one site on a specific day. Safety data exchange between the study sites will be ensured.<br><br>The first 4 adolescents will be vaccinated 1 hour apart each. After vaccination, the participant will be observed for the development of any acute reaction at the study site for 3 hours after the vaccination procedure. Prior to discharge from the study site, vital signs will be measured and the participant/parents will be instructed in the use of the eDiary. The study site will contact the participant by phone approximately 24 and 48 hours after vaccination for safety follow-up. The provided information provided must be compared with the entries in the participant´s eDiary.<br><br>The minimum time before the next 4 participants are vaccinated is 24 hours after all participants of the previous sentinel cohort have received their vaccination. The same procedure is applied for the following 3 sentinel groups.<br><br>A Data Safety and Monitoring Board (DSMB) will review the accrued safety data when all 16 sentinel participants have completed the 7-day e-diary period after vaccination. After favourable DSMB review, randomization of the remaining participants across all sites will be initiated. Ad-hoc DSMB reviews will be performed if individual withdrawal criteria are met (section 11.6.1).<br><br>Subsequently, at least 660 participants will be recruited and randomized in a 1:1 ratio to receive 2 intramuscular doses of either VLA2001 (n=330) or placebo (n=330). Adolescents randomized to receive VLA2001, will receive a booster vaccination with VLA2001 at Visit V5ab (Day 208). Participants in the placebo-group will receive a 2-dose primary immunization (28 days apart) with VLA2001 at Visit V4p and Visit V5p (Figure 2).

Figure 2 - Study Overview - Adolescents

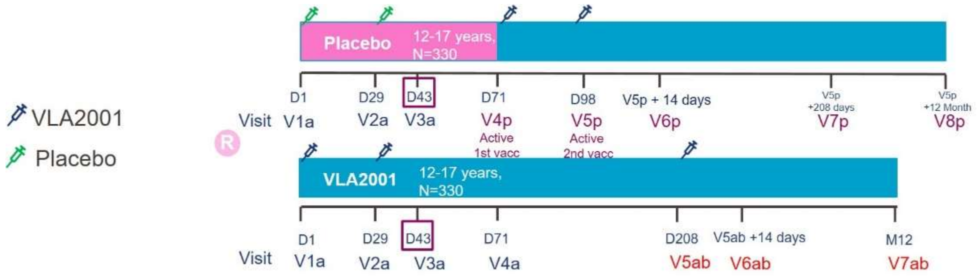

Due to the fact that the placebo group will receive a dose of VLA2001 at Visit V4p, all adolescent participants will be unblinded at this point in time. Visit V5p, V6p, V7p and V8p will only be performed by the participants initially randomized to the placebo-group.

VLA2001 Booster Group in Adolescents

Adolescents initially randomized to receive the primary vaccination schedule with VLA2001 will receive a booster vaccination with VLA2001 at Visit V5ab and will have a follow-up visit 14 days after the booster dose (i.e., Visit V6ab). The participants will have 1 more follow-up visit 5 months after the booster vaccination (i.e. Visit V7ab).

Applicable for all participants and study arms:

All visits will be conducted at the clinical site on an outpatient basis.

Initial vaccination with VLA2001 (28 days apart)

Participants will be provided with an electronic Diary (e-Diary) and will be trained to record specifically solicited systemic and local symptoms daily, as well as any additional AEs during the follow-up period after each of both vaccinations up to the next visit to the site, i.e. Day 43 visit has been completed. Adolescents in the VLA2001-group will be asked to complete their e-Diary until Visit V6ab (i.e., the next site visit following VLA2001-booster vaccination), and adolescents in the placebo-group until Visit V6p (i.e., the next site visit following the second vaccination with VLA2001).

Booster vaccination with VLA2001

Participants will be provided with an electronic Diary (e-Diary) and will be trained to record specifically solicited systemic and local symptoms daily as well as any additional AEs during follow-up period after booster vaccination up to the next visit to the site has been completed.

The following information will be collected:

- Oral body temperature,
- Solicited local (i.e., injection site) reactions (predefined terms),
- Solicited systemic reactions (predefined terms),
- Other AEs, i.e. unsolicited events
- Any new medications or changes in medication administered after vaccination

**INCLUSION CRITERIA:**	Participants must meet **all** inclusion criteria to be eligible for the study.<br>1. All participants must have read, understood, and signed the informed consent form (ICF),<br>a. For participants aged 16 years or older: Written informed consent prior to any study related procedures<br>b. For participants aged ≥12 to <16 years: Written informed consent by the participant's legal representative(s), according to local requirements, and written informed assent of the participant, if applicable, prior to any study related procedures.<br>2. Participants of either gender aged 12 years and older at screening.<br>3. Medically stable such that, according to the judgment of the investigator, hospitalization within the study period is not anticipated and the participant appears likely to be able to remain on study through the end of protocol-specified follow-up,<br>• A stable medical condition is defined as disease not requiring significant change in therapy or hospitalization for worsening disease during the 3 months prior to the expected day of randomization (Visit 1).<br>4. Must be able to attend all visits of the study and comply with all study procedures, including daily completion of the e-Diary for 7 days after each vaccination,<br>5. Women of childbearing potential (WOCBP) who are sexually active with a man must be able and willing to use at least 1 highly effective method of contraception (i.e. including hysterectomy, bilateral salpingectomy, and bilateral oophorectomy, hormonal oral medication, male condoms with spermicide, transdermal, implant or injection, barrier (i.e., condom, diaphragm with spermicide); intrauterine device; vasectomized partner (6 months minimum), clinically sterile partner, or abstinence) for a minimum of 3 months after the last dose of study vaccine,<br>• A female participant is considered to be a WOCBP after menarche and until she is in a postmenopausal state for 12 consecutive months (without an alternative medical cause) or otherwise permanently sterile.<br>Note: Participants not of childbearing potential are not required to use any other forms of contraception during the study. Non-childbearing potential is defined as participant confirmed.<br>• Surgical sterilization (e.g., bilateral oophorectomy, bilateral salpingectomy, bilateral occlusion by cautery, hysterectomy, or tubal ligation),<br>• Postmenopausal (defined as permanent cessation of menstruation for at least 12 consecutive months prior to screening).<br>6. WOCBPs must have a negative pregnancy test prior to each vaccination.

**EXCLUSION CRITERIA:**	Participants will not be eligible for the study if they meet any of the exclusion criteria, or will be discontinued from study vaccination if they develop any of the following exclusion criteria during the study:<br>1. Participant is pregnant or planning to become pregnant within 3 months after study vaccine administration,<br>2. History of allergy to any component of the vaccine,<br>3. Significant infection (e.g., positive SARS-CoV-2 RT-PCR) or other acute illness, including fever >100 °F (>37.8 °C) 48 hours before vaccination,<br>4. Participant has a known or suspected defect of the immune system, such as participants with congenital or acquired immunodeficiency, including infection with HIV, status post-organ transplantation or immunosuppressive therapy within 4 weeks prior to the expected day of randomization (Visit 1),<br>• Immunosuppressive therapy is defined as administration of chronic (longer than 14 days) prednisone or equivalent ≥0.05 mg/kg/day within 4 weeks prior to the expected day of randomization (Visit 1), radiation therapy or immunosuppressive cytotoxic drugs/monoclonal antibodies in the previous 3 years; topical and inhaled steroids are allowed.<br>• Participants with chronic HIV unless: HIV disease with documented viral load <50 copies/mL and CD4 count >200 cells/mm3 for at least 6 months before enrolment, and stable antiretroviral therapy for the last 6 months.<br>5. Participant has a history of cerebral venous sinus thrombosis, heparin-induced thrombocytopenia, or antiphospholipid syndrome,<br>6. Participant has a history of malignancy in the past 5 years other than squamous cell or basal cell skin cancer. If there has been surgical excision or treatment >5 years ago, that is considered to have achieved a cure, the participant may be enrolled. A history of hematologic malignancy is a permanent exclusion. Participants with a history of skin cancer must not be vaccinated at the previous tumour site,<br>7. History of drug dependency or current drug or alcohol abuse at screening,<br>8. Significant blood loss (>450 mL) or has donated 1 or more units of blood or plasma within 6 weeks prior to the expected day of randomization (Visit 1) or the booster administration (Visit B1).<br>9. History of clinically significant bleeding disorder (e.g., factor deficiency, coagulopathy, or platelet disorder), or prior history of significant bleeding or bruising following IM injections or venepuncture.<br>10. Severe and uncontrolled ongoing autoimmune or inflammatory disease, history of Guillain-Barre syndrome, or any other demyelinating condition<br>11. Any other significant disease, disorder, or finding which, in the opinion of the investigator, may significantly increase the risk to

	the volunteer because of participation in the study, affect the ability of the volunteer to participate in the study, or impair interpretation of the study.<br><br>Prior/concomitant therapy:<br><br>12. Receipt of immunoglobulin or another blood product within the 3 months before expected day of randomization (Visit 1) or the booster administration (Visit B1). in this study or those who expect to receive immunoglobulin or another blood product during this study,<br>13. Receipt of medications and or vaccinations (other than the study vaccinations) intended to prevent COVID-19,<br>14. Receipt of any vaccine (licensed or investigational), other than licensed influenza vaccine, within 28 days prior to the expected day of randomization (Visit 1).<br><br>Others:<br><br>15. Any member of the study team or sponsor,<br>16. An immediate family member or household member of the study's personnel.<br><br>**Booster Vaccination (Adults and Adolescent)**<br><br>In addition to the above described eligibility criteria, the following criteria must be met:<br><br>1. Participant has not received another licensed COVID-19 vaccine during the study
**DELAY CRITERIA FOR VACCINATION**	Vaccination will be delayed if:<br><br>1. Participant has an acute infection within 48 hours prior to vaccination or oral temperature ≥ 38.0 °C on the day of vaccination;<br>2. Participant has received antipyretics within 4 hours prior to the scheduled time of vaccination.<br>3. Participant has received any other vaccine (other than licensed influenza vaccine or for medical emergencies such as tetanus or rabies exposure), within 28 days.<br><br>The rescheduled visit should be within the specified time window for the vaccination visit. In case of a delay of the second vaccination beyond the pre-specified time window this must be captured as protocol deviation.<br><br>In any case, all efforts should be made to schedule the next vaccination as soon as possible after the participant is eligible for receiving it.<br><br>In addition, for a rescheduled vaccination all inclusion and none of the exclusion criteria must be met; in case not all of these criteria are met, the vaccination will be omitted.
**INVESTIGATORS AND SITES:**	This is a multi-centre study. The study will be conducted at approximately 25 study sites in the United Kingdom, Germany and Mexico.

**SAMPLE SIZE**	The sample size for the adult part of this study is selected in order to establish a comprehensive safety database and to characterize the safety profile of VLA2001. 3,000 participants vaccinated with VLA2001 will allow for the detection of at least 1 rare event (incidence rate 1/1000) with a probability of 94% in this study. The VLA2001 safety database will then provide safety data on more than 3000 vaccinated adult participants.<br><br>The planned number of 600 participants per group in the immunogenicity subset will allow for a statistical power of 90% to detect superiority in terms of the Day 43 GMT ratio VLA2001/AZD1222 with an expected ratio of 1.3, a standard deviation of 0.6 (on a LOG10 scale), expected drop-out rate of 10%, and a two-sided significance level of 5%.<br><br>For the adolescent part of the study, a formal sample size calculation has not been performed as there will be no formal hypothesis testing. However, approximately 330 adolescents vaccinated with VLA2001 is considered to provide sufficient immunogenicity data to support immune-bridging to the adult population.
**STUDY TYPE**	Superiority of immunogenicity.
**INVESTIGATED THERAPY:**	VLA2001: Inactivated, SARS-CoV-2 (Wuhan) vaccine adjuvanted with CpG and Alum (33AU/dose, volume of one dose is 0.5mL).
**REFERENCE THERAPY:**	For adult part: AZD1222: A recombinant, replication-defective chimpanzee adenovirus expressing the SARS-CoV-2 S surface glycoprotein driven by the human cytomegalovirus major immediate early promoter that includes intron A with a human tPA leader sequence at the N terminus.
**STUDY INITIATION:**	Q2-2021
**STUDY DURATION:**	The overall study duration (First Participant First Visit [screened] – Last Participant Last Visit [LSO]) is estimated to be approximately 21 months.<br><br>The total study duration for the individual participant (adults and adolescents) is up to 16 months from the day of signing the informed consent to study completion, unless prematurely discontinued.
**STUDY COMPLETION:**	Last Participant Last Visit in the adult population is expected to occur by Aug-2022.<br><br>Last Participant Last Visit in the adolescent population is expected to occur in Q2 2023.
**STATISTICS:**	<u>**Analysis Populations:**</u><br><br>The **Safety population** contains all participants who entered into the study and received at least one study vaccination. Participants will be analysed as treated.<br><br>The **Booster safety population** for adult and adolescent participants will contain all participants who received a booster dose.<br><br>The **Immunogenicity (IMM) population** is defined to include all randomized and vaccinated participants of the IMM subset for the primary endpoint evaluation, who were SARS-CoV-2 seronegative and have at least one evaluable post-baseline antibody titer measurement after vaccination. Participants who meet the case definition of confirmed COVID-19 during the study will not be included in the primary endpoint evaluation.

The **PBMC population** will be a randomized subset of the immunogenicity population (participants aged 30 years and above). 200 adult participants (100 adult participants of the VLA2001 immunogenicity subset and 100 adult participants of the AZD1222 immunogenicity subset) will randomized for PBMC sample collection and analysis.

In addition, 100 adolescent participants (50 adolescent participants of the VLA2001 and 50 adolescent participants of the placebo group) will be randomized for PBMC sample collection and analysis.

The **Per Protocol (PP) population** contains all IMM population participants who have no major protocol violations that could impact the immune response.

The **Booster Immunogenicity population** will contain a subset of participants who received a booster dose with VLA2001 and from which blood samples are drawn. It is planned to include:
- approximately 330 adult participants who have received 2 doses of VLA2001 for primary immunization
- approximately 110 participants who had received 2 doses of AZD1222 for primary immunization and
- approximately 50 adolescent participants who have received 2 doses of VLA2001 for primary immunization

Selection of subjects for the immunogenicity blood sampling is described in section 10.5.1.

Analysis of T-cell response will be done for those participants from whom PBMC samples are available (see section 10.5.1).

Immunogenicity Analysis:

The primary immunogenicity endpoint (Day 43 GMT) will be compared between groups using a t-test applied to neutralization titres after LOG10 transformation. An analysis of variance with the factors study site and treatment group will be applied secondarily for sensitivity analysis.

Safety Analysis:

Vital signs, clinical laboratory tests, and physical examination findings will be listed and summarized by time point using appropriate descriptive statistics.

The number and percentage of participants reporting any Adverse event or reactogenicity will be tabulated by system organ class and preferred term (coded using MedDRA). All Adverse Events will be recorded appropriately. All SAEs and AESIs will be analysed and presented by severity and relationship to study treatment up to the end of study (Month 12) or up to 6 Months post booster dose.

Analysis for Study Endpoints

A first statistical analysis, including the analysis of the superiority primary endpoint, will be performed after the last adult participant has completed the Day 43 visit.

	A second statistical analysis will be performed when all adult participants have completed Visit 6 (Day 208) and the adult participants from the booster immunogenicity subset have completed visit B2.<br><br>A third statistical analysis will be performed when all adolescent participants have completed Visit V3a.<br><br>A fourth statistical analysis will be performed at the time when adolescent participants have completed Visits V6p and V6ab.<br><br>A final analysis will be performed once the last participant has completed the last visit.
**MONITORING OF COVID-19 SYMPTOMS**	Participants who develop any of the COVID-19 related symptoms after randomization into the study will be advised to contact the study team for evaluation and testing for SARS-CoV-2 (PCR) according to the following scheme:<br><br>**Immediately:** Participants should contact the study site immediately; in case they develop any of the following symptoms:<br>1. Fever (≥38.0°C or ≥100.4°F), or<br>2. Shortness of breath, or<br>3. Difficulty in breathing (wheezing, croup).<br><br>**After 2 consecutive days:** Participants should contact the study site in case they have at least one of the following symptoms for at least 2 consecutive days:<br>• Sore throat,<br>• Chills,<br>• Cough,<br>• Fatigue,<br>• Muscle aches,<br>• Body aches,<br>• Headache,<br>• New loss of taste,<br>• New loss of smell,<br>• Nasal Congestion,<br>• Runny nose,<br>• Nausea,<br>• Vomiting,<br>• Diarrhoea.<br><br>In case of an initial negative PCR test result, participants will be invited for a second confirmatory PCR test on site after approximately 2 days. If the test can not be done at site, a test in the community is also acceptable. In the event the result is still negative, the participant will continue with scheduled visits.<br><br>If any of the tests is positive, the participant will be asked to come for a COVID-19 illness visit at site (if the national quarantine protocol allows it). Where necessary and possible, the COVID-19 illness visit may be substituted by a home or mobile visit.

	As part of the illness visit, blood samples will be taken for antibody assessment. Either a nasal swab or gargle (saliva) sample will be taken for genetic sequencing for identification of the virus strain. In addition, a recording of COVID-19-related clinical and laboratory information as shown in the Schedule of Assessments will be done.<br><br>The Investigator's severity grading of COVID-19 will be based on the following case definition criteria:<br><br>**Case Definition for Mild COVID-19**<br><br>A SARS-CoV-2 positive RT-PCR result<br><br>AND:<br><br>One of the following symptoms:<br><br>• Fever (≥38.0°C or ≥100.4°F),<br>• Sore throat,<br>• Malaise (loss of appetite, generally unwell, fatigue, physical weakness),<br>• Pain (headache, muscle pain (myalgia)),<br>• gastrointestinal symptoms,<br>• Respiratory symptoms (cough, chest congestion, runny nose, wheezing),<br>• Skin rash,<br>• Eye irritation or discharge,<br>• Shaking, Chills, or rigors<br>• New or changing olfactory or taste disorders,<br>• Red or bruised looking feet or toes.<br><br>**Case Definition for Moderate COVID-19**<br><br>A SARS-CoV-2 positive RT-PCR result<br><br>AND<br><br>Any 1 of the following new or worsening signs or symptoms:<br><br>• Respiratory rate ≥20 breaths/minute,<br>• Abnormal saturation of oxygen (SpO_2) but still >93% on room air at sea level or adjusted according to altitude,<br>• Clinical or radiologic evidence of pneumonia,<br>• Radiologic evidence of deep vein thrombosis (DVT)<br>• Shortness of breath or difficulty breathing.<br><br>OR<br><br>Any 2 of the following new or worsening signs or symptoms:<br><br>• Fever (≥38.0°C or ≥100.4°F),

- Heart rate ≥90 beats/minute,
- Shaking, chills, or rigors,
- Sore throat,
- Cough,
- Malaise, as evidenced by 1 or more of the following:
  - Loss of appetite,
  - Generally unwell,
  - Fatigue,
  - Physical weakness.
- Headache,
- Muscle pain (myalgia),
- Gastrointestinal symptoms (e.g. diarrhoea, vomiting, nausea, abdominal pain)
- New or changing olfactory or taste disorders
- Red or bruised looking feet or toes.

Having 2 or more elements of a symptom (e.g., vomiting and diarrhoea or fatigue and loss of appetite) is counted only as 1 symptom for the case definition. To meet the case definition, a participant would need to have at least 2 different symptoms.

Case Definition for Severe/Critical COVID-19

A SARS-CoV-2 positive RT-PCR result

AND

Any 1 of the following at any time during the course of observation:

- Clinical signs at rest indicative of severe systemic illness (respiratory rate ≥30 breaths/minute, heart rate ≥125 beats/minute, oxygen saturation (SpO_2) ≤93% on room air at sea level, or partial pressure of oxygen/fraction of inspired oxygen (PaO_2/FiO_2) <300 mmHg)

  (SpO_2 criteria will be adjusted according to altitude per the investigator judgement),
- Respiratory failure (defined as needing high-flow oxygen, non-invasive ventilation, mechanical ventilation, or extracorporeal membrane oxygenation [ECMO]),
- Evidence of shock (defined as systolic blood pressure <90 mmHg, diastolic blood pressure <60 mmHg, or requiring vasopressors),
- Significant acute renal, hepatic, or neurologic dysfunction,
- Admission to the ICU,
- Death.

All cases meeting the severe/critical criteria will be reviewed by the Data and Safety Monitoring Board (DSMB) to determine if the case is severe/critical in their judgement.

**ACCESS TO NATIONALLY DEPLOYED OR APPROVED COVID-19 VACCINE**	It is possible that during the course of this study, participants become eligible, through the national vaccination roll-out, to receive a nationally deployed or approved COVID-19 vaccine (this then becomes standard of care vaccine). Participants can discuss with the Investigator and others to make an informed choice about whether they should take the approved COVID-19 vaccine or continue with the study.<br><br>If a participant decides to receive a nationally deployed COVID-19 vaccine, and the Investigator may unblind the participant and inform them about which vaccine they have received if this will be required for booster administration. The Investigator will also advise them on consequences and the necessity to complete the study visits according to the schedule of study procedures and assessments.<br><br>If the study participant has already received the 2 doses of the study vaccine, they will not be unblinded immediately but advised to wait until after Day 43 visit. Participants who decide to take a nationally deployed COVID-19 vaccine will be encouraged to still attend all remaining study visits and procedures. However, if the participant decides not to attend the remaining study visits, they will be asked to perform an Early Termination visit, as stated in the Schedule of Study Procedures and Assessments of this Protocol.
**DATA AND SAFETY MANAGEMENT BOARD**	An Independent Data and Safety Management Board (DSMB) will be constituted for this study.
**SPONSOR**	VALNEVA Austria GmbH

6. TABLE OF CONTENTS

1. PROTOCOL SIGNATURE PAGE 2

2. STUDY PERSONNEL 3

2.1 Study Organization 3

3. ADVERSE EVENTS AND ADVERSE EVENTS OF SPECIAL INTEREST 4

4. PREGNANCY REPORTING 5

5. CLINICAL STUDY SYNOPSIS 6

Analysis of T-cell response will be done for those participants from whom PBMC samples are available (see section 10.5.1). 18

6. TABLE OF CONTENTS 23

7. LIST OF ABBREVIATIONS 27

8. INTRODUCTION 29

8.1 Clinical Condition/Indication 29

8.1.1 Transmission, Disease and Diagnosis 29

8.1.2 Prospects for Vaccine Development 30

8.2 Valneva's Candidate Vaccine: VLA2001 30

8.3 Findings from Nonclinical and Clinical Studies 30

8.3.1 Non-clinical Summary 30

8.3.2 Clinical Summary 31

8.4 Study Rationale 32

8.4.1 The Comparator vaccine: AZD1222 32

8.4.2 Justification for the choice of comparator 33

8.4.3 Possible Benefits for the Participant 34

8.4.4 Possible Benefits for Society 35

8.4.5 Possible Risks/Inconveniences for the Participant 35

9. STUDY PURPOSE AND OBJECTIVES 37

9.1 Study Purpose 37

9.2 Primary Objectives (Immunogenicity and Safety) 37

9.3 Secondary Objectives (Immunogenicity and Safety) 37

9.4 Exploratory Objectives (Efficacy) 38

9.5 Study Endpoints 38

9.5.1 Primary Endpoints 38

9.5.2 Secondary Endpoints 38

9.5.3 Exploratory endpoint 41

10. STUDY DESIGN 42

10.1 Overall Study Design 42

10.2 Special Procedures for Participants older than 55 years and younger than 18 years 45

10.3 Study Duration 46

10.4 Study Design and Dosage Rationale 46

10.5 Randomisation and Blinding 46

10.5.1 Selection of adult participants for blood sampling at visits B1, B2 and B3 47

10.5.2 Blinding Process 48

10.5.3 Unblinding 49

11. PARTICIPANT SELECTION, WITHDRAWAL AND DISCONTINUATION 50
11.1 Inclusion Criteria 50
11.2 Exclusion Criteria 50
11.3 In- and Exclusion criteria for Booster Vaccination (Adults and Adolescents) 52
11.4 Delay Criteria for Vaccination 52
11.5 Pregnancy Testing and Birth Control 52
11.6 Participant Withdrawal or Discontinuation 53
11.6.1 General 53
11.6.2 Access to a nationally deployed or approved COVID-19 vaccine 55
11.7 Lost to Follow-up 55
11.8 Re-screening of Participants 55
12. INVESTIGATIONAL MEDICINAL PRODUCT 56
12.1 Description of IMPs 56
12.1.1 VLA2001 56
12.1.2 AZD1222 56
12.1.3 Placebo 56
12.2 IMP Labelling & Packaging 57
12.3 IMP Storage 57
12.3.1 Dispensing and Accountability of Investigational Medicinal Product 57
13. STUDY PROCEDURES 58
13.1 Informed Consent and Enrolment 58
13.2 Participant Identification Code 58
13.3 Investigational Treatment 58
13.3.1 Description of Treatment 58
13.3.2 Vaccine Preparation and Administration 58
13.4 Post- Vaccination Observation 59
13.4.1 Adult (>55 years of age) Sentinel Group 59
13.4.2 Adolescent Sentinel Group 59
13.5 Monitoring of COVID-19 symptoms 59
13.6 Monitoring for blood clotting events following COVID-19 vaccination 62
13.7 Screening and Study Visits 62
13.7.1 Unscheduled Visit 63
13.7.2 Early Termination Visit 63
13.8 Procedures for Monitoring Participant Compliance 64
14. ASSESSMENT OF IMMUNOGENICITY 65
14.1 Additional Testing Procedures 65
15. ASSESSMENT OF SAFETY 66
15.1 Definitions 66
15.1.1 Adverse Events 66
15.1.2 Serious Adverse Event 66
15.1.3 Adverse Events of Special Interest 67
15.1.4 Medically-Attended Adverse Event 67
15.1.5 Pre-existing Diseases 67

15.1.6 Untoward Medical Occurrences Not Considered Adverse Events ... 67
15.2 Collection, Documentation and Assessment of Adverse Events ... 67
15.2.1 Unsolicited Adverse Events ... 67
15.2.2 Severity ... 68
15.2.3 Causality Assessments ... 68
15.2.4 Assessment and Outcome of Adverse Events ... 69
15.2.5 Solicited Adverse Events ... 69
15.2.6 Injection Site Reaction – Measurement and Evaluation ... 70
15.2.7 Systemic Reactions – Measurement and Evaluation ... 71
15.2.8 Fever and Body Temperature ... 71
15.3 Electronic Diary ... 71
15.4 Medical, Medication, and Non-Drug Therapy History ... 72
15.4.1 Medical history ... 72
15.4.2 Concomitant Medications and Non-Drug Therapies ... 72
15.5 Vital Signs ... 73
15.6 Physical Examinations ... 74
15.7 Clinical Laboratory Parameters ... 74
15.8 Safety Reporting Procedures and Oversight ... 75
15.8.1 Serious Adverse Events and Adverse Events of Special Interest ... 75
15.8.2 Pregnancy ... 76
15.8.3 Data Safety Monitoring Board ... 76
15.8.4 Sponsor ... 78
16. STATISTICAL ANALYSIS ... 79
16.1 Sample Size and Power Calculations ... 79
16.2 Analysis Populations: ... 79
Analysis of T-cell response will be done for those participants from whom PBMC samples are available (see section 10.5.1). ... 80
16.3 Handling of Missing, Unused, and Spurious Data ... 80
16.4 Methods of Analysis ... 80
16.4.1 Immunogenicity Analysis ... 81
16.4.2 Safety Analysis ... 81
16.5 Clinical Study Report ... 81
17. ETHICS AND REGULATORY ASPECTS ... 82
17.1 Compliance Statement ... 82
17.2 Ethics Committee and Regulatory Authorities ... 82
17.3 Participant Information and Informed Consent ... 82
18. QUALITY CONTROL AND QUALITY ASSURANCE ... 83
18.1 Source Data and Records ... 83
18.2 Investigator's Responsibility ... 83
18.3 Training ... 83
18.4 Monitoring ... 83
18.5 Audit and Inspection ... 83
18.6 Non-Compliance with the Protocol/Deviations from the Protocol ... 83

18.7 Confidentiality of Participant's Data 84
19. DATA HANDLING AND RECORD KEEPING 85
19.1 Information of Investigators 85
19.2 Electronic Case Report Forms 85
19.2.1 Data Recorded Directly on Case Report Forms 85
19.2.2 Electronic Case Report Form Entries 85
19.2.3 Changes to Electronic Case Report Form Data 85
19.2.4 Electronic Case Report Form Entry Validation 85
19.2.5 Data Collection 85
19.3 Coding of Adverse Events, Drugs and Diseases 86
19.4 Investigator File 86
19.4.1 Maintenance 86
19.4.2 Archiving and destruction 86
19.4.3 Provision of Additional Information 86
20. PUBLICATION POLICY 87
21. LIABILITIES AND INSURANCE 88
22. SUPPLEMENTS 89
22.1 Schedule of Study Procedures and Assessments (Adults) 90
22.1 Schedule of Study Procedures and Assessments (Adults – Booster Phase) 93
22.2 Schedule of Study Procedures and Assessments (Adolescents – Blinded part) 96
22.3 Schedule of Study Procedures and Assessments (Adolescents – Unblinded part) 99
22.4 Toxicity Grading Scale for Abnormal Laboratory Assessments 102
22.5 Adverse Events of Special Interest 105
23. REFERENCES 107

7. LIST OF ABBREVIATIONS

Abbreviation	Definition
AE	Adverse event
AESI	Adverse event of special interest
ALP	Alkaline phosphatase
ALT	Alanine aminotransferase
aPTT	Activated partial thromboplastin time
AST	Aspartate aminotransferase
BMI	Body mass index
CFR	Case fatality ratio
CI	Confidence interval
CpG	Cytosine phospho-guanine
CRA	Clinical research associate
CRO	Contract research organisation
CRP	C-reactive protein
CSR	Clinical study report
DSMB	Data Safety Monitoring Board
EC	Ethics committee
eCRF	Electronic case report form
eDiary	Electronic diary
ELISA	Enzyme-linked immunosorbent assay
EMA	European Medicines Agency
ET	Early termination (visit)
ESR	Erythrocyte sedimentation rate
FDA	Food and Drug Administration
GCP	Good Clinical Practice
GMFI	Geometric mean fold increase
GMT	Geometric mean titre
IB	Investigator’s Brochure
ICF	Informed consent form
ICH	International Conference on Harmonisation
IMP	Investigational medicinal product
ITT	Intent-to-treat (population)

Abbreviation	Definition
IRS	Interactive Response System
MedDRA	Medical Dictionary for Regulatory Activities
NHS	National Health Service
NHP	Non Human Primate
PP	Per-protocol population
PBMC	Peripheral Blood Mononuclear Cell
PPAS	Per-Protocol Analysis Set
PRNT	Plaque reduction neutralization test
RT-PCR	Reverse transcription polymerase chain reaction
SAE	Serious Adverse Event
SAP	Statistical Analysis Plan
SARS-CoV-2	Severe Acute Respiratory Syndrome Coronavirus-2
SOP	Standard operating procedure
SUSAR	Suspected Unexpected Serious Adverse Reaction
Th	T helper cell
UK	United Kingdom
ULN	Upper limit of normal
WBC	White blood cell

8. INTRODUCTION

8.1 Clinical Condition/Indication

8.1.1 Transmission, Disease and Diagnosis

Since December 2019, coronavirus-induced disease 2019 (COVID-19) has spread around the world, with over 24 million confirmed cases as of the 27 Aug 2020 (https://coronavirus.jhu.edu/, accessed 28 Aug 2020). SARS-CoV-2 was initially isolated, and the genome was published internationally by Chinese scientists on January 10, 2019. SARS-CoV-2 is the seventh member of the family of coronaviruses to infect humans. Novel coronaviruses from Wuhan, together with 2 bat-derived SARS-like strains, form a distinct clade in lineage B of the subgenus Sarbecovirus. SARS-CoV-2 is a group 2b coronavirus (as are MERS-CoV and SARS-CoV), with a whole genome similarity of up to 80% to SARS-CoV (Yang and Leibowitz, 2015). SARS-CoV-2 is an enveloped, non-segmented, positive sense RNA virus. Structurally, SARS-CoV-2 has 4 main structural proteins, which are conserved across coronaviruses; namely the spike glycoprotein, small envelope glycoprotein, membrane glycoprotein, and nucleocapsid protein, along with several accessory proteins. The spike protein facilitates binding of envelope viruses to host cells by attraction with angiotensin-converting enzyme 2 (ACE2) expressed in lower respiratory tract cells. (Astuti and Ysrafil, 2020).

Human infection is characterized by an incubation period of 2 to 7 days (typically 5 days across many studies), up to a maximum of 14 days. Symptoms of COVID-19 are non-specific, and the disease presentation can range from no symptoms (asymptomatic) to severe pneumonia and death. Common symptoms include fever, cough, dyspnoea, myalgia, fatigue, sputum, haemoptysis and diarrhoea. Lymphocytopenia and pneumonia are also common (Chaolin et al, 2020; Guan et al, 2020). In a study of 41 participants, complications included acute respiratory distress syndrome (29%), acute heart injury (12%), and secondary infections (10%); 32% of participants required treatment in the intensive care unit (Chaolin et al, 2020). In an analysis of 1099 confirmed cases, 25.2% of participants had at least one underlying disease (such as hypertension, chronic obstructive pulmonary disease). On admission, 50% of the participants presented ground-glass shadow on a chest computed tomography scan (Guan et al, 2020). Recent studies indicate that participants ≥ 60 years of age are at higher risk than children (Li et al, 2020a; Wang et al, 2020). Up to 5% of participants with COVID-19 developed acute respiratory distress syndrome (Guan et al, 2020), (Li et al, 2020a), (Guan et al, 2019). According to the World Health Organisation (WHO), 10% to 15% of participants ≤ 50 years old experience moderate to severe infection. Recovery time is about 2 weeks for persons with mild disease and 3 to 6 weeks for persons with severe or critical disease, based on Chinese Centres of Disease Control and Protection data and the current U.S. experience in California and Washington.

Analysis of the first 55,924 laboratory confirmed COVID-19 cases in China showed that mortality increases with age, with the highest mortality among people over 80 years of age (case fatality ratio [CFR] 21.9%). The CFR was higher among males than females (4.7% vs. 2.8%). The CFR was much higher in participants with comorbidities (CFR 7.6% to 13.2%) than those without (CFR 1.4%) (WHO, 2020). Similar results were found in a study analysing 17,425,445 adults in the UK between 01 February and 25 April 2020. In total 5683 deaths were attributed to COVID-19 (Williamson et al, 2020).

It is not known if SARS-CoV-2 will remain as worldwide pandemic. It is also not known how long the immunity that is acquired after symptomatic or asymptomatic SARS-CoV-2 infection can last.

The general strategies for clinical disease management include bed rest and supportive treatment, including antiviral therapy (Arabi et al, 2018), antibiotic application, immunomodulating therapy (Arabi et al, 2020), organ function support, respiratory support, bronchoalveolar lavage, blood purification and extracorporeal membrane oxygenation (ECMO; Wang et al, 2020; Zumla et al, 2020).

For detailed information on the disease and epidemiology please refer to the Investigator's Brochure.

8.1.2 Prospects for Vaccine Development

With the aim of preventing the spread of COVID-19 disease and to control the current pandemic, numerous SARS-CoV-2 vaccine candidates are being developed. These vaccine candidates are based on several different technology platforms and many of them express the spike protein (S-protein) or parts of the spike protein, i.e., the receptor binding domain (RBD), as the immunogenic determinant. Some of these have already received regulatory approval, others have reported efficacy data but are not yet approved; other candidates are still in earlier stages of development.
The first COVID-19 vaccines were designed and developed for rapid production and distribution; placebo-controlled efficacy studies have shown protection against moderate to severe disease, although the durability of this protection is currently unclear.
Even prior to vaccine rollout, it has been observed that the S-protein is a locus for rapid evolutionary and functional change as evidenced by the D614G, Y453F, 501Y.V2, and VUI-202012/01 mutations/deletions. This propensity for mutation of the S-protein leads to future risk of efficacy reduction over time as these mutations accrue. Taking into consideration that COVID-19 is expected to become endemic and that the virus mutations may adversely affect the efficacy of the current vaccines (including those still in development), there is a need for additional vaccines to complement the global vaccination programs and solutions.

As the first wave vaccines generate more and more information from clinical trials, there is data that suggests a close correlation between the immune response post vaccination and vaccine efficacy. Aiming for an increased efficiency of the pandemic response there is opportunity for the next-wave vaccines to adapt their clinical development plans and licensure pathway accordingly.

8.2 Valneva's Candidate Vaccine: VLA2001

VLA2001 is a highly purified, whole virus, SARS-CoV-2 vaccine produced on Vero cells and inactivated with β-propiolactone. The viral strain is derived from a Chinese tourist from Hubei diagnosed in a hospital in Rome (Stefanelli et al, 2020). VLA2001 will be adjuvanted with the licensed adjuvant cytosine phospho-guanine (CpG) 1018 (produced by Dynavax, contained in HEPLISAV-B®) in combination with aluminium hydroxide.

VLA2001 has been developed using the same manufacturing platform as that used for the commercial vaccine IXIARO®, a purified, inactivated, whole virus, aluminium hydroxide-adjuvanted Japanese encephalitis vaccine that has been approved by regulatory authorities worldwide (including the Food and Drug Administration [FDA], European Medicines Agency [EMA] and Therapeutic Goods Administration). It is similar in composition to VLA2001, with a good safety profile consistent with findings from pre-licensure clinical studies. As of April 2017, 2,532,480 participants worldwide have been vaccinated with IXIARO (IXIARO clinical review memorandum, 2018).

VLA2001 has demonstrated excellent purity and overall a biological, chemical and physical profile comparable to that of IXIARO®.

A detailed description of the vaccine and the mechanism of action can be found in the Investigator's Brochure.

8.3 Findings from Nonclinical and Clinical Studies

8.3.1 Non-clinical Summary

VLA2001 was immunogenic in mice and non-human primates. In mice, an increase in immunogenicity (dose sparing effect) was observed when CpG-1018 was used together with alum. The presence of CpG-1018 skewed the immune response towards a pronounced Th1 response. A strong Th1 response is important to minimize potential risks for enhanced

respiratory disease (ERD) or antibody disease enhancement (ADE) upon infection. One potential cause for ERD or ADE is a strong Th2 response. In addition, neutralising antibodies could be measured at the highest immunization dose (3.0 AU) in the presence of CpG-1018, the measured neutralising titre was in a similar range as the neutralising titre determined for the plasma pool derived from convalescent donors.

Cynomolgus macaques immunized with VLA2001 were protected from a combined intranasal and tracheal challenge with SARS-CoV-2. SARS-CoV-2 genomic RNA could transiently be detected in immunized macaques on day 2 post infection at a very low level in nasoparyngeal and tracheal swabs, but not at all in BAL. Subgenomic SARS-CoV-2 RNA was not detectable at all in nasopharyngeal and tracheal swabs, nor in BAL. No difference between VLA2001, high and medium dose could be detected in regard to the level of protection observed.

VLA2001 was tested in a repeat dose toxicology study in rats. The observations seen in the study are expected physiological and immunological response to a vaccine. Furthermore, some of the local reactions might be due to relatively large volume compared to the muscle mass in rats. Overall, VLA2001 was well tolerated by rats.

For further details please refer to the Investigator's Brochure.

8.3.2 Clinical Summary

Two additional clinical studies (VLA2001-201, VLA2001-304) are ongoing at this time. The study is a randomised, dose-escalation and dose-finding, multicentre study with three dose groups (low, medium and high).

The primary objective of this study is to evaluate the tolerability, safety and immunogenicity of the inactivated, adjuvanted SARS-CoV-2 vaccine candidate VLA2001 up to 14 days after completion of a two-dose schedule in healthy adults aged 18 to 55 years.

A total of 153 participants (51 per each dose group) have been recruited into the study

An interim analysis with data up to Day 106 is available. In this analysis, VLA2001 was found to be generally safe and well tolerated across all dose groups tested, with no safety concerns identified by an independent Data Safety Monitoring Board. There were no statistically significant differences between dose groups and no differences between first and second vaccinations in terms of reactogenicity. The majority of Adverse Events (AEs) were mild or moderate and only two subjects reported severe solicited AEs (headache and fatigue). The majority of solicited AEs resolved quickly. Only 19.6% of study participants reported unsolicited adverse events up to Day 106 which were considered related to the vaccine; no severe unsolicited AEs were reported. There were no serious related AEs. One adverse event of special interest (AESI) was observed (event term: chilblains). According to protocol an AESI was to be considered a medically important condition, consequently meeting reporting requirements as serious adverse events (seriousness criterion: medically important condition). No other SAE was reported. The investigator reported the event of chilblains as mild in severity and assessed the event as unrelated to the study vaccination. This AESI was observed in one participant in the medium dose group who experienced chilblains 4 days after 1st vaccination (start date 17 January 2021). The subject developed chilblains on toes, namely redness and swelling of toes. Due to this event, the subject underwent a COVID-19 PCR test at the site on 20 January 2021. The result of the PCR COVID-19 test was negative. No treatment was initiated by the investigator for this event. No action was taken with regards to the study vaccine for this event. Subject received second dose of the study vaccine.

VLA2001 was highly immunogenic with more than 90% of all study participants developing significant levels of antibodies to the SARS-CoV-2 virus spike protein across all dose groups tested. Seroconversion Rates (SCR) for S-protein binding IgG antibodies were 89.8% in the medium dose and 100% in the high dose group. Two weeks after completion of the two-dose schedule, Geometric Mean Fold Rises (GMFRs) from baseline were 26 in the medium dose and 86 in the high dose group. Of note, the IgG antibody response was highly correlated

with neutralization titres (MNA50) (r=0.79, p<0.001). VLA2001 induced a dose dependent response with statistically significant higher Geometric Mean Titres (GMTs) for both IgG and neutralising antibodies in the high dose group compared to the low and medium dose groups. In the high dose group which is the selected dose for this study, the GMT of neutralising antibodies antibody titres measured 2 weeks after completion of the 2-dose schedule was at or above levels for a panel of convalescent sera (GMT 530.4 (95% CI: 421.49, 667.52)). VLA2001 induced broad T-cell responses across participants with antigen-specific IFN-gamma producing T-cells against the S-protein, M and N protein detected in 75.6 %, 35.6% and 48.9% of study participants, respectively.

Another phase 3 clinical study (VLA2001-304) is currently ongoing in New Zealand. The study's objective is to generate additional immunogenicity and safety data in older participants (> 55 years). The study recruited 306 participants who received 2 doses of VLA2001 (33AU) at an interval of 28 days. First results are expected in Q1 2022.

More information on the clinical profile of VLA2001, can be found in the Investigator's Brochure.

8.4 Study Rationale

This Phase 3 clinical study represents the pivotal study to evaluate safety and immunogenicity of VLA2001. The study is designed to show superiority of VLA2001 in terms of inducing neutralising antibodies over another SARS-CoV-2 vaccine for which efficacy has been demonstrated. As it will be explained in section 8.4.2, this comparison is intended to allow to reasonably predict the vaccine efficacy of VLA2001 through immuno-bridging to the licensed comparator vaccine. In addition, the safety profile of VLA2001 will be established in 3,000 participants aged 18 years and older. After safety has been established in adults (i.e. 1 month follow-up after completion of the two dose primary immunization) and been reviewed by the Data Safety Monitoring Board of the study, adolescents will be included in this study to establish the safety and immunogenicity of VLA2001 in this age group as well.

8.4.1 The Comparator vaccine: AZD1222

AZD1222 is a recombinant replication-defective chimpanzee adenovirus expressing the SARS-CoV-2 S surface glycoprotein. Development of AZD1222, previously referred to as ChAdOx1 nCoV-19, was initiated by the University of Oxford with subsequent transfer of development activities to AstraZeneca.

In the current composition, AZD1222 vaccine contains two excipients: Sodium and Ethanol. The vaccine contains less than 1 mmol sodium (23 mg) per 0.5 ml dose, that is to say essentially "sodium-free". Similarly, the vaccine contains 2 mg of alcohol (ethanol) per 0.5 ml dose. It is claimed that the small amount of alcohol in the vaccine will not have any noticeable effects.

In December 2020, AZD1222 vaccine was approved for emergency use in the United Kingdom (UK), following positive results from an interim analysis of clinical trials of AZD1222 in the UK and Brazil,

In January 2021, AstraZeneca's COVID-19 vaccine was also granted a conditional marketing authorisation (CMA) in the European Union (EU) for active immunisation to prevent COVID-19 caused by SARS-CoV-2, in individuals 18 years of age and older. However, following latest MHRA guidance, the vaccine will not be offered to vaccinees below 30 years of age.

According to the approved European prescribing information and Regulation 174 Information for UK healthcare professionals, AZD222 vaccination course consists of two separate doses of 0.5 ml each. The second dose should be administered between 4 and 12 weeks (28 to 84 days) after the first dose.

8.4.2 Justification for the choice of comparator

AZD1222 has been tested in several clinical studies including studies evaluating efficacy. Between 23 April and 06 December 2020, 24,422 participants were recruited and vaccinated across four studies evaluating vaccine efficacy, of whom 17,178 were included in the primary analysis (8,597 receiving ChAdOx1 nCoV-19 and 8,581 receiving control vaccine). 332 confirmed SARS-CoV-2 infections met the primary endpoint of symptomatic infection more than 14 days after the second dose. Overall vaccine efficacy more than 14 days after the second dose was 66·7% (95% CI 57·4–74·0), with 84 (1·0%) cases in the 8597 participants in the ChAdOx1 nCoV-19 group and 248 (2·9%) in the 8,581 participants in the control group. To date AZD1222 is the only licensed SARS-CoV-2 vaccine for which different efficacy read-outs are available which have been associated with different levels of neutralization titres. Plotting anti-SARS-CoV-2 neutralising antibodies against vaccine efficacy for each dose interval showed evidence of a relationship between neutralising antibodies and vaccine efficacy (figure 1).

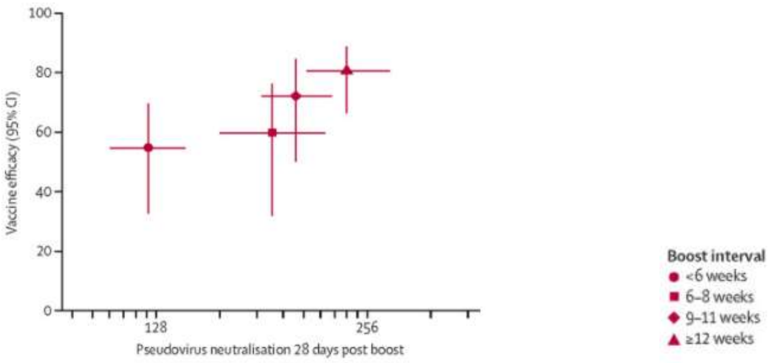

Figure 1. Correlation of neutralization titre and efficacy results with AZD1222 [adapted from Voysey et al. Lancet. 2021 Feb 19; doi: 10.1016/S0140-6736(21)00432-3].

Even with the immunization schedule of less than 6 weeks the reported efficacy was still higher than the WHO standard for COVID-19 vaccines of 50%. Consequently, comparison and demonstration of superiority in terms of neutralising antibodies against the schedule of 4 weeks of AZD1222 was shown to reasonably likely predict a vaccine efficacy above the WHO standard threshold of 50% efficacy for SARS-CoV-2 vaccines [Voysey et al. Lancet. 2021 Feb 19; doi: 10.1016/S0140-6736(21)00432-3].

Immuno-bridging of this association between AZD1222 to other vaccines like VLA2001 is considered possible because the induction of immune responses is ultimately at the centre of conferring protection and therefore the intermediary steps like mode of delivery and differences in antigen processing may be considered less relevant than the evaluation of the immune response induced by a vaccine candidate.

Supportive data of the universal importance of neutralising antibodies exist independently of vaccine platform, correlation of neutralising antibodies was suggested when comparing the neutralization and efficacy results of different vaccines that have successfully demonstrated efficacy:

The ratio of neutralising antibodies induced by 7 different vaccines (including AZD1222 as well as another whole virus inactivated vaccine) in their respective analyses of Phase 1/2 studies and human convalescent sera showed that 79.9% of variance in efficacy can be explained by neutralising antibodies. In other words, there is a strong correlation of ρ= 0.83 between neutralising antibody responses and efficacy regardless of vaccine technology.

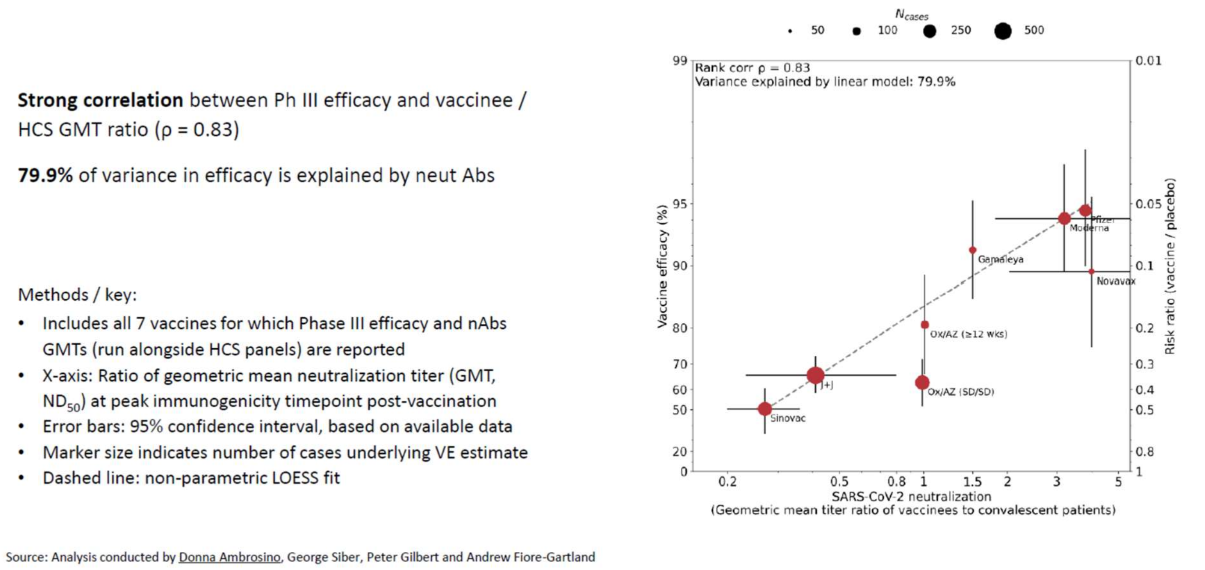

Figure 2. Phase III efficacy correlation of Phase I/II neutralization titre in relation to human convalescent sera presented at COVAX Workshop: February 25th

There is not a single example for which low neutralising antibody levels were associated with surprisingly high efficacy results or the other way around, high neutralising antibodies associated with low reported efficacy. On the contrary, the findings are very consistent across all vaccine platforms. Regardless, if mRNA, whole virus inactivated, vectored or subunit vaccine candidates were assessed, the highest neutralising antibodies are associated with the highest efficacy results for all vaccine candidates tested in phase 3 studies so far.

In conclusion, even though a specific correlate predictive of protection against COVID-19 for any vaccine candidate has not been established to date, evidence from laboratory studies, pre-clinical animal studies, and clinical studies suggest that neutralising antibody responses are reasonably predictive for vaccine efficacy for any SARS-CoV-2 vaccine candidate including a whole virus inactivated vaccine like VLA2001. Therefore, the benchmark of neutralising antibodies induced by AZD1222 when using a 4-week schedule is considered to reasonably likely predict vaccine efficacy in line with the WHO standard of above 50%.

8.4.3 Possible Benefits for the Participant

This is the second study with VLA2001 in human participants, therefore the clinical benefits of VLA2001 have not yet been established. As of 15 February 2021, 3 vaccines have been authorised in the UK and some more vaccines have been approved for use in several countries; this study includes an active comparator (AZD1222) that will be used according to an approved schedule, in line with the prescribing information.

Nevertheless, the overall benefit and risk balance for individual participants in the study cannot be ascertained. The participants will be informed that VLA2001 has not yet been proved to be safe and effective to prevent COVID-19.

Participants in this study may benefit from vaccination with the active comparator, participants in the VLA2001 arm may benefit from repeated physical examinations together with the laboratory assessments as prophylactic measures for an early diagnosis of any illness.

In addition, adolescent participants may benefit from access to a COVID-19 vaccine which has been shown to be safe in an adult population. At this point in time, this age group is not yet recommended to receive nationally deployed vaccines as part of the vaccine roll-out program in the UK. Adolescent participants who will be randomized to placebo will receive active immunization as well, albeit in a delayed fashion.

8.4.4 Possible Benefits for Society

SARS-CoV-2 has a high infection rate and has the potential to cause serious illness, especially in older populations and those people who are immunocompromised. A vaccine for SARS-CoV-2 will help reduce the severe and unprecedented disruption the pandemic has caused to people's lives worldwide. It will reduce the burden of healthcare services that had to find extra resources to care for critically ill people with COVID-19 and will also reduce the risk to frontline workers of contracting the virus. The pandemic has had a huge economic impact globally, forcing many businesses to close and people to isolate or shield indoors to prevent the spread of the virus. Many people have lost their jobs as a result. Businesses have been burdened by the extra cost of disinfectant, plastic shields, social distancing (fewer customers) and personal protective equipment. Additional effective vaccines will help ameliorate these global impacts.

8.4.5 Possible Risks/Inconveniences for the Participant

VLA2001

In general, risks associated with vaccination with an inactivated virus vaccine are considered low and several inactivated whole virus vaccines, such as Japanese encephalitis vaccine IXIARO, have been shown to have an excellent safety profile (IXIARO Clinical Review Memorandum). One study has been conducted with VLA2001. So far, 153 healthy volunteers aged 18 to 55 years have been given this vaccine candidate at different dose levels. Thereof, 51 were given the dose level of the vaccine tested in this study. Overall, the study showed that VLA2001 was safe and well-tolerated at all dose levels tested. A description of the most frequent side effects (observed in at least 1 out of 10 study participants) that were observed following vaccination and how common they were, is given below. Overall, about seven of 10 volunteers in the first study noted any side effects. Most Common side effects were vaccination site tenderness, Vaccination site pain, headache, Tiredness and muscle pain. The majority of events was considered mild and moderate and resolved within a few days. Only two study participants had reported severe side effects (one subject reported severe fatigue for a single day; one subject reported severe fatigue and headache for a single day). The type and frequency of side effects reported following the first and second vaccination were comparable.

AZD1222

The overall safety of COVID-19 Vaccine AstraZeneca (AZD1222) is based on an interim analysis of data from four clinical trials conducted in the United Kingdom, Brazil, and South Africa. At the time of analysis, 23,745 participants ≥18 years old had been randomized and received either AZD1222 or control. Out of these, 12,021 received at least one dose of AZD1222. Overall, among the participants who received AZD1222, approximately 9 of 10 participants were aged 18 to 64 years and approximately 1 of 10 was 65 years of age or older. Most Common side effects were vaccination site tenderness, Vaccination site pain, headache, fatigue, muscle pain, malaise, feverishness or fever ≥38°C and chills. The majority of side effects were mild to moderate in severity and usually resolved within a few days of vaccination. When compared with the first dose, side effects reported after the second dose were milder and reported less frequently. Side effects were generally milder and reported less frequently in older adults (≥65 years old).

Rare cases of severe and sometimes life-threatening thrombosis (blood clotting) inside blood vessels in different parts of the body including the brain, associated with low numbers of circulating platelets (small blood cells involved in the process of blood clotting) have been described in people during the period after they received AZ1222. As these events are very rare, it is very unlikely that any case would occur in this study. Nevertheless, participants will be informed about specific signs and symptoms which can be warnings signs of blood clotting events and will be advised to contact the study site immediately for further in-depth assessments.

Following latest MHRA guidance, vaccinees aged below 30 years will not receive AZD1222.
https://www.gov.uk/government/publications/use-of-the-astrazeneca-covid-19-vaccine-jcvi-statement

https://www.gov.uk/government/publications/regulatory-approval-of-covid-19-vaccine-astrazeneca/information-for-healthcare-professionals-on-covid-19-vaccine-astrazeneca

General

Since COVID-19 remains a risk now and in the coming months and may cause severe illness, including thrombosis and death, the balance of risk and benefit is considered to be strongly in favour of receiving either vaccine.

The study team will be available to respond to any concerns and ensure correct investigations and treatment are provided to study participants if they develop any symptoms of illness of any kind throughout the period of participation and follow up.

The likelihood of a participant to experience a vaccine-associated enhanced disease (VAED) following administration of VLA2001 is considered low but cannot be excluded.

Vaccine-induced enhancement of disease has also been described for SARS-CoV and MERS-CoV in animal models, but proof of human SARS-CoV or MERS-CoV vaccine associated enhanced disease does not exist as these candidate vaccines were never tested for efficacy nor used in outbreak situations. For SARS and MERS, the mechanism of enhanced disease observed in mice has been associated with a Th2-mediated eosinophilic infiltration in the lung, which is reminiscent of ERD effects observed after RSV infection of mice immunized with RSV. While vaccine-associated enhanced disease was observed in nonclinical studies with experimental SARS and MERS vaccines, it is not a given that the same risk applies to COVID-19 vaccines. To the Sponsor’s knowledge, antibody-related COVID-19 disease enhancement has not been observed in nonclinical models yet. In addition, vaccine mediated disease enhancement was not observed in Phase 1/2 studies of other whole virus inactivated SARS-CoV-2 vaccine candidates (Xia et al 2020).

Participants in the present study will be informed of the theoretical risk of disease enhancement in the informed consent form (ICF) or assent form and as a risk mitigation strategy, all enrolled participants will intensively monitored during the conduct of the study to rapidly diagnose COVID-19 and refer for treatment, if applicable.

The blood draws performed during the study carry the possible risks of pain, hematoma, and in very rare cases an infection at the venepuncture site.

After vaccination participants will remain at the study site for close observation by study team to monitor development of any acute reactions.

Necessary emergency equipment and medications must be available at the study site to treat severe allergic reactions.

9. STUDY PURPOSE AND OBJECTIVES

9.1 Study Purpose

The purpose of this study is to compare the immunogenicity of VLA2001 vaccine to AZD1222 vaccine in adults, and to establish the immunogenicity and safety of VLA2001 in adolescents.

9.2 Primary Objectives (Immunogenicity and Safety)

Immunogenicity:

1) To demonstrate the superiority of VLA2001 (Wuhan strain) compared to AZD1222 administered in a 2-dose immunization schedule 4 weeks apart, in terms of Geometric Mean Titre (GMT) ratio as well as non-inferiority in terms of seroconversion rate,of neutralising antibodies, at 2 weeks after the second vaccination (Day 43) in adults aged 30 years and older.
2) To examine the immunogenicity of VLA2001 administered in a 2-dose immunization schedule, 4 weeks apart, in terms of GMT and non-inferiority in terms of seroconversion rate, of neutralising antibodies, at 2 weeks after the second vaccination (Day 43) in adolescents aged ≥12 to <18 years compared to adults 18-29 years of age.

Safety:

3) To evaluate the safety and tolerability of VLA2001 at 2 weeks after the second vaccination (Day 43) in adults and adolescents aged 12 years and older.

9.3 Secondary Objectives (Immunogenicity and Safety)

Immunogenicity:

1) To assess immunogenicity of a 2-dose primary immunization schedule with VLA2001 in adults aged 18 years and above.
2) To assess immunogenicity of a 2-dose primary immunization schedule with VLA2001 in adolescents aged ≥12 to <18 years.
3) To determine the immunogenicity of a single booster dose with VLA2001 in adults and adolescents.
4) To evaluate cellular immune responses following administration of VLA2001 in adults and adolescents.

Safety:

5) To compare the safety of VLA2001 to AZD1222 up to Day 43 in adults aged 30 years and older, following a 2-dose immunization schedule 4 weeks apart.
6) To compare the safety of VLA2001 to placebo up to Day 71 in adolescents aged ≥12 to <18 years, following 2-dose primary immunization with VLA2001.
7) To describe the safety and tolerability of VLA2001 in adults up to 12 months after the first vaccination.
8) To describe the safety and tolerability of VLA2001 in adolescents aged ≥12 to <18 years following a 2-dose primary immunization schedule with VLA2001 up to 12 months after second vaccination.

9) To determine the safety and tolerability of a single booster dose with VLA2001 in adults and adolescents up to 6 months post booster dose.

9.4 Exploratory Objectives (Efficacy)

1) To assess the efficacy of VLA2001 in the prevention of COVID-19 in an adult population, aged 30 years and older.
2) To assess the efficacy of VLA2001 in the prevention of COVID-19 in an adolescent population, aged ≥12 to <18 years.

9.5 Study Endpoints

9.5.1 Primary Endpoints

Immunogenicity:

1) Immune response measured after completion of a 2-dose immunization schedule with VLA2001, as determined by the GMT ratio in adults and GMT in adolescents, as well as seroconversion (defined as 4-fold increase from baseline) of SARS-CoV-2-specific neutralising antibodies on Day 43.

Safety:

2) Frequency and severity of any Adverse Events (AE) up to Day 43 post-vaccination with VLA2001.

9.5.2 Secondary Endpoints

Immunogenicity:

1. Proportion of adult participants with seroconversion after receipt of 2 doses of study vaccination on Day 8 (age 55+ only), Day 29, Day 71, Day 208. Seroconversion is defined as ≥ 4-fold increase in SARS-CoV-2 neutralising antibody titre against the Wuhan strain and IgG antibodies directed against the S-protein of the Wuhan strain between Day 1 and the defined post-vaccination time points.
2. Proportion of adolescents, aged ≥12 to <18 years with seroconversion following a 2-dose primary immunization with VLA2001 at visits V3a and V4ab as well as V6p and V8p
3. Immune response in adults on Day 8 (age 55+ only), Day 29, Day 71, and Day 208, as determined by the GMT of SARS-CoV-2-specific neutralising antibodies.
4. Immune response in adolescents, aged ≥12 to <18 years, as determined by the GMT of SARS-CoV-2-specific neutralising antibodies following a 2-dose immunization schedule with VLA2001 at V3a and V4ab as well as V6p and V8p.
5. GMT ratio of SARS-CoV-2-specific neutralizing antibodies in the adolescent population vaccinated with VLA2001 at Day 43 (i.e. Visit 3a) and the adult population above 30 years (i.e. Visit 4).
6. Immune response in adults (age 55+ only) on Day 8 (age 55+ only), Day 29, Day 43, Day 71 and Day 208 , as determined by the GMT of IgG antibodies to SARS-CoV-2 S-protein.

7. Immune response in adolescents aged ≥12 to <18 years, as determined by the GMT of IgG antibodies to SARS-CoV-2 S-protein following a 2-dose immunization schedule with VLA2001 at V3a and V4ab as well as V6p and V8p.

8. GMT ratio of IgG antibodies to SARS-CoV-2 S-protein in the adolescent population vaccinated with VLA2001 at Day 43 (i.e. Visit 3a) and the adult population above 30 years (i.e. Visit 4).

9. Assessment of T-cell responses (Th1/Th2 polarization) from PBMCs on selected time points in a subset of participants after in vitro stimulation with SARS-CoV-2 antigens using e.g. ELISpot (IFNγ) or intracellular cytokine staining (IL-2, IL-4, IL-5, IL-13, TNFα, IFNγ)

Safety:

10. Frequency and severity of solicited injection site and systemic reactions within 7 days after each and after any vaccination (primary vaccination series).

11. Frequency and severity of any AE during the entire study period.

12. Frequency and severity of any unsolicited AE following the completion of the 2-dose immunization schedule with VLA2001.

13. Frequency and severity of any unsolicited vaccine-related AE following the completion of the 2-dose immunization schedule with VLA2001.

14. Frequency and severity of any serious adverse event (SAE) during the entire study period.

15. Frequency and severity of any adverse event of special interest (AESI) during the entire study period.

Additional immunogenicity endpoints for the subset of adult participants who received a single Booster dose of VLA2001:

B13) Geometric mean fold rise (GMFR) from pre-booster time point (day of booster vaccination, Visit B1) to 14 days (Visit B2) after booster dose with regards to SARS-CoV-2-specific neutralizing antibodies.

B14) GMT of SARS-CoV-2-specific neutralizing antibodies as measured by MNA50, at Visits B1, B2 and B3 including formal non-inferiority testing on the GMT ratio Visit 4/ Visit B2 for the booster subgroup who had received 2 doses of VLA2001 for primary immunization schedule.

B15) Proportion of participants with 4-fold increase from pre-booster time point (day of booster vaccination, Visit B1) to 14 days (Visit B2) after booster dose with regards to SARS-CoV-2-specific neutralizing antibodies.

B16) GMFR from pre-booster time point (day of booster vaccination, Visit B1) to 14 days (Visit B2) after booster dose with regards to S-protein binding antibodies.

B17) Proportion of participants with 4-fold increase from pre-booster time point (day of booster vaccination, Visit B1) to 14 days (Visit B2) after booster dose with regards to S-protein binding antibodies.

B18) GMT measured as IgG antibodies against SARS-CoV-2 as determined by ELISA, at Visits B1, B2 and B3.

B19) Assessment of T-cell responses from PBMCs in a subset of participants after in vitro stimulation with SARS-CoV-2 antigens using ELISpot (IFNγ) at Visits B1, B2 and B3.

Additional Safety endpoints for adult booster dose group:

B20) Frequency and severity of solicited injection site and systemic reactions within 7 days after booster vaccination in a subset of participants.

B21) Frequency and severity of any unsolicited AE up to 6 months after booster dose.

B22) Frequency and severity of any vaccine-related up to 6 months after booster dose.

B23) Frequency and severity of any serious adverse event (SAE) up to 6 months after booster dose.

B24) Frequency and severity of any adverse event of special interest (AESI) up to 6 months after booster dose.

Additional immunogenicity endpoints for the subset of adolescent participants who received a single Booster dose of VLA2001:

B13) Geometric mean fold rise (GMFR) from pre-booster time point (day of booster vaccination, V5ab) to 14 days after booster dose (V6ab) with regards to SARS-CoV-2-specific neutralizing antibodies.

B14) GMT of SARS-CoV-2-specific neutralizing antibodies as measured by MNA50, at Visits V5ab, V6ab and V7ab

B15) Proportion of participants with 4-fold increase from pre-booster time point (day of booster vaccination, V5ab) to 14 days after booster dose (V6ab) with regards to SARS-CoV-2-specific neutralizing antibodies.

B16) GMFR from pre-booster time point (day of booster vaccination, V5ab) to 14 days after booster dose (V6ab) with regards to S-protein binding antibodies.

B17) Proportion of participants with 4-fold increase from pre-booster time point (day of booster vaccination, V5ab) to 14 days after booster dose (V6ab) with regards to S-protein binding antibodies.

B18) GMT measured as IgG antibodies against SARS-CoV-2 as determined by ELISA, at Visits V5ab, V6ab and V7ab

B19) Assessment of T-cell responses from PBMCs in a subset of participants after in vitro stimulation with SARS-CoV-2 antigens using ELISpot (IFNγ) at Visits V5ab, V6ab and V7ab.

Additional Safety endpoints for adolescent booster dose group:

B20) Frequency and severity of solicited injection site and systemic reactions within 7 days after booster vaccination

B21) Frequency and severity of any unsolicited AE up to 6 months after booster dose.

B22) Frequency and severity of any vaccine-related up to 6 months after booster dose.

B23) Frequency and severity of any serious adverse event (SAE) up to 6 months after booster dose.

B24) Frequency and severity of any adverse event of special interest (AESI) up to 6 months after booster dose.

9.5.3 Exploratory endpoint

Occurrence of PCR confirmed SARS-CoV-2 infection

10. STUDY DESIGN

10.1 Overall Study Design

This is a multicentre, randomized, observer-blind, active-controlled, superiority, study in adults to compare the immunogenicity of VLA2001 to AZD1222 in terms of GMT. Furthermore, VLA2001 will be compared to placebo in an adolescent population.

Adult Population

In the adult population, approximately 4,000 adults will be recruited into the study. Participants will be adults aged ≥18 years older who are either generally healthy or are with a stable medical condition. Immunogenicity and safety of VLA2001 will be assessed up to Month 12 after the first vaccination.

Approximately 3,000 participants ≥30 years of age and older will be randomized overall in a 2:1 ratio to receive 2 intramuscular doses of either VLA2001 (n=2,000) or AZD1222 (n=1,000) at the recommended dose level, 28 days apart, on Days 1 and 29.

Figure 1: Study Overview - Adults

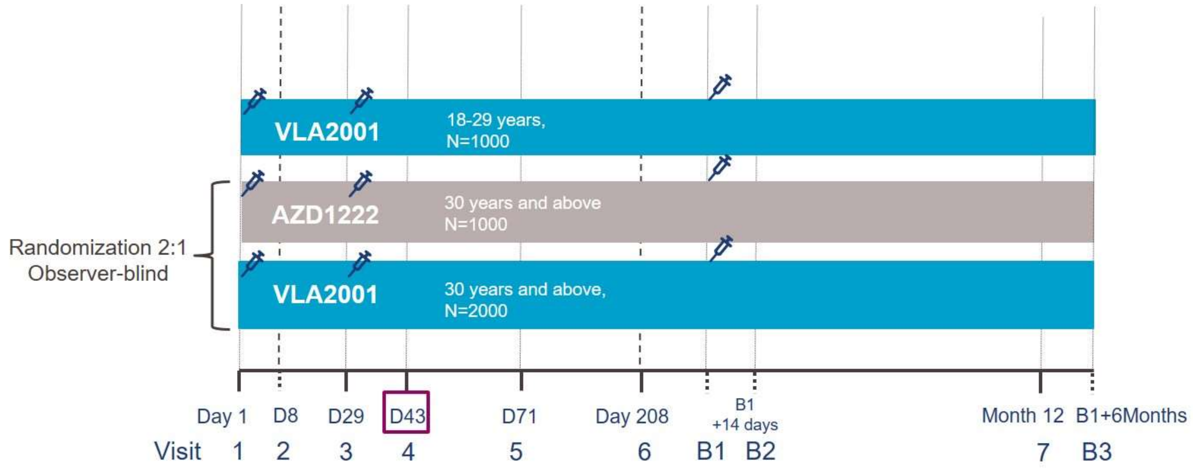

Note: For adults Day 8 visit is only applicable for participants of the immunogenicity subset.

For immunogenicity analyses, samples from approximately 1,200 adult participants (600 per group) who have been tested sero-negative for SARS-CoV-2 at screening will be analysed.

Approximately 1,000 participants that are under 30 years of age will be placed in a non-randomized treatment group and receive VLA2001 at the recommended dose level, 28 days apart, on Days 1 and 29.

The first 10 participants who are older than 55 years at the time of signing the ICF, will be treated as a ‘sentinel’ group and will be observed for at least 60 minutes after each vaccination. Thereafter, participants will be observed for immediate AEs and/or reactogenicity for at least 30 minutes after the administration of the vaccine.

Participants will be provided with an electronic Diary (e-Diary) and will be trained to record specifically solicited systemic and local symptoms daily, as well as any additional AEs during the follow-up period after each of both vaccinations up to the next visit to the site, i.e. Day 43 visit has been completed.

The following information will be collected:

- Oral body temperature,
- Solicited local (i.e., injection site) reactions (predefined terms),
- Solicited systemic reactions (predefined terms),
- Other AEs, i.e. unsolicited events

Any new medications or changes in medication administered after vaccination

VLA2001 Booster Group in Adults

All participants – except those who already received a licensed COVID-19 vaccine outside of the study - will be offered a booster dose with VLA2001. All eligible and willing participants will receive a booster vaccination with VLA2001at Visit B1 and will have a follow-up visit 14 days (Visit B2) after the booster dose. The participants will have 1 more follow-up visit 6 months after the booster vaccination (i.e., Visit B3 which replaces Visit 7(Month 12) for those participants who received a booster dose). All participants who do not receive a booster dose with VLA2001 will continue with Visit 7 (Month 12).

Blood samples for immunogenicity analysis will be taken in a subset of adults who had received the primary vaccination with 2 doses of VLA2001 (N=330) as well as a subset of participants who had received 2 doses of AZD1222 for primary immunization (N=110). The timing of the booster dose administration will be scheduled after Visit 6 (Day 208) approximately between January and February 2022.

All visits will be conducted at the clinical site on an outpatient basis.

Participants will be provided with an electronic Diary (e-Diary) and will be trained to record specifically solicited systemic and local symptoms daily as well as any additional AEs during follow-up period after the booster vaccination up to the next visit to the site has been completed.

The following information will be collected:

- Oral body temperature,
- Solicited local (i.e., injection site) reactions (predefined terms),
- Solicited systemic reactions (predefined terms),
- Other AEs, i.e. unsolicited events
- Any new medications or changes in medication administered after vaccination

Adolescent Population

For safety reasons, the first 16 adolescents will be enrolled in an open label, non-randomized manner. The sentinel dosing is aimed to be done at a single site to ensure permanent oversight on safety data by one principal investigator during the recruitment of the 16 sentinel participants.

If, for logistical reasons, a second site needs to be involved in the recruitment of the sentinel participants, vaccinations will be limited to one site on a specific day. Safety data exchange between the study sites will be ensured.

The first 4 adolescents will be vaccinated 1 hour apart each. After vaccination, the participant will be observed for the development of any acute reaction at the study site for 3 hours after the vaccination procedure. Prior to discharge from the study site, vital signs will be measured and the participant/parents will be instructed in the use of the eDiary. The study site will contact the participant by phone approximately 24 and 48 hours after vaccination for safety follow-up. The provided information provided must be compared with the entries in the participant´s eDiary.

The minimum time before the next 4 participants are vaccinated is 24 hours after all participants of the previous sentinel cohort have received their vaccination. The same procedure is applied for the following 3 sentinel groups.

A Data Safety and Monitoring Board (DSMB) will review the accrued safety data when all 16 sentinel participants have completed the 7-day e-diary period after vaccination. After favourable DSMB review, randomization of the remaining participants across all sites will be initiated. Ad-hoc DSMB reviews will be performed if individual withdrawal criteria are met (section 11.6.1).

Subsequently, at least 660 participants will be recruited and randomized in a 1:1 ratio to receive 2 intramuscular doses of either VLA2001 (n=330) or placebo (n=330). Adolescents randomized to receive VLA2001, will receive a booster vaccination with VLA2001 at Visit V5ab (Day 208). Participants in the placebo-group will receive a 2-dose primary immunization (28 days apart) with VLA2001 at Visit V4p and Visit V5p (figure 2).

Figure 2 - Study Overview - Adolescents

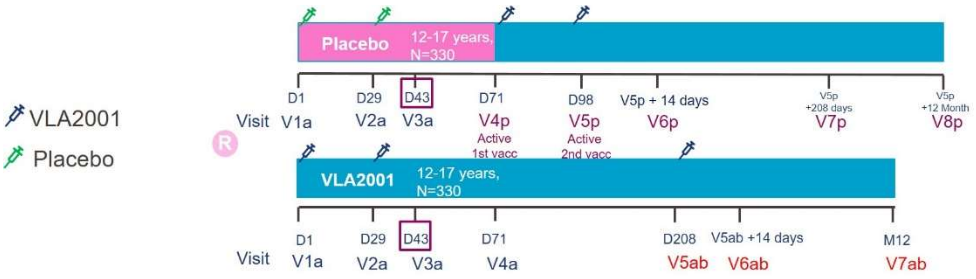

Due to the fact that the placebo group will receive a dose of VLA2001 at Visit V4p, all adolescent participants will be unblinded at this point in time. Visit V5p, V6p, V7p and V8p will only be performed by the participants initially randomized to the placebo-group.

Participants will be provided with an electronic Diary (e-Diary) and will be trained to record specifically solicited systemic and local symptoms daily, as well as any additional AEs during the follow-up period after each of both vaccinations up to the next visit to the site. Adolescents in the VLA2001-group will be asked to complete their e-Diary until Visit V6ab (i.e., the next site visit following VLA2001- booster vaccination), and adolescents in the placebo-group until Visit V6p (i.e., the next site visit following the second vaccination with VLA2001).

The following information will be collected:

- Oral body temperature,
- Solicited local (i.e., injection site) reactions (predefined terms),
- Solicited systemic reactions (predefined terms),
- Other AEs, i.e. unsolicited events
- Any new medications or changes in medication administered after vaccination

VLA2001 Booster Group in Adolescents

Adolescents initially randomized to receive the primary vaccination schedule with VLA2001 will receive a booster vaccination with VLA2001 at Visit V5ab and will have a follow-up visit 14 days after the booster dose (i.e., Visit V6ab). The participants will have 1 more follow-up visit 5 months after the booster vaccination (i.e., Visit V7ab).

All visits will be conducted at the clinical site on an outpatient basis.

Participants will be provided with an electronic Diary (e-Diary) and will be trained to record specifically solicited systemic and local symptoms daily as well as any additional AEs during follow-up period after the booster vaccination up to the next visit to the site has been completed.

The following information will be collected:

- Oral body temperature,
- Solicited local (i.e., injection site) reactions (predefined terms),
- Solicited systemic reactions (predefined terms),
- Other AEs, i.e. unsolicited events
- Any new medications or changes in medication administered after vaccination

10.2 Special Procedures for Participants older than 55 years and younger than 18 years

The first 10 participants who are older than 55 years at the time of signing the ICF, will be treated as a 'sentinel' group and will be observed for at least 60 minutes after each vaccination. In addition, there will be an early safety review of the 'sentinel' group by the DSMB (in parallel while recruitment continues).

The first DSMB safety review of this group will include all accrued safety data after first vaccination plus at least 1-week follow-up.

A second DSMB safety review by the DSMB for participants above 55 years of age will be conducted when first 10 participants above 55 years of age have received their second vaccination and have at least 1 week of safety follow-up.

For safety reasons, the first 16 adolescents will be enrolled in an open-label, non-randomized manner. The sentinel dosing is aimed to be done at a single site to ensure permanent oversight on safety data by one principal investigator during the recruitment of the 16 sentinel participants.

If, for logistical reasons, a second site needs to be involved in the recruitment of the sentinel participants, vaccinations will be limited to one site on a specific day. Safety data exchange between the study sites will be ensured.

The first 4 adolescents will be vaccinated 1 hour apart each. After vaccination the participant will be observed for the development of any acute reaction at the study site for 3 hours after the vaccination procedure. Prior to discharge from the study site, vital signs will be measured and the participant/parents will be instructed in the use of the eDiary. The study site will contact the participant by phone approximately 24 and 48 hours after vaccination for safety follow-up. The provided information must be compared with the entries in the participant´s eDiary.

The minimum time before the next 4 participants are vaccinated is 24 hours after all participants of the previous sentinel cohort have received their vaccination. The same procedure is applied for the following 3 sentinel groups.

A Data Safety and Monitoring Board (DSMB) will review the accrued safety data when all 16 sentinel participants have completed the 7- day e-diary period after vaccination. After favourable DSMB review randomization of the remaining participants across all sites will be initiated.

10.3 Study Duration

The overall study duration (First Participant First Visit [screened] – Last Participant Last Visit [LSO]) is estimated to be approximately 21 months.

The total study duration for individual participant (adults and adolescents) is up to 14 months from the day of signing the informed consent to study completion, unless prematurely discontinued.

10.4 Study Design and Dosage Rationale

This Phase 3 study is designed as a randomized, observer-blind, active-controlled, stratified study, in order to demonstrate the superiority of the immunogenicity of VLA2001 vaccine when compared to an already registered vaccine AZD1222, against COVID-19.
In order to compare VLA2001 with AZD1222, the vaccination administration scheme of VAL2001 has been adjusted to match that of AZD1222 so that the interval between the first dose and second dose is 28 days instead of 21 days as was done in the phase 1/2 study of VLA2001-201. Available data from other inactivated vaccines against SARS-CoV-2 suggest that extending the schedule may lead to similar if not better immune response. For example, results for the whole virus inactivated alum-only adjuvanted SARS-CoV-2 vaccine candidate from Sinopharm, in both clinical phases 1 and 2 indicated that a longer interval (21 days and 28 days) between the first and second injections produced similar antibody responses and significantly greater than a Day 0 & 14 schedule.

The timing of the primary endpoint analysis at Day 43 (i.e. two weeks after completion of the vaccination schedule) has been selected based on previous results for AZD1222 for which this time point represented the peak immune response (Ramasamy et al. LANCET 2020, https://doi.org/10.1016/S0140-6736(20)32466-1).

For the study population aged 30 years and above the study will be observer blinded to the Investigators and study team. However, the pharmacists/ nurses who prepare the vaccination will not be blinded since the packaging of the 2 vaccines is different. Observers (i.e. investigators and study team performing any study related assessments) remain blinded to minimize possibility of a safety bias by the participants and/or Investigator’s or staff performing medical safety assessments. Study participants below 30 years of age will be involved in an open-label manner.

The dose of 33 Antigen Units (AU) for VLA was selected based on the results of the Phase 1/2 study VLA2001-201 of 150 participants aged 18 to 55 years.

The dose of the comparator AZD1222 is the recommended registered dose as approved in the U.K.

Enrolment of adolescent participants will only be started when the enrolment of adults has been completed and safety information (i.e. 1 month follow-up after completion of the two dose primary immunization) for the adult participants who received VLA2001 has been reviewed by an independent DSMB.

Similar to other COVID-19 vaccines, adolescents ≥12 to <18 years of age will receive the full adult dose. The placebo group represents a “delayed vaccination” group and will contribute to both safety comparison to placebo as well as generation of additional safety data with VLA2001 since all participants – including the initial placebo recipients - will eventually receive a complete immunization with 2 doses of VLA2001.

10.5 Randomisation and Blinding

Approximately 3,000 participants 30 years of age and older will be randomized overall in a 2:1 ratio to receive 2 intramuscular doses of either VLA2001 (n=2,000) or AZD1222

(n=1,000) at the recommended dose level, 28 days apart, on Days 1 and 29.

At least 660 adolescent participants ≥12 to <18 years of age and older will be randomized overall in a 1:1 ratio to initially receive 2 intramuscular doses of either VLA2001 (n=330) or Placebo (n=330) at the same dose level as adults, 28 days apart.

For immunogenicity analyses, samples from approximately 1,200 adult participants (600 per group) and all adolescent participants (at least N=660, 330 per group) who have been tested sero-negative for SARS-CoV-2 at screening will be analysed.

Approximately 1,000 participants between 18-29 years of age will be placed in a non-randomized treatment group and receive VLA2001 at the recommended dose level.

The first two doses of vaccination will be administered 28 days apart, on Days 1 and 29.

The following conditions apply to the randomization:

- The immunogenicity subset in adults will be randomly assigned in a 1:1 ratio to receive either VLA2001 or AZD1222.
- 200 adult participants (100 adult participants of the VLA2001 immunogenicity subset and 100 adult participants of the AZD1222 immunogenicity subset) will be selected for PBMC sample collection and analysis.
- 100 adolescent participants (50 adolescent participants of the VLA2001 and 50 adolescent participants of the placebo group) will be selected for PBMC sample collection and analysis.

Each participant will have a unique participant screening number obtained from the Interactive Response System (IRS). The Investigator will keep a record (i.e. the participant screening log) of participants who entered screening.

The IMP will be prepared by unblinded study staff in accordance with the treatment allocation provided in the IRS.

More details about randomization, blinding and administration of the vaccines will be provided in the IRS manual, eCRF completion guidelines and the pharmacy manuals respectively.

10.5.1 Selection of adult participants for blood sampling at visits B1, B2 and B3

As described in section 10.1 all adult participants – except those who received a licensed COVID-19 vaccine outside of the study - will be offered a booster dose with VLA2001. Those participants who are not eligible or do not wish to receive a booster with VLA2001 will continue to proceed with Visit 7 (Month 12) as the last visit in the study.

The following process for selection of participants for the booster immunogenicity analysis in adults will be followed (only for these participants, will blood samples be collected at Visit B1, B2 and B3):

Study sites will schedule booster visits for the eligible and willing participants in a first come first serve fashion starting with participants who were in the Immunogenicity population and did not have a positive SARS-CoV-2 PCR result during the study. It is planned to collect blood samples of 280 participants aged 30 years and above and at least 50 participants aged 18-29 years of age vaccinated with VLA2001 as well as 110 participants from the AZD1222 group. As soon as the planned number of participants has been reached, blood sampling will stop, no blood samples will be taken from all remaining eligible and willing participants who will be scheduled to receive the booster vaccination.

Blood samples will be preferentially taken from participants who already provided samples for the previous immunogenicity analysis and PBMC analysis. Blood samples for PBMC analysis will be only be drawn at Visit B1, B2 and B3 from those participants receiving a booster and for whom PBMC blood samples have already been collected at previous visits.

In case the total subject numbers targeted for subjects vaccinated with VLA2001 (280/50) cannot be reached during selection of the booster immunogenicity population, subjects from the younger age group may be chosen to achieve a total of 330 VLA2001 vaccinated subjects in the booster immunogenicity population.

Note: Adolescent participants will be already randomized at the time of enrolment. Only those randomized into the active VLA2001 arm from the start will receive a booster vaccination (3rd dose with VLA2001). Adolescents randomized in the placebo arm will eventually receive 2 doses of VLA2001 but will not receive a booster vaccination.

10.5.2 Blinding Process

VLA2001 and AZD1222 will be provided to the study sites in multi-dose vials, placebo will be provided in a single-dose vial. All vials will have a unique kit number. In addition, the site will receive unlabelled syringes together with aspiration and administration needles and specific syringe labels. Once the unblinded pharmacist identifies the treatment for a participant via randomization in EDC, they will retrieve 0.5 mL from the respective multi-dose or single dose vial with a syringe. Immediately afterwards this syringe will be labelled with a syringe label and participant identifiers will be written manually on the label. With this process the identification of and allocation of the syringe to a specific vial and participant is ensured.

An overview of persons who will be blinded or un-blinded is provided below:

Unblinded:

- Study participants aged 18-29 years at the time of enrolment
- Adolescence participants after Visit 4p/4ab
- Members of the DSMB
- Unblinded pharmacist/ nurse who will prepare the vaccination dose
- Unblinded clinical research associates (CRAs) who are responsible for monitoring the tasks performed by the unblinded study team and for checking the vaccine inventory at the site.
- Unblinded statistician(s) at CRO who will be responsible for following tasks:
    - Generation of the randomization code list
    - Attendance of unblinded sessions of DSMB meetings
    - Generating safety data tables/listings/figures for the DSMB meetings

Blinded:

- Study participants aged ≥12 years to <18 years and 30 years and above at the time of enrolment
- All laboratory operators at central laboratories for immunogenicity laboratory assessments.
- Laboratory personnel at the Sponsor’s labs for additional testing procedures

For study participants aged ≥12 years to <18 years and 30 years and above:

- Investigators and other site staff, excluding the unblinded study team preparing the vaccine and managing inventory.

- Biostatisticians writing the Statistical Analysis Plan and developing the statistical analysis.
- CRAs responsible for monitoring study data, excluding those who may observe the study drug application process and will monitor the vaccine inventory.
- All other Sponsor and contract research organisation (CRO) staff including medical monitor; unblinding of these teams will occur during the Primary Analysis of Day 43 data – this applied to the adult participants.
- All other Sponsor and contract research organisation (CRO) staff including medical monitor and adolescent participants; unblinding of these teams will occur at Visit 4 (ie V4p or V4a) – this applied to the adolescent participants

Further details are presented in the IMP manual.

10.5.3 Unblinding

With the exception of section 11.5.1 the randomisation assignment is not to be revealed except in emergency cases where unblinding is necessary for the clinical management of an AE/SAE. In such events, the Investigator must either inform the Sponsor before breaking the blind or immediately after unblinding.

In case of emergency, the vaccine administered to the participants can be revealed through the IRS.

11. PARTICIPANT SELECTION, WITHDRAWAL AND DISCONTINUATION

Approximately 4,000 male or female adults and approximately 660 male or female adolescents who satisfy the inclusion and exclusion criteria listed below will be allowed to participate in the study.

11.1 Inclusion Criteria

Participants must meet **all** inclusion criteria to be eligible for the study.

1. All participants must have read, understood, and signed the informed consent form (ICF).
    a. For participants aged 16 years or older: Written informed consent prior to any study related procedures;
    b. For participants aged ≥12 to <16 years: Written informed consent by the participant's legal representative(s), according to local requirements, and written informed assent of the participant, if applicable, prior to any study related procedures;
2. Participants of either gender aged 12 years and older at screening.
3. Medically stable such that, according to the judgment of the investigator, hospitalization within the study period is not anticipated and the participant appears likely to be able to remain on study through the end of protocol-specified follow-up.
    - A stable medical condition is defined as disease not requiring significant change in therapy or hospitalization for worsening disease during the 3 months prior to the expected day of randomization (visit 1).
4. Must be able to attend all visits of the study and comply with all study procedures, including daily completion of the e-diary for 7 days after each vaccination.
5. Women of childbearing potential (WOCBP), who are sexually active with a man, must be able and willing to use at least 1 highly effective method of contraception (i.e. include hysterectomy, bilateral salpingectomy, and bilateral oophorectomy, hormonal oral medication, male condoms with spermicide, transdermal, implant, or injection, barrier [i.e. condom, diaphragm with spermicide]; intrauterine device; vasectomized partner [6 months minimum], clinically sterile partner; or abstinence) for a minimum of 3 months after the last dose of study vaccine of the primary vaccination series.
    - A female participant is considered to be a WOCBP after menarche and until she is in a postmenopausal state for 12 consecutive months (without an alternative medical cause) or otherwise permanently sterile.

    Note: Participants not of childbearing potential are not required to use any other forms of contraception during the study. Non-childbearing potential is defined as participant confirmed:

    - Surgical sterilization (e.g., bilateral oophorectomy, bilateral salpingectomy, bilateral occlusion by cautery, hysterectomy, or tubal ligation).
    - Postmenopausal (defined as permanent cessation of menstruation for at least 12 consecutive months prior to screening).
6. WOCBPs must have a negative pregnancy test prior to each vaccination.

11.2 Exclusion Criteria

Participants will not be eligible for the study if they meet any of the exclusion criteria or will be discontinued from study vaccination if they develop any of the following exclusion criteria during the study.

1. Participant is pregnant or planning to become pregnant within 3 months after study

vaccine administration.

2. History of allergy to any component of the vaccine.
3. Significant infection (e.g. positive SARS-CoV-2 RT-PCR) or other acute illness, including fever >100 °F (>37.8 °C) 48 hours before vaccination.
4. Participant has a known or suspected defect of the immune system, such as participants with congenital or acquired immunodeficiency, including infection with HIV, status post organ transplantation or immuno-suppressive therapy within 4 weeks prior to the expected day of randomization (Visit 1).
    - Immunosuppressive therapy is defined as administration of chronic (longer than 14 days) prednisone or equivalent ≥ 0.05 mg/kg/day within 4 weeks prior to the expected day of randomization (visit 1), radiation therapy or immunosuppressive cytotoxic drugs/ monoclonal antibodies in the previous 3 years; topical and inhaled steroids are allowed.
    - Participants with chronic HIV unless: HIV disease with documented viral load <50 copies/ml and CD4 count >200 cells/mm3 for at least 6 months before enrolment, and stable antiretroviral therapy for the last 6 months
5. Participant has a history of cerebral venous sinus thrombosis, heparin-induced thrombocytopenia or antiphospholipid syndrome.
6. Participant has a history of malignancy in the past 5 years other than squamous cell or basal cell skin cancer. If there has been surgical excision or treatment more than 5 years ago that is considered to have achieved a cure, the participant may be enrolled. A history of hematologic malignancy is a permanent exclusion. Participants with a history of skin cancer must not be vaccinated at the previous tumour site.
7. History of drug dependency or current use of drug of abuse or alcohol abuse at screening.
8. Significant blood loss (>450 mL) or has donated 1 or more units of blood or plasma within 6 weeks prior to the expected day of randomization (Visit 1).
9. History of clinically significant bleeding disorder (e.g., factor deficiency, coagulopathy, or platelet disorder), or prior history of significant bleeding or bruising following IM injections or venepuncture.
10. Severe and uncontrolled ongoing autoimmune or inflammatory disease history of Guillain-Barre syndrome or any other demyelinating condition.
11. Any other significant disease, disorder or finding which in the opinion of the investigator may significantly increase the risk to the volunteer because of participation in the study, affect the ability of the volunteer to participate in the study or impair interpretation of the study.

    Prior/concomitant therapy:

12. Receipt of immunoglobulin or another blood product within the 3 months before expected day of randomization (Visit 1) in this study or those who expect to receive immunoglobulin or another blood product during this study.
13. Receipt of medications and or vaccinations (other than the study vaccinations) intended to prevent COVID-19.
14. Receipt of any vaccine (licensed or investigational), other than licensed influenza vaccine, within 28 days prior to the expected day of randomization (Visit 1).

    Others:

15. Any member of the study team or sponsor.
16. An immediate family member or household member of the study's personnel.

11.3 In- and Exclusion criteria for Booster Vaccination (Adults and Adolescents)

In addition to the above described eligibility criteria, the following criteria must be met:

1. Participant has not received another licensed COVID-19 vaccine during the study

11.4 Delay Criteria for Vaccination

Vaccination will be delayed if:

4. Participant has an acute infection within 48 hours prior to vaccination or oral temperature ≥ 38.0 °C on the day of vaccination;
5. Participant has received antipyretics within 4 hours prior to the scheduled time of vaccination.
6. Participant has received any other vaccine (other than licensed influenza vaccine or for medical emergencies such as tetanus or rabies exposure), within 28 days.

The rescheduled visit should be within the specified time window for the vaccination visit. In case of a delay of the second vaccination beyond the pre-specified time window this must be captured as protocol deviation.

In any case, all efforts should be made to schedule the next vaccination as soon as possible after the participant is eligible for receiving it.

In addition, for a rescheduled vaccination all inclusion and none of the exclusion criteria must be met; in case not all of these criteria are met, the vaccination will be omitted.

11.5 Pregnancy Testing and Birth Control

Women of childbearing potential presenting with a negative pregnancy test and practicing the use of adequate birth control before conduct and during the first 3 months of the study are eligible for inclusion into the study.

A woman is considered of childbearing potential if fertile, following menarche and until becoming post-menopausal unless permanently sterile.

Women of childbearing potential must have practiced an adequate contraceptive method during the 30 days before Visit 0 (Screening Visit).

A **urine** pregnancy test will be done prior to vaccination at Visit 1, Visit 3 and Visit B1 in the adult population, and Visits V1a, V2a, V4p, V5p and V5ab in the adolescent population.

Women of childbearing potential are required to practice an acceptable method of birth control for the first 3 months after receiving the last dose of study vaccine. An acceptable method of birth control is defined as those, which result in a low failure rate (i.e., less than 1% per year) when used consistently and correctly. This includes 1 of the following measures:

- Hormonal contraceptives (e.g., implants, birth control pills, patches).
- Intrauterine hormone-releasing system or intrauterine device.
- Barrier type of birth control measure (e.g., diaphragms, cervical caps).

- Vasectomy in the male sex partner ≥3 months prior to first vaccination.
- Same-sex relationships.

Women who are not of childbearing potential are not required to use any birth control measure. A woman is considered of non-childbearing potential, if she is:

- Surgically sterilized for ≥3 months prior to Visit 1 (permanent sterilization methods include hysterectomy, bilateral salpingectomy or bilateral oophorectomy, or transcervical sterilization [Essure and Adiana procedures], or tubal ligation).
- Postmenopausal for ≥12 months prior to Screening.

If a participant becomes pregnant during the study, she must immediately inform the Investigator and the participant is asked to attend all remaining visits according to schedule.

11.6 Participant Withdrawal or Discontinuation

11.6.1 General

Any participant has the right to withdraw from the study at any time for any reason, without the need to justify. A participant who considers withdrawing from the study must be informed by the Investigator about modified follow-up options (e.g., telephone contact, a contact with a relative or treating physician or information from medical records).
The Investigator and Sponsor also have the right to prematurely terminate a participant's further participation in the study, e.g., in the case of non-compliance or if – in the judgment of the Investigator and/or Sponsor – continued participation would pose an unacceptable risk for the participant.

A study participant must not receive the second study vaccine, if one or more of the following criteria applies:

a) If a participant becomes pregnant during the study. Attempts will be made to follow her through completion of the pregnancy and the first 3 months of life of the new-born. The Investigator will record a narrative description of the course of the pregnancy and infant. For further information on pregnancy reporting procedures see Section 15.8.

b) If a participant has symptoms, or abnormal laboratory values, which are considered unacceptable by the participant or the Investigator.

c) If a participant experiences an SAE or AESI with no likelier alternative cause than the study vaccine (i.e., possibly or probably related). In such cases, DSMB review and evaluation or confirmation of seriousness criteria and causality assessment will be conducted.

d) If a participant experiences a severe systemic allergic reaction, e.g., generalized urticaria within 10 days after vaccine administration or anaphylaxis within 24 hours following vaccine administration, with no likelier alternative cause than the study vaccine.

e) If a participant experiences a Grade 3 solicited injection site reaction that (1) occurs within 7 days following vaccination and (2) lasts longer than 3 days.

f) If a participant experiences a Grade 3 solicited systemic reaction that (1) occurs within 7 days following vaccination and (2) lasts longer than 3 days. However, the participant may receive further vaccination if there is a more plausible alternative cause for the reaction.

g) If a participant experiences a Grade 3 unsolicited AE, including Grade 3 laboratory abnormality that is assessed to be clinically relevant by the Investigator, and has no likelier alternative cause than the study vaccine or has not resolved prior to the next scheduled study vaccination.

The primary reason for withdrawal/discontinuation of a participant from treatment and/or from the study should be documented in the electronic Case Report Form (eCRF) (e.g., withdrawal of consent, withdrawal due to AE, Investigator/Sponsor recommended withdrawal, lost to follow up, death).

Participants withdrawn from further vaccination should perform their remaining regular study visits as scheduled, if there are no other compelling reasons not to do so.
In case of premature withdrawal from the study, all attempts should be made to have the participant perform an Early Termination (ET) visit.

Data collected on withdrawn participants will be used in the analysis and included in the Clinical Study Report (CSR).

Participants who do not complete the entire study due to withdrawal or discontinuation for any reason will not be replaced.

11.6.2 Access to a nationally deployed or approved COVID-19 vaccine

It is possible that during the course of this study, participants become eligible, through the national vaccination roll-out, to receive a nationally deployed or approved COVID-19 vaccine (this then becomes standard of care vaccine). Participants can discuss with the Investigator and others to make an informed choice about whether they should take the approved COVID-19 vaccine or continue with the study.

If a participant decides to receive a nationally deployed COVID-19 vaccine, and the Investigator may unblind the participant and inform them about which vaccine they have received if this will be required for booster administration. The Investigator will also advise them on consequences and the necessity to complete the study visits according to the schedule of study procedures and assessments.

If the study participant has already received the 2 doses of the study vaccine, they will not be unblinded immediately but advised to wait until after Day 43 visit. Participants who decide to take a nationally deployed COVID-19 vaccine will be encouraged to still attend all remaining study visits and procedures. However, if the participant decides not to attend the remaining study visits, they will be asked to perform an Early Termination visit, as stated in the Schedule of Study Procedures and Assessments of this Protocol.

11.7 Lost to Follow-up

A participant will be considered lost to follow-up if she or he fails to return for scheduled visits and is unable to be contacted by the study site.

The following actions should be taken if a participant fails to return to the study site (clinic) for a required visit:

- The study site must attempt to contact the participant and reschedule the missed visit as soon as possible and counsel the participant on the importance of maintaining the assigned visit schedule and ascertain whether or not the participant wishes to and/or should continue in the study.
- Before the participant is deemed lost to follow-up, the investigator or designee must take every effort to regain contact with the participant through different channels (e.g. telephone call and registered letter). These contact attempts should be documented in the medical record.
- Should the participant continue to be unreachable, she/he will be regarded as lost to follow-up.

11.8 Re-screening of Participants

A participant is eligible for re-screening if she or he completed the screening visit and met eligibility criteria but cannot be randomized or vaccinated for administrative reasons within the given time window. In such case a new unique participant identifier number will be assigned, and the Participant Identification Log will be updated.

More details about re-screening can be found in the study EDC manual.

12. INVESTIGATIONAL MEDICINAL PRODUCT

12.1 Description of IMPs

12.1.1 VLA2001

VLA2001 is a highly purified, inactivated, whole virus SARS-Cov-2 vaccine grown on Vero cells. The vaccine production platform, developed by Valneva, uses an inactivated whole-virus approach where live wild-type virus is grown in cell culture and then inactivated (i.e. making it unable to replicate and infect cells) via chemical treatment. Valneva uses β-propiolactone inactivation in order to preserve the native surface structure of the S protein. VLA2001 has similar biological, physical and chemical properties as the approved vaccine IXIARO, produced using the same platform. The final administered volume of VLA2001 is 0.5 mL.

Table 1 Composition of VLA2001

VLA2001 (0.5mL)

**Active substance**	
SARS-CoV-2 inactivated	Antigen Units/dose, 33 AU
Excipients and buffer components	
Aluminium Hydroxide	0.5 mg Al^{3+}/dose
CpG 1018	1 mg/dose
Dulbecco's Phosphate Buffered Saline (DPBS) [3)]	
rHA	≤25 µg/dose

For this clinical study, the vaccine will be provided in multidose glass vials as a liquid formulation containing aluminium hydroxide and CpG 1018.

12.1.2 AZD1222

AZD1222 is a recombinant, replication-defective chimpanzee adenovirus expressing the SARS-CoV-2 S surface glycoprotein driven by the human cytomegalovirus major immediate early promoter that includes intron A with a human tPA leader sequence at the N terminus. AZD1222 is administered by intramuscular injection. AZD1222 will be available as multi-dose vials. The recommended dose by the manufacturer at the time of administration will be used for this study.

More details can be found in the IMP and or pharmacy manual.

12.1.3 Placebo

Placebo consists of a PBS buffer based on Dulbecco’s PBS media formulation without Calcium and Magnesium. The concentration of the PBS is 1x and is produced with raw material classified as free from animal origin. The filling volume is 0.6 mL, ensuring an extractable volume of 0.5 mL. The glass vials are 2R Type I Plus® glass vials closed with 13 mm injection Flurotec® secured by aluminium crimp caps.

**Placebo**	
Potassium Chloride (KCl)	100 µg
Potassium Dihydrogen Phosphate (KH_2PO_4)	100 µg
Sodium Chloride (NaCl)	4000 µg
Di Sodium Hydrogen Phosphate Heptahydrate (Na_2HPO_4 x$7H_2O$)	1080 µg

12.2 IMP Labelling & Packaging

Packaging and labelling of IMP is performed by a central manufacturing organization located in the UK. Labels will be written in accordance to local law.

12.3 IMP Storage

The IMP should be stored in a refrigerator at +2 to +8°C in a room with access limited to study personnel. Temperature monitoring systems will be used.

PLEASE DO NOT FREEZE!

12.3.1 Dispensing and Accountability of Investigational Medicinal Product

A drug shipment log will be kept current by the site, detailing the date and quantity of IMP received from and returned to the Sponsor. In addition, a current drug dispensing log has to be maintained, detailing the dates and quantities of IMP administered in which syringe to each participant. This documentation will be available to the designated unblinded CRA to verify drug accountability during the study and to perform overall drug accountability.

Further Details of IMP handling and return are provided in a study specific pharmacy and or IMP manual.

13. STUDY PROCEDURES

All visits will be conducted at the clinical site on an outpatient basis.

13.1 Informed Consent and Enrolment

Any participant who provides informed consent (i.e., signs and dates the informed consent form) is considered enrolled in the study. For participants aged ≥12 to <18 years written informed consent by the participant's legal representative(s), according to local requirements, and written informed assent of the participant, if applicable, is required.

The Investigator or designee will inform the participant about the procedures, risks and benefits of the study. Fully informed, written consent and assent as applicable must be obtained from each participant prior to any assessment being performed. It is important that the participant is allowed sufficient time to decide on his/her participation in the study.

During the informed consent process, trial participants will be made aware of how their confidentiality will be protected in the event of changes required by COVID-19.

13.2 Participant Identification Code

At Visit 0, a 10-digit participant identification code will be assigned to each participant. The first 4 digits are the product identifier (e.g. 2001 for this product) provided by the Sponsor. The fifth digit is the study identifier (i.e. 2001-2 for this Phase 3 study); the sixth and seventh digits are the site identification number (i.e. 2001-2-01). The last 3 digits are assigned in ascending order as the participants are enrolled (i.e., signing the informed consent form, e.g. 2001-2-01-001).

13.3 Investigational Treatment

13.3.1 Description of Treatment

The vaccination schedule consists of at least 2 vaccinations for each study participant, administered by intramuscular (IM) injection in the deltoid region of non-dominant arm preferably 28 days apart, on Day 1 and Day 29 and at visit B1 for participants in the booster group.

Adolescent participants in the VLA-group will receive a third vaccination at Visit V5ab (Day 208). Placebo-group participants will receive 4 vaccinations: placebo at Visit 1a (Day 1) and Visit 2a (Day 29), and VLA2001 at Visit V4p (Day 71) and Visit V5p (corresponds to Day 29 post first VLA2001 vaccination).

13.3.2 Vaccine Preparation and Administration

Once a participant is eligible for vaccination, the IRS and EDC system will notify the unblinded pharmacist about whether VLA2001 or AZD1222 in the adult population, or VLA2001 or placebo in the adolescent population is to be used.

The preparation of the IMP is explained in detail in the IMP manual.

Before administration, it must be verified that the participant number listed on the syringe label matches with the participant to be vaccinated.

The IMP must be administered intramuscular. Under no circumstances should the vaccine be administered intravascularly, as this could lead to immediate hypersensitivity reactions such as shock.

Anaphylaxis or other possible severe acute, post-vaccination adverse reactions to vaccines, including VLA2001 vaccine, are very rare, but can occur. Therefore, appropriate emergency equipment and medication as well as adequately trained personnel must be on site whenever a vaccination is performed.

More details about the preparation of the vaccine and the vaccination procedure can be found in the pharmacy and or IMP manual.

13.4 Post- Vaccination Observation

All participants will be observed at the study site for at least 30 minutes after vaccination.

13.4.1 Adult (>55 years of age) Sentinel Group

The first 10 Participants who are older than 55 years will be treated as a ‘sentinel’ group and will be observed for at least 60 minutes after each vaccination.

This is in order to provide appropriate emergency treatment should this be necessary. In addition, vital signs including pulse rate and blood pressure while seated and at rest will be measured prior to discharge. Any injection site and systemic reactions will be recorded.

Prior to leaving the study site, the participant will be instructed how to complete the respective electronic diary for documentation of AEs (for further information see Section 15.3), will be given a digital thermometer for measuring oral body temperature and a measuring guide for assessing injection site reactions (at the vaccination visit only).

13.4.2 Adolescent Sentinel Group

For safety reasons, the first 16 adolescents will be enrolled in an open-label, non-randomized manner. The sentinel dosing is aimed to be done at a single site to ensure permanent oversight on safety data by one principal investigator during the recruitment of the 16 sentinel vaccinees.
If, for logistical reasons, a second site will be involved in the recruitment of the sentinel participants, vaccinations will be limited to one site on a specific day. Safety data exchange between the study sites will be ensured.

The first 4 adolescents will be vaccinated 1 hour apart each. After vaccination the vaccinee will be observed for the development of any acute reaction at the study site for 3 hours after the vaccination procedure. Prior to discharge from the study site, vital signs will be measured, and the vaccinee/parents will be instructed to use the eDiary. The study site will contact the participant per phone approximately 24 and 48 hours after vaccination for safety follow-up. The provided information must be compared with the entries in the subject´s eDiary.

The minimum time before the next 4 subjects are vaccinated is therefore 24 hours after the all participants of the previous sentinel cohort have received their vaccination. The same procedure is applied for the following 3 sentinel groups.

A Data Safety and Monitoring Board (DSMB) will review the accrued safety data when all 15 sentinel subjects have completed the 7 day e-diary period after vaccination. After favourable DSMB review randomization of the remaining subjects across all sites will be initiated.

13.5 Monitoring of COVID-19 symptoms

All enrolled participants will be intensively monitored during the conduct of the study to rapidly diagnose COVID-19 and referred for treatment according to local site procedures, if applicable.

During the 7 days following administration of each dose of study intervention, investigator judgement should be used to determine which participants should initiate SARS-CoV-2 PCR testing and/or illness visits as symptoms may be due to the reactogenicity of the study intervention as opposed to potentially due to infection with SARS-CoV-2.

Even in the absence of symptoms, participants may present with a positive SARS-CoV-2 PCR result (e.g. as part of community screening, testing for travel purposes, …). In case, no symptoms are reported in association with a positive SARS-CoV-2 PCR result this should be captured in the eCRF.

Participants who develop any of the COVID-19 related symptoms (with or without presence of a positive test result for SARS-CoV-2 infection) after randomization into the study will be advised to contact the study team for evaluation and - in case not available yet - testing for

SARS-CoV-2 (PCR), preferably at the clinical study site (PCR test done at community is also acceptable) according to the following scheme:

Immediately: Participants should contact the study site immediately, in case they develop any of the following symptoms;

4. Fever (≥38.0°C or ≥100.4°F), or
5. Shortness of breath, or
6. Difficulty in breathing (wheezing, crop)

After 2 consecutive days: Participants should contact the study site in case they have at least one of the following symptoms, for at least 2 consecutive days:

- Sore throat
- Chills
- Cough
- Fatigue
- Muscle aches
- Body aches
- Headache
- New loss of taste
- New loss of smell
- Nasal Congestion
- Runny nose
- Nausea
- Vomiting
- Diarrhoea

In case of an initial negative PCR test result, participants will be invited for a second confirmatory PCR test on site (i.e. COVID-19 verification visit, see section 21.1) after approximately 2 days. If the test can not be done at site, a test in the community is also acceptable. In the event the result is still negative, the participant will continue with scheduled visits.

If any of the tests is positive, the participant will be asked to come for a COVID-19 illness visit at site as soon as possible (if the national quarantine protocol allows it). Where necessary and possible, the COVID-19 illness visit may be substituted by a home or mobile visit.

As part of the illness visit, blood samples will be taken for antibody assessment. Either a nasal swab or gargle (saliva) sample will be taken for genetic sequencing for identification of the virus strain. In addition, a recording of COVID-19-related clinical and laboratory information as shown in the Schedule of Assessments will be done.

The Investigator's severity grading of COVID-19 will be based on the following case definition criteria:

Case Definition for Mild COVID-19

A SARS-CoV-2 positive RT-PCR result

AND:

One of the following symptoms:

- Fever (≥38.0°C or ≥100.4°F),
- Sore throat,

- Malaise (loss of appetite, generally unwell, fatigue, physical weakness),
- Pain (headache, muscle pain (myalgia)),
- gastrointestinal symptoms,
- Respiratory symptoms (cough, chest congestion, runny nose, wheezing),
- Skin rash,
- Eye irritation or discharge,
- Shaking, Chills, or rigors
- New or changing olfactory or taste disorders,
- Red or bruised looking feet or toes.

Case Definition for Moderate COVID-19

A SARS-CoV-2 positive RT-PCR result

AND

Any 1 of the following new or worsening signs or symptoms:

- Respiratory rate ≥20 breaths/minute,
- Abnormal saturation of oxygen (SpO_2) but still >93% on room air at sea level or adjusted according to altitude,
- Clinical or radiologic evidence of pneumonia,
- Radiologic evidence of deep vein thrombosis (DVT)
- Shortness of breath or difficulty breathing.

OR

Any 2 of the following new or worsening signs or symptoms:

- Fever (≥38.0°C or ≥100.4°F),
- Heart rate ≥90 beats/minute,
- Shaking, chills, or rigors,
- Sore throat,
- Cough,
- Malaise, as evidenced by 1 or more of the following:
  - Loss of appetite,
  - Generally unwell,
  - Fatigue,
  - Physical weakness.
- Headache,
- Muscle pain (myalgia),
- Gastrointestinal symptoms (e.g. diarrhoea, vomiting, nausea, abdominal pain)
- New or changing olfactory or taste disorders
- Red or bruised looking feet or toes.

Having 2 or more elements of a symptom (e.g., vomiting and diarrhoea or fatigue and loss of appetite) is counted only as 1 symptom for the case definition. To meet the case definition, a participant would need to have at least 2 different symptoms.

Case Definition for Severe/Critical COVID-19

A SARS-CoV-2 positive RT-PCR result

AND

Any 1 of the following at any time during the course of observation:

- Clinical signs at rest indicative of severe systemic illness (respiratory rate ≥30 breaths/minute, heart rate ≥125 beats/minute, oxygen saturation (SpO_2) ≤93% on room air at sea level, or partial pressure of oxygen/fraction of inspired oxygen (PaO_2/FiO_2) <300 mmHg)

  (SpO_2 criteria will be adjusted according to altitude per the investigator judgement),
- Respiratory failure (defined as needing high-flow oxygen, non-invasive ventilation, mechanical ventilation, or extracorporeal membrane oxygenation [ECMO]),
- Evidence of shock (defined as systolic blood pressure <90 mmHg, diastolic blood pressure <60 mmHg, or requiring vasopressors),
- Significant acute renal, hepatic, or neurologic dysfunction,
- Admission to the ICU,
- Death.

All cases meeting the severe/critical criteria will be reviewed by the Data and Safety Monitoring Board (DSMB) to determine if the case is severe/critical in their judgement.

13.6 Monitoring for blood clotting events following COVID-19 vaccination

Participants will be advised to contact the study site if they experience any of the following symptoms more than 4 days and within 28 days of vaccination:

- new onset of severe headache, which is getting worse and does not respond to simple painkillers
- an unusual headache which seems worse when lying down or bending over, or may be accompanied by blurred vision, nausea and vomiting, difficulty with speech, weakness, drowsiness or seizures
- new unexplained pinprick bruising or bleeding
- shortness of breath, chest pain, leg swelling or persistent abdominal pain

An unscheduled in person visit will be held if deemed necessary by the Investigator. As described in 13.7.1 and 22.1, procedures at unscheduled visits include safety labs (e.g., platelet counts), additional examinations/ assessments are performed as deemed appropriate by the investigator. Based on the assessed findings, the investigator will determine further monitoring and medical treatment. The sponsor will ensure that the most up to date guidelines on the handling of blood clotting following COVID-19 vaccination endorsed by MHRA is communicated to the participating sites during the study conduct.

13.7 Screening and Study Visits

Please refer to the Schedule of Study Procedures and Assessments in Section 22.1 for timings of all procedures to be carried out during the study.

Please note:

Study Changes in Response to the COVID-19 Pandemic Situation

Authorities worldwide have issued guidance for Industry, Investigators, and Institutional Review Boards on conducting clinical studies during the COVID-19 pandemic.

Competent health authorities and agencies, including the FDA, the MHRA and the HRA, support the implementation of remote activities to ensure continuation of study activities should the COVID 19 situation limit/forbid access to clinical sites.

Valneva will continuously monitor and evaluate the development of the COVID-19 pandemic in the area of study sites to determine if any measures should be implemented to mitigate undue risks to the participants or in response to local governmental recommendations.

Such measures can include (but are not limited to):

- Remote monitoring,
- Switching in-person visits to telephone call and/or home visits (including appropriate documentation of the phone call/home visit),
- Sending 'Dear Investigator' letters to inform Investigator sites of changes to trial conduct
- Self-isolation of trial participants as a precaution or as a result of confirmed infection, limiting or inhibiting the required clinical trial activities.

Explicit instructions from Valneva and the CRO as to what can be accessed and where, will be available and communicated in a timely manner to ensure fulfilment of protocol activities during the trial.

The safety of the participants participating in the trial remains our priority.

*For more information see the link to MHRA Guidance in the references section

13.7.1 Unscheduled Visit

An unscheduled visit can be held at any time during the study if deemed necessary by the Investigator (e.g. follow-up on unexpected AEs or SAEs) or the DSMB. Assessments performed at an unscheduled visit will be either at the Investigator's or DSMB's discretion. Unscheduled visits and any procedures/assessments performed during such a visit (e.g. physical examination, laboratory test) should be documented in the source data and the eCRF.

In any case a phone call will take place if a) participants have not entered data into the eDiary; b) a grade 3 event is reported by the study participant via the eDiary, c) if a serious AE is reported via the eDiary, d) if participants indicate symptoms towards potential COVID-19 infection.

13.7.2 Early Termination Visit

Participants who terminate participation or who are withdrawn from the study prematurely will undergo investigations during an Early Termination (ET) visit, if possible. Every effort should be made to have discontinued participants complete the ET visit.

Please refer to the section on Schedule of Study Procedures and Assessments for more details.

If an in-person ET Visit is not possible, a phone call should be made as soon as possible after termination to capture at least concomitant medications and unsolicited AEs and review of reported solicited AEs since the last study visit.

The reason for early termination should be clarified in as much detail as possible. If an AE was the reason for early study termination, details of the specific AE(s) should be captured (see Section 11.6). The reason for discontinuation will be recorded on the eCRF, and data collected up to the time of discontinuation will be used in the analysis and included in the clinical study report.

In the event of participant discontinuation due to an AE, clinical and/or laboratory investigations that are beyond the scope of the required study observations/assessments may be performed as part of the evaluation of the event. These investigations will take place under the direction of the Investigator in consultation with the Sponsor, and the details of the outcome may be reported to the appropriate regulatory authorities by the Sponsor.

13.8 Procedures for Monitoring Participant Compliance

In general, study procedures are to be performed under the supervision of the Investigator or designee at the study site. Participant compliance will be monitored by reviewing eDiary completion and other follow-up and monitoring methods.

14. ASSESSMENT OF IMMUNOGENICITY

All serum samples obtained for the determination of neutralising antibodies and binding IgG antibodies against SARS-CoV-2 will be handled according to the procedures supplied to each investigative site for the preparation, storage, and shipment of samples (refer to Laboratory Manual). Immunogenicity samples will be collected from all participants at the time points indicated in Section 22.1. Each clinical study site will be responsible for the separation of serum from whole blood samples and the safe and controlled storage of serum samples prior to shipment to the central laboratory.

Immunogenicity sample (20 mL for adults, 10 mL for adolescents) will be drawn for SARS-CoV-2-specific neutralising antibody and IgG antibody titre evaluation. An authorized laboratory will measure neutralising antibodies to SARS-CoV-2 using wild-type virus neutralising assay. In addition to the functional assay, samples will be analysed for IgG against SARS-CoV-2 by ELISA.

For the assessment of cell mediated responses, PBMCs will be isolated from 50mL (adults) or 30 mL (adolescents) whole blood in a subset of participants. PBMCs will be used to assess T-cell responses after in vitro stimulation with SARS-CoV-2 antigens (e.g. Spike protein, nucleocapsid and membrane protein) using e.g. ELISpot (IFNγ) or intracellular cytokine staining on CD4+ and CD8+ T cells (Th1: IFNγ, IL-2, TNFα and Th2: IL-4, IL-5, IL-13).

At the end of the study, results of immunogenicity assessments will be provided to the Investigator.

14.1 Additional Testing Procedures

Residual Samples:

Samples obtained in this study may, in addition to its use for assessment of SARS-CoV-2 specific antibody and cellular immune responses, also be used for further development of the vaccine, including but not limited to the following:

- Development of additional neutralization assays (e.g. pseudovirion neutralization assay)
- Assessment of neutralising antibodies against SARS-CoV-2 variants
- Detection of antibodies by enzyme linked immunosorbent assay (ELISA) against different SARS-CoV-2 antigens
- Clinical diagnostic work-up.

Such development may occur at laboratories other than the central analytical laboratories used for this study.

15. ASSESSMENT OF SAFETY

15.1 Definitions

15.1.1 Adverse Events

An AE is defined as any untoward medical occurrence in a participant administered an investigational product that does not necessarily have a causal relationship with the treatment. All new abnormalities or any exacerbation in intensity or frequency (worsening) of a pre-existing condition during or after vaccination have to be documented as TEAEs. TEAEs are defined as AEs with onset or worsening date on or after the first day of exposure to randomized treatment. Any AEs occurring before receipt of IMP will be captured as Medical History and will not be separately analysed. If an existing stable condition worsens after the administration of the IMP, they will be regarded as TEAEs.

15.1.2 Serious Adverse Event

An SAE is defined as any untoward medical occurrence that at any dose meets one or more of the following criteria:

- Outcome is fatal/results in death (including foetal death).
- Is life-threatening – defined as an event in which the participant was, in the judgment of the Investigator, at risk of death at the time of the event; it does not refer to an event that hypothetically might have caused death had it been more severe.
- Requires inpatient hospitalisation or results in prolongation of an existing hospitalisation.
- Results in persistent or significant disability/incapacity (i.e., a substantial disruption of a person's ability to conduct normal life functions).
- Results in congenital anomaly/birth defect.
- Is a medically important condition – a medical event that may not be immediately life-threatening or result in death or require hospitalisation but may jeopardize the participant or may require medical or surgical intervention to prevent one of the other outcomes listed in the definitions above. This definition also applies to progression of disease leading to a serious outcome.

In case of hospitalisation or prolonged hospitalisation for diagnostic or elective medical procedures that were planned prior to vaccination to treat a pre-existing condition that did not change in severity, neither the condition leading to the hospitalisation or prolonged hospitalisation, nor the actual medical procedure need to be reported as an SAE. In this case, the underlying diagnosis or condition should be reported in the medical history section of the eCRF and the corresponding medical procedure should be documented as a comment to the underlying diagnosis or condition in the medical history section of the eCRF.

The Sponsor will classify the SAEs as either expected or unexpected:

Expected:	An AE that is listed in the current Investigator's Brochure (IB)
Unexpected:	An AE that is not listed in the current IB, or it differs because of greater severity or greater specificity

For the purpose of this study, AEs graded as potentially life-threatening (Grade 4) will be reported as SAEs.

15.1.3 Adverse Events of Special Interest

An AESI (serious or non-serious) is an event of scientific and medical concern specific to the Sponsor's product. Please refer to section 22.5, where symptoms/illnesses considered as an AESI for this study are described.

15.1.4 Medically-Attended Adverse Event

All AEs where participants are seeking medical care (i.e. doctor's office, emergency service, hospital, but not including use of self-medication).

15.1.5 Pre-existing Diseases

Pre-existing diseases that are present before entry into the study, that are described in the medical history, and that manifest with the same severity, frequency, or duration after vaccine exposure, will not be recorded as AEs. Furthermore, routine health checks required due to pre-existing diseases will not be recorded. However, when there is an increase in the severity of a pre-existing disease, the event must be described on the AE CRF page.

15.1.6 Untoward Medical Occurrences Not Considered Adverse Events

Each untoward medical occurrence experienced before vaccine exposure (for example, from the time of signed informed consent up to but not including vaccine exposure) will be described in the medical history.

15.2 Collection, Documentation and Assessment of Adverse Events

15.2.1 Unsolicited Adverse Events

Participants will be provided and trained to use an electronic diary to collect unsolicited AEs occurring until Day 43 and for those, who are boostered up to 6 months after the booster. Additionally, the Investigator will enquire about AEs during study visits throughout the study. Clinically relevant laboratory parameter changes constitute unsolicited AEs, too, unless they are considered a symptom of an underlying AE or part of a syndrome that is reported as AE (e.g., presence of blood cells in urine in a person diagnosed with urinary tract infection). In addition, symptoms noted during the symptom-driven physical exams (unless already covered by an AE) constitute AEs.

All unsolicited AEs need to be documented in the respective AE section of the eCRF during the applicable study visit, regardless of their source (AEs noted in the participant diary [see Section 15.3], open question to participant, laboratory parameters, symptom-driven physical examination). Serious adverse events as well as AESIs will continue to be documented in the eCRF until the end of the study.

Any symptom is regarded as a separate AE. However, if the Investigator considers several symptoms to be in the context of one underlying diagnosis, the Investigator may merge these symptoms into one single appropriate AE. The AE term entered into the eCRF should contain all symptoms summarized to one event (e.g., 'Influenza with flu-like-symptoms, fever and headache').

The Investigator will follow-up each AE until it is resolved or until the medical condition of the participant is stable. All relevant follow-up information will be reported to the Sponsor until the end of the study for each participant. Serious adverse events and AESIs ongoing after this last visit (e.g. Visit 7 for non-boostered adults) will be followed until resolution or achievement of stable clinical conditions, latest until the overall end of the study.

The following information will be documented for each AE: severity, causality, outcome, and seriousness, medically-attended, action taken to treat AE, action taken on IMP, start and stop dates.

15.2.2 Severity

The Investigator will assess the severity of AEs using his/her clinical expertise and judgment based on the most appropriate description below:

Mild (Grade 1):	Awareness of signs or symptoms, but easily tolerated, does not interfere with daily activities.
Moderate (Grade 2):	Discomfort enough to interfere with usual activity and with or without requiring medical intervention.
Severe (Grade 3):	Incapable of work or usual activity and requiring medical intervention.
Life-threatening (Grade 4):	Urgent intervention indicated and required.

15.2.3 Causality Assessments

Causality is a determination of whether there is a reasonable possibility that the vaccine administration is aetiologically related to/associated with the AE.

Solicited adverse events of vaccination site reactions will be considered causally related to study treatment.

For all other adverse events, the Investigator will determine a **causal relationship** to the study vaccine. A number of factors will be considered in making this assessment, including: 1) the temporal relationship of the event to the administration of the study vaccine 2) whether an alternative aetiology has been identified and 3) biological plausibility.

Causality of all adverse events should be assessed by the Investigator using the following question:

“Is there a reasonable possibility that the adverse event may have been caused by the study vaccine?”

- YES (related): There is a reasonable possibility that the study vaccine contributed to the adverse event
    - Probable: Reaction that follows a reasonable temporal sequence from administration of the IMP, or that follows a known or expected response pattern to the suspected treatment; and that could not reasonably be explained by known characteristics of the participant’s clinical state.
    - Possible: Reaction that follows a reasonable temporal sequence from administration of the IMP, or that follows a known or expected response pattern to the suspected treatment; but that could readily have been produced by a number of other factors.
- NO (not related): There is no reasonable possibility that the adverse event is causally related to the administration of the study vaccine. There are other, more likely causes and administration of the study vaccine is not suspected to have contributed to the adverse event
    - Unlikely: Reports not following a reasonable temporal sequence from administration of the IMP; an event, which may have been produced by the participant’s clinical state or by other environmental factors. A more likely alternative aetiology exists.
    - Not related (unrelated): Events for which sufficient information exists to conclude that the aetiology is unrelated to the IMP.

Adverse events with a causality reported as probable or possible will be considered related to the IMP. Adverse events with missing causality assessment will be regarded as related unless further specified. All other AEs will be considered as not related to IMP.

15.2.4 Assessment and Outcome of Adverse Events

Each AE that occurs from first vaccination to study completion/termination will be described in the eCRF (i.e. one AE per form) using the medical diagnosis (preferred), symptom, or sign, in standard medical terminology in order to avoid the use of vague, ambiguous, or colloquial expressions. Adverse events will be evaluated by the Investigator for:

- Seriousness defined in Section 15.1.2.
- Severity as defined in Section 15.2.2.
- Causal relationship to vaccine exposure as defined in Section 15.2.3.

For each AE, the outcome will also be documented as either:

- Recovered/Resolved
- Recovered/Resolved with sequelae
- Recovering/Resolving
- Not recovered/not resolved
- Fatal
- Unknown

If the severity rating for an ongoing AE changes before the event resolves, the AE will not be reported a second time. Instead the original AE report will be revised. For purposes of data capture the highest severity rating during the course of a single AE will be the severity rating entered on the AE CRF.

NOTE: A participant's death per se is not an event, but an outcome. The event that resulted in the participant's death must be fully documented and reported, regardless of being considered related to treatment or not.

15.2.5 Solicited Adverse Events

Solicited AEs (predefined terms) will be collected in the eDiary by the participant, starting on the day of vaccination and during the follow-up period after each vaccinations (i.e. seven consecutive days). Thereafter participants will be advised to report to the site any AEs, as it happens to the site throughout the study duration.

Solicited AEs will be reviewed and if necessary re-graded for severity by the Investigator or the designee, according to the FDA’s Toxicity Grading Scale for Healthy Adult and Adolescent Volunteers Enrolled in Preventive Vaccine Clinical Trials, see Table 2, Table 3 and Table 4.

For solicited AEs which are serious and/or medically attended, the investigator carries out more detailed assessments and documents them in the eCRF. For solicited local and systemic AEs persisting beyond 6 days after vaccination, stop date is also documented in the eCRF (AE section).

15.2.6 Injection Site Reaction – Measurement and Evaluation

Solicited injection site reactions include injection site pain, itching, tenderness, redness and swelling/induration. Participants will be provided with a measuring tool to measure the size of any measurable injection site reaction (longest diameter).

Table 2 Grading of Injection Site Reactions

**Vaccine-specific Criteria**	**Mild (Grade 1)**	**Moderate (Grade 2)**	**Severe (Grade 3)**	**Potentially Life Threatening (Grade 4)[c]**
Pain	Does not interfere with activity	Repeated use of non-narcotic pain reliever >24 hours or interferes with activity	Any use of narcotic pain reliever or prevents daily activity	Emergency room visit or hospitalisation
Tenderness	Mild discomfort to touch	Discomfort with movement	Significant discomfort at rest	Emergency room visit or hospitalisation
Erythema / Redness [a]	2.5–5.0 cm<br>0.98–1.96 inch	5.1–10.0 cm<br>1.97–3.94 inch	>10.0 cm<br>>3.94 inch	Necrosis or exfoliative dermatitis
Induration / Swelling [b]	2.5–5.0 cm<br>(0.98–1.96 inch)<br>and does not interfere with activity	5.1–10.0 cm<br>(1.97–3.94 inch) or interferes with activity	>10.0 cm<br>(>3.94 inch) or prevents daily activity	Necrosis

[a] In addition to grading the measured local reactions at the greatest single diameter.

[b] Induration/swelling should be evaluated and graded using the functional scale as well as the actual measurement.

[c] Any Grade 4 injection site reaction will be reported as SAE, Grade 4 severity be documented in the eCRF according to the eCRF manual or completion guidelines.

15.2.7 Systemic Reactions – Measurement and Evaluation

Solicited systemic reactions include fever/body temperature, fatigue, headache, nausea/vomiting, muscle pain.

Table 3 Grading of Systemic Reactions

Vaccine-specific Criteria	Mild (Grade 1)	Moderate (Grade 2)	Severe (Grade 3)	Potentially Life Threatening (Grade 4)[d]
Nausea/vomiting	No interference with activity or 1 to 2 episodes/ 24 hours	Some interference with activity or >2 episodes/24 hours	Prevents daily activity, requires outpatient IV hydration	Emergency room visit or hospitalisation for hypotensive shock
Headache	No interference with activity	Repeated use of non-narcotic pain reliever >24 hours or some interference with activity	Significant; any use of narcotic pain reliever or prevents daily activity	Emergency room visit or hospitalisation
Fatigue	No interference with activity	Some interference with activity	Significant; prevents daily activity	Emergency room visit or hospitalisation
Myalgia	No interference with activity	Some interference with activity	Significant; prevents daily activity	Emergency room visit or hospitalisation

[a] Any Grade 4 systemic reaction will be reported as SAE, Grade 4 severity should be documented in the eCRF according to the eCRF manual or completion guidelines.

15.2.8 Fever and Body Temperature

Participants will be given a digital thermometer to measure their body temperature orally once every evening from the day of vaccination until Day 7 after each vaccination. In case fever (oral body temperature ≥ 38.0°C) occurs, participants should measure their body temperature every 4 to 8 hours until fever resolves (oral body temperature < 38.0°C). The time at which the first body temperature of <38.0°C is recorded is considered to be the end of the fever episode. All fever measurements should be recorded by the participant in the participant diary including the first value that shows a return to normal body temperature. If more than one body temperature value is recorded in the participant diary on a given day, the highest daily temperature reading will be recorded in the eCRF.

Table 4 Grading of Fever

Mild	38.0°C – 38.4°C
Moderate	38.5°C – 38.9°C
Severe	39.0°C – 40.0°C
Potentially Life-Threatening	>40.0°C

15.3 Electronic Diary

Electronic diaries (eDiary) will be used to record solicited AEs starting on the day of vaccination and during the follow-up period after each vaccination for 7 consecutive days.

Thereafter participants will be advised to report any AEs, as it happens to the site throughout the study duration.

Assessments by the participant should occur at the same time each day, starting approximately 8 hours after vaccination. The participant will be properly instructed on the reporting requirements and how to complete and use the eDiary, thermometer, and measuring tool (for assessment of measurable injection site reactions).

The following information will be collected:

- Oral body temperature.
- Solicited injection site reactions (predefined terms).
- Solicited systemic reactions (predefined terms).
- Symptoms Relating to SARS-CoV-2 infection.
- Other AEs, i.e. unsolicited events.
- Any new medication or changes in medication taken after vaccination.

Study staff will be prompted to perform a phone call visit if:

a) no diary data are being reported by the participant,

b) a Grade 3 solicited event has been reported,

c) criteria for an SAE have been met and/ or

d) symptoms relating to SARS-CoV-2 have been reported.

The eDiary will serve as source documentation. Entries in the diaries will be transferred onto the appropriate eCRFs electronically. Any entry on the eCRF that does not correspond with an entry in the diary will be explained by the Investigator on the relevant diary page.

More details is available in the e-Diary manual and during the training of site personnel and participants.

15.4 Medical, Medication, and Non-Drug Therapy History

15.4.1 Medical history

At screening, the participant's medical history will be recorded accordingly.

In addition, the medical history will include the following:

- Previous SARS-CoV-2 infection.
- Information on planned hospitalisations (including elective surgery) during the study for medical conditions existing prior to or at study entry. Conditions leading to such planned hospitalisations do not need to be reported as SAEs.

  Detail information is available in the e-CRF completion manual and guidelines.

15.4.2 Concomitant Medications and Non-Drug Therapies

All medications or vaccines (including over-the-counter or prescription medicines) received from 2 weeks prior to study enrolment until completion/termination must be recorded in the eCRF along with the information listed below. Vitamins and/or herbal supplements are not to be recorded.

- Product name
- Reason for use

- Dates and routes of administration including start and end date
- Dosage information including dose frequency

The Study Medical Monitor should be contacted if there are any questions regarding concomitant or prior therapy.

In addition, medications to treat SAEs will be reported to the Sponsor on SAE Report Forms as described in Section15.8.1. In the context of this study, information on non-drug therapies will only be collected in relation to SAEs.

The following medications are **not permitted** if administered within the specified study periods (unless such treatment has to be administered in an emergency situation.

- NOTE: for participants who become hospitalized with COVID-19, receipt of licensed treatment options and/or participation in investigational treatment studies is permitted
- Receipt of any vaccine (licensed or investigational) other than licensed influenza vaccines within 28 days prior to and after administration of study intervention (except for medical emergencies such as tetanus or rabies exposure).
- Investigational products indicated for the treatment or prevention of SARS-CoV-2 or COVID-19.
- Another IMP.
- Immunosuppressive treatment during the course of the study (unless such treatment has to be administered in an emergency situation). Note: Specifically, treatment that can be expected to influence immune response. Such treatment includes, but is not limited to, systemic or high dose inhaled (>800 µg/day of beclomethasone dipropionate or equivalent) corticosteroids, radiation treatment or other immunosuppressive or cytotoxic drugs. Use of inhaled (low dose), intranasal or topical steroids is permitted
    - Glucocorticoids at a dose ≥20 mg/day of prednisone or equivalent given daily or on alternate days for ≥14 consecutive days between randomization and the participant´s schedule
    - Other systemically administered drugs with significant immunosuppressive activity, such as azathioprine, tacrolimus, cyclosporine, methotrexate, or cytotoxic chemotherapy between randomization and the participant´s schedule
- Any blood products or immunoglobulins.

For documentary purposes, any of the treatments listed above (including emergency treatment) given within these time periods requires special documentation and is to be documented as a protocol deviation.

Use of any other medications or non-drug therapies is not restricted.

Additionally, medications that are not permitted prior to study enrolment, resulting in exclusion from the study, are reflected in the exclusion criteria in Section 11.2.

15.5 Vital Signs

Vital signs will include body temperature (°C) measured digitally and orally according to NHS method, pulse rate (beats/min), and systolic and diastolic blood pressure (mmHg) while seated and at rest.

Vital signs will be measured at the times outlined in Section 22.1. At vaccinations visits (Day V1, V3, B1, B2 and B3 (adults), and Visits V1a, V2a, V4p, V5p and V5ab (adolescents) these data should be recorded prior to vaccination and in addition, pulse rate and blood pressure should be assessed after vaccination while seated and at rest after a 30-minute observation period (60 minutes for the first 10 participants aged 55 years and older).

Vital sign values are to be recorded on the appropriate eCRF. For each vital sign value, the Investigator will determine whether the value is considered an AE (see definition in Section 15.1.1). If assessed as an AE, the medical diagnosis (preferably), symptom, or sign, will be recorded on the AE CRF. Additional tests and other evaluations required to establish the significance or aetiology of an abnormal result, or to monitor the course of an AE should be obtained when clinically indicated. Any abnormal value that persists should be followed at the discretion of the Investigator.

15.6 Physical Examinations

At screening (Visit 0 or V0), a physical examination will include, but will not be limited to assessment of height and weight, general appearance, head and neck, eyes and ears, nose and throat, chest, lungs, heart, abdomen, extremities and joints, lymph nodes, skin, and neurological. They will be described as normal or abnormal in the e-CRF.

At all study visits, a symptom-driven physical examination will be performed if the participant reports a symptom. In addition, at the Screening Visit (Visit 0 or V0) a system-based assessment will be performed for a detailed check of the affected body system(s) and at visits, where a vaccination is given, a symptom-driven physical examination shall be performed before the vaccination is given. Another symptom-driven examination should be performed if the participant has complaints within the observation time after vaccination (See Section 22.1).

Abnormal conditions detected at screening or prior to vaccination at Visit 1 or V1 will be recorded as medical history. At all other study visits, if a new abnormal or worsened abnormal pre-existing condition is detected, the condition will be recorded as an AE.

15.7 Clinical Laboratory Parameters

Blood and urine samples will be obtained for assessment of clinical laboratory parameters as outlined in the table in the section Schedule of Study Procedures and Assessments. Laboratory samples will be analysed by certified laboratories, according to the applicable laboratory standard operating procedure (SOP). On vaccination visits samples are collected before the vaccination is given.

For women of childbearing potential, a urine sample test for pregnancy should be performed at the site using the kit supplied for this clinical study.

A baseline safety laboratory blood and urine sample will be obtained at Visit 0 or V1 (screening visit) from all participants for standard clinical chemistry, haematology, coagulation panel and urinalysis.

A SARS-CoV-2 antibody test will be done at visit 0 (screening).

A SARS-CoV-2 antigen test will be done at visit 1 (randomization).

Clinical chemistry (approx. 8.0 mL))	Creatinine, sodium, potassium, calcium, aspartate transaminase (AST), alanine aminotransferase (ALT), alkaline phosphatase, bilirubin and C-reactive protein (CRP).
Haematology panel (approx. 9.0 mL)	Haemoglobin, haematocrit, erythrocyte count, white blood cell (WBC) count, differential WBC count (basophils, eosinophils, lymphocytes, monocytes, neutrophils), platelets, erythrocyte sedimentation rate (ESR).
Coagulation panel (approx. 5 mL)	Small blood coagulation (prothrombin time, activated partial thromboplastin time and fibrinogen).
Urinalysis	Standard urine dipstick for determining pH-value, specific gravity, leucocytes, nitrite, protein, glucose, ketones, urobilinogen, bilirubin, erythrocytes.

Laboratory values will be evaluated at unscheduled safety labs in case of an AE according to the Toxicity Grading Scale for Healthy Adult and Adolescent Volunteers Enrolled in Preventive Vaccine Clinical Trials (FDA). For the individual toxicity criteria refer to Section 22.4.

Laboratory assessments for which no severity grading is described in Section 22.4 are graded as described in Section 15.2.2 upon the Investigator's judgement.

The Investigator's assessment of each abnormal laboratory value, including its clinical significance, is to be recorded in the eCRF:

- Abnormal laboratory assessments that are considered clinically relevant, in the opinion of the Investigator, need to be documented as unsolicited AEs and assessed further for severity according to the toxicity grading scale provided in Section 22.4, causality and other assessments done for unsolicited AE (see Section 15.2.1).
- Abnormal laboratory assessments that are considered a symptom of an underlying AE or part of a syndrome that is reported as AE (e.g. presence of blood cells in urine in a person diagnosed with urinary tract infection) do NOT additionally need to be documented as unsolicited AE, but a respective comment should be added to the underlying AE.

Additional tests and other evaluations required to establish the significance or aetiology of an abnormal laboratory result or to monitor the course of an AE should be obtained when clinically indicated. Any abnormal value that persists should be followed at the discretion of the Investigator.

15.8 Safety Reporting Procedures and Oversight

All AEs will be captured in the source documents from the time a signed and dated informed consent form (ICF) is obtained until the end of the study/early withdrawal. Clinically relevant medical events not meeting the above criteria and occurring between ICF signature and the first vaccination will be collected on the medical history electronic case report form (eCRF) page as pre-existing conditions. The sponsor will evaluate any safety information that is spontaneously reported by an Investigator beyond the time frame specified in the protocol. All AEs will be followed until resolution or until clinically stable.

15.8.1 Serious Adverse Events and Adverse Events of Special Interest

Any SAE and AESIs should be reported via the Safety Gateway within EDC-system within 24 hours after the Investigator has become aware of the event.

Under certain circumstances the initial notification could be done by phone, but nevertheless a written SAE/ AESI Report Form has to be submitted to the Safety Officer within 24 hours.

The study centre will be provided with specific reporting procedures. SAEs/ AESIs will be entered into the SAE/ AESIs eCRF page using a recognized medical term or diagnosis that accurately reflects the event. All identified participant's documents supporting the SAE/ AESIs should be sent to the CRO as soon as they are requested by the Medical Monitor/Drug Safety Officer by e-mail (preferable) or by fax using the contact information on section 3.

Fatal or life-threatening SAEs that the Investigator suspects are related to the study vaccine should be telephoned to the local Medical Monitor immediately upon the Investigator's awareness of the event.

All SAEs/ AESIs are reported to the Sponsor for the entire study period.

SAE/ AESIs term should represent diagnosis. Only in case no diagnosis can be identified, each symptom should be reported separately.

Medical or diagnostic procedures due to an underlying disease or symptom are not considered an AE but a consequent measure following an AE. A correct SAE report will therefore have to specify the disease or symptom as the reportable AE and the medical or diagnostic procedure as action taken.

In addition, expedited and periodic reporting to Competent Authorities and ECs will be performed in accordance with local requirements. Further reporting details can be found in the study-specific SAE procedure which is in accordance with respective EU requirements, ICH GCP, national laws and site-specific requirements. Serious adverse events that are considered as probably or possibly related and additionally are unexpected need to be reported according to the requirements for suspected unexpected serious adverse reactions (SUSARs).

Serious adverse event/ AESI reports will be reviewed by the study site's physician, the Medical Safety Officer, the Study Medical Monitor, the Sponsor Medical Officer, and the independent DSMB.

15.8.2 Pregnancy

The risk of maternal to foetal transmission of SARS-CoV-2 during pregnancy cannot be excluded. Women are strongly advised not to become pregnant during the entire duration of the study. If pregnancy occurs after receiving the first or second dose of the vaccination, the participant will be advised to continue with the study visits but will be given no more vaccinations.

Reporting requirements start with administration of the vaccination until study completion (or ET Visit). All pregnancies that occur during the clinical study period will be followed-up for 3 months after delivery or termination of the pregnancy. Any effect on either mother or foetus should be determined. A pregnancy which led to a congenital anomaly/birth defect must be followed-up by the Investigator longer or until resolution or stabilization. Duration of prolonged follow-up will be decided on an individual basis and in accordance with the Sponsor. The Investigator will prepare a narrative on the course of the pregnancy and the outcome.

The Investigator should report pregnancies within 24 hours of being notified using the Pregnancy Report Form. Reporting procedures are similar to SAE reporting procedures (contacts and processing), although a pregnancy is not considered an SAE.

If a seriousness criterion applies in addition to the pregnancy (e.g. hospitalisation, congenital anomaly/birth defect) the pregnancy qualifies as an SAE. In such case a Pregnancy Report Form **and** an SAE Report Form have to be filled out.

15.8.3 Data Safety Monitoring Board

An independent DSMB, consisting of experts that will be selected based upon their expertise in infectious diseases (preferably clinical experience with COVID-19) vaccines clinical research and/or clinical medicine, will be utilised in this study to provide independent monitoring of safety data/issues during the course of the study and make recommendations to the Sponsor regarding further conduct of the study, further vaccinations in the study and/or protocol modifications to be implemented for safety reasons. A DSMB charter including a detailed description will be prepared.

The first 10 Participants who are older than 55 years will be treated as a 'sentinel' group and will be observed for at least 60 minutes after each vaccination. In addition, there will be a dedicated DSMB meeting after the first 10 participants above 55 years of age received their first vaccination.

In the adolescent population the DSMB will review the accrued safety data when all 16 sentinel participants have completed the 7 day e-diary period after vaccination. After favourable DSMB review randomization of the remaining participants across all sites will be initiated.

Responsibilities of the DSMB:

- Review of accrued safety data of the first 10 sentinel participants (age 55+) as stated above.
- Review of accrued safety data of all adult participants once adults have completed at least 1 month safety follow-up after the second dose of VLA2001.Review of accrued safety data of the first 16 sentinel participants (aged ≥12 to <18 years) as stated above.
- Review of any case reports of SAEs/AESIs on an ad-hoc basis.
- Periodically review unblinded listings and summary tabulations of SAEs, deaths, solicited AEs, unsolicited AEs and AEs leading to withdrawal from further vaccinations.
- All COVID-19 cases meeting the severe/critical criteria will be reviewed by the Data and Safety Monitoring Board (DSMB) to determine if the case is severe/critical in their judgement.
- Participate in ad-hoc DSMB reviews initiated by the principal Investigator, the Sponsor or the medical monitor at the CRO for any safety reasons. During any of its meetings, the DSMB can issue a recommendation to stop or halt the study or to discontinue a treatment group, e.g. in response to an excess rate of AEs with the same suspected underlying pathological mechanism.

More details about the DSMB procedures is available in the study-specific DSMB charter.

15.8.4 Sponsor

Listings of available blinded safety data will be closely reviewed by the Sponsor to identify any potential safety concerns until the last participant has completed the study. Further details about safety monitoring will be specified in a medical monitoring plan.

16. STATISTICAL ANALYSIS

A first statistical analysis, including the analysis of the superiority primary endpoint, will be performed after the last adult participant has completed the Day 43 visit.

A second statistical analysis will be performed when all adult participants have completed Visit 6 (Day 208) and the adult participants from the booster immunogenicity subset have completed visit B2.

A third statistical analysis will be performed when all adolescent participants have completed Visit V3a.

A fourth statistical analysis will be performed at the time when adolescent participants have completed Visits V6p and V6ab.

A final analysis will be performed once the last participant has completed the last visit.

Clinical Study Reports will be compiled following each analysis; a final Clinical Study Report will be produced using all available data.

16.1 Sample Size and Power Calculations

The sample size for this study is selected in order to establish a comprehensive safety database and to characterize the safety profile of VLA2001. 3,000 participants vaccinated with VLA2001 will allow for the detection of at least 1 rare event (incidence rate 1/1000) with a probability of 94% in this study. The VLA2001 safety database will then provide safety data on more than 3000 vaccinated participants.

The planned number of 600 participants per group in the immunogenicity subset will allow for a statistical power of 90% to detect superiority in terms of the Day 43 GMT ratio VLA2001/AZD1222 with an expected ratio of 1.3, a standard deviation of 0.6 (on a LOG10 scale), expected drop-out rate of 10% and a two-sided significance level of 5%.

All participants aged > 55 years will be analysed in this subset, whereas younger participants will be randomly selected for this subset.

For the adolescent part of the study a formal sample size calculation has not been performed as there will be no formal hypothesis testing. However, approximately 330 adolescents vaccinated with VLA2001 is considered to provide sufficient immunogenicity data to support immune-bridging to the adult population. For example, considering a two-sided significance level of 5% and power of 90%, a standard deviation of 0.60 (on a LOG10 scale) for neutralising antibody titres, no expected difference between groups and a drop-out rate of 10% the calculated sample size is approximately 300.

16.2 Analysis Populations:

Analysis Populations:

The **Safety population** contains all participants who entered into the study and received at least one study vaccination. Participants will be analyzed as treated.

The **Booster safety population** for adult and adolescent participants will contain all participants who received a booster dose.

The **Immunogenicity (IMM) population** is defined to include all randomized and vaccinated participants of the IMM subset for the primary endpoint evaluation, who were SARS-CoV-2 seronegative and have at least one evaluable post-baseline antibody titer measurement after vaccination. Participants who meet the case definition of confirmed COVID-19 during the study will not be included in the primary endpoint evaluation.

The **PBMC population** will be a randomized subset of the immunogenicity population (participants aged 30 years and above). 200 adult participants (100 adult participants of the

VLA2001 immunogenicity subset and 100 adult participants of the AZD1222 immunogenicity subset) will randomized for PBMC sample collection and analysis.

In addition, 100 adolescent participants (50 adolescent participants of the VLA2001 and 50 adolescent participants of the placebo group) will be randomized for PBMC sample collection and analysis.

The **Per Protocol (PP) population** contains all IMM population participants who have no major protocol violations that could impact the immune response.

The **Booster Immunogenicity population** will contain a subset of participants who received a booster dose with VLA2001 and from which blood samples are drawn. It is planned to include:
- approximately 330 adult participants who have received 2 doses of VLA2001 for primary immunization
- approximately 110 participants who had received 2 doses of AZD1222 for primary immunization and
- approximately 50 adolescent participants who have received 2 doses of VLA2001 for primary immunization

Selection of subjects for the immunogenicity blood sampling is described in section 10.5.1.

Analysis of T-cell response will be done for those participants from whom PBMC samples are available (see section 10.5.1).

16.3 Handling of Missing, Unused, and Spurious Data

All immunogenicity analysis will be based on observed values. Missing values will neither be replaced nor estimated. For missing data in AE evaluation (e.g. severity information) a worst-case approach will be applied.

16.4 Methods of Analysis

A Statistical Analysis Plan (SAP) will be provided describing in more detail, how the study results will be evaluated. The SAP will be finalized prior to the respective Blind Data Review Meetings.

Data will be summarized by treatment group and, where appropriate, by visit and SARS-CoV-2 baseline serological status. Descriptive statistics (number of observations, mean, standard deviation, minimum, median, and maximum) will be provided for continuous variables (e.g. age and weight). Frequency counts and percentages will be presented for categorical variables (e.g. gender).

All data exclusions, including premature terminations, will be detailed and tabulated. Data listings will include enrolled participants.

Baseline characteristics including demographic variables, medical and vaccination history and concomitant medications will be presented.

AEs and medical history will be coded using the MedDRA coding dictionary. Concomitant medications (including vaccinations) will be coded using the WHO Drug Dictionary.

More detailed criteria to identify participants in each analysis set, other research questions of interest not covered in this protocol, the definition of endpoints and details of their calculation, as well as further details on how to deal with missing, unused and spurious data will be covered in the Statistical Analysis Plan (SAP). If a change of the planned analyses is considered necessary after protocol finalization, this will be described and justified in the SAP. If a change is made after the statistical analysis has been performed, this will be described and justified in the CSR.

16.4.1 Immunogenicity Analysis

The immunogenicity assessments according to the defined endpoints will be determined and summarized by time point using appropriate descriptive statistics:

The primary immunogenicity endpoint (Day 43 GMT) will be compared between groups using a t-test applied to neutralization titres after LOG10 transformation. An analysis of variance with the factors study site and treatment group will be applied secondarily for sensitivity analysis.

The SARS-CoV-2 neutralising antibody titres, S-protein binding IgG level and cellular immune responses for the immunogenicity population (or PBMC subset) will be determined and summarized by time point using appropriate descriptive statistics in line with the respective endpoints. More details will be covered in the SAP.

16.4.2 Safety Analysis

Vital signs, clinical laboratory tests, and physical examination findings will be listed and summarized by time point using appropriate descriptive statistics.

The number and percentage of participants reporting any treatment-emergent adverse event (TEAE) or reactogenicity will be tabulated by system organ class and preferred term (coded using MedDRA).

TEAEs will be further classified by severity and relationship to treatment for up to Day 43 and for SAEs and AESIs up to Day 365. A first statistical analysis will be performed after all participants have been vaccinated and completed the visit 14 days after the second vaccination (i.e. Day 43).

16.5 Clinical Study Report

Clinical Study Reports (CSRs) will be compiled following each analysis; a final CSR will be produced using all available data.

17. ETHICS AND REGULATORY ASPECTS

17.1 Compliance Statement

This study will be conducted in accordance with this protocol, current ICH GCP guidelines, Declaration of Helsinki, and with the applicable national and local regulatory requirements.

17.2 Ethics Committee and Regulatory Authorities

Before enrolment of healthy volunteers into this study, the protocol, informed consent form, any promotional material/advertisements, and any other requested information will be reviewed and approved/given favourable opinion by the EC and applicable regulatory authorities in accordance with local requirements. The study will commence only upon the Sponsor's receipt of approval/favourable opinion from the EC.

If the protocol and/or any other information given to the participant is/are amended, the revised document(s) will be reviewed and approved/given favourable opinion by the EC and applicable regulatory authorities in accordance with local requirements, where applicable. The protocol amendment will only be implemented upon the Sponsor's receipt of approval. Amendments that are intended to eliminate an apparent immediate hazard to participants may be implemented prior to receiving EC and authority approval. However, in this case, approval must be obtained as soon as possible after implementation.

17.3 Participant Information and Informed Consent

It is the Investigator's responsibility to obtain freely given, written, informed consent from the participant before the participant is exposed to any study-related procedures, including screening tests for eligibility.

The informed consent form will include a comprehensive explanation of the proposed treatment without any exculpatory statements, in accordance with the elements required by ICH GCP and applicable regulatory requirements. Volunteers will be allowed sufficient time to consider participation in the study after having the nature and risks of the study explained to them. By signing the informed consent form, volunteers agree that all evaluations required by the study will be completed, unless they withdraw voluntarily or are terminated from the study for any reason.

The Investigator will explain that the participants are completely free to refuse to enter the study or to withdraw from it at any time, without any prejudice and need for justification. The participants will be informed that representatives of the Sponsor and health authority inspector may review their source records, and that these persons are bound by confidentiality obligations.

The participant will be given a copy or a second original of the ICF. An original of the signed and dated ICF must be retained in the site's records and is participant to inspection by representatives of the Sponsor or representatives from regulatory agencies.

The Sponsor will provide to the Investigator in written form any new information that significantly bears on the participants' risks associated with study vaccine exposure. The informed consent form will be updated, if necessary. This new information and/or revised informed consent form, which has been approved by the applicable EC and regulatory authorities, will be provided by the Investigator to the participants who consented to participate in the study.

18. QUALITY CONTROL AND QUALITY ASSURANCE

18.1 Source Data and Records

Source data are defined as all information related to clinical findings, observations or other activities in the study, captured in original records or certified copies of original records. The Investigator will permit study-related monitoring, audits, EC review and regulatory inspections, by providing direct access to source data/records. Source records should be preserved for the maximum period of time required by local regulations.

Source data entries must be made in accordance with local requirements. Signed and dated copies of the laboratory result reports have to be kept within the participant's source data file.

For data collected via the electronic participant diary (eDiary), the eDiary is regarded as source document.

18.2 Investigator's Responsibility

The Investigator will comply with the protocol (which has been approved/given favourable opinion by the EC), ICH GCP, and applicable regulatory requirements. The Investigator is ultimately responsible for the conduct of all aspects of the study at the study site and verifies by signature the integrity of all data transmitted to the Sponsor. The term 'Investigator', as used in this protocol and in study documents, refers to the Investigator or authorized study personnel whom the Investigator has designated to perform certain duties. Sub-Investigators or other authorized study personnel are eligible to sign for the Investigator, except where the Investigator's signature is specifically required.

18.3 Training

The study monitor will ensure that the Investigator and study site personnel understand all requirements of the protocol, the investigational status of the vaccine, and his/her regulatory responsibilities as an Investigator. Training may be provided at an Investigator's meeting, at the study site, web-based, and/or by instruction manuals. In addition, the study monitor will be available for consultation with the Investigator and will serve as the liaison between the study site and the Sponsor.

18.4 Monitoring

A designated monitor will check electronic system data and source data at regular intervals throughout the study to verify completeness, accuracy and consistency of the data, protocol adherence, and adherence to GCP guidelines. The monitor will work and perform Source Data Verification according to the Monitoring Plan. The Investigator will cooperate with the monitor to ensure that any discrepancies identified are resolved.

18.5 Audit and Inspection

Upon request, the Investigator will make all study-related source data and records available to a qualified quality assurance auditor mandated by the Sponsor or to regulatory inspectors. The main purposes of an audit or inspection are to confirm that the rights and welfare of the participants have been adequately protected, and that all data relevant for the assessment of safety and efficiency of the investigational product have appropriately been reported to the Sponsor.

18.6 Non-Compliance with the Protocol/Deviations from the Protocol

Any deviations from the protocol will be tracked, actions defined, as feasible, and reviewed in Data Review meetings for the study part analysis and the final analysis for assessment of their influence on the quality of the study analysis.

18.7 Confidentiality of Participant's Data

The Investigator will exercise all reasonable precautions within the constraints of the applicable regulatory requirements to maintain the confidentiality of participants' identities. On exported electronic source data or any other documents submitted to the Sponsor, participants will only be identified by participant number. Documents not for submission to the Sponsor, e.g. participant identification log and original ICF, will be maintained by the Investigator in strict confidence.

19. DATA HANDLING AND RECORD KEEPING

19.1 Information of Investigators

An Investigator Brochure (IB) containing all important data relating to the safe use of the IMP will be supplied to the Investigator prior to study start.

The Investigator will be kept informed on new relevant safety data as the study proceeds.

19.2 Electronic Case Report Forms

19.2.1 Data Recorded Directly on Case Report Forms

An eCRF will be used for this study. Data will be recorded directly onto source documents before documentation in the eCRF.

19.2.2 Electronic Case Report Form Entries

Electronic case report form entries and corrections will only be performed by study site staff authorized by the Investigator. Each user is informed of the clinical study's web-site internet address and is allocated to a user account with personal password to access the confidential website. The personal password must be kept confidential and must only be used by the person to whom it was assigned. For additional authorized users at the site, a new user account has to be requested to ensure that each entry/change can be allocated to the person who performed the entry/change.

All visit data need to be recorded in the eCRF database as soon as possible after each study visit.

19.2.3 Changes to Electronic Case Report Form Data

Corrections may be requested as follows:

- Investigators' responses are checked as they are entered and are rejected if they do not fulfil quality criteria. A message will specify the type of error or syntax error and assist in its correction.
- If required, the CRA can ask for information to be corrected during monitoring.
- Computerized data-check programs and manual checks will identify clinical data discrepancies for resolution. Corresponding queries will be created within the data capturing system and the site will be informed about new issues to be resolved on-line.

All discrepancies will be solved on-line directly by the Investigator or by authorized staff.

Corrections of eCRF data may be performed by authorized staff only. The person performing the changes in the eCRF is required to confirm electronically the changes made.

19.2.4 Electronic Case Report Form Entry Validation

The Investigator will thoroughly review the data on the eCRF and will finally certify the contents of the eCRF by electronic signature after completion of each participant. If a correction is made to the eCRF data after the Investigator's final approval, the certification must be repeated after the changes have been performed.

19.2.5 Data Collection

All visits and assessments are entered into an interactive form. Electronic case report forms will be source document verified following guidelines established before study onset and detailed in the Monitoring Plan. The study database will be maintained. Details of eCRF handling are provided in a study specific eCRF manual.

19.3 Coding of Adverse Events, Drugs and Diseases

After data entry, AEs and medical history will be coded according to the latest version of the Medical Dictionary for Regulatory Activities (MedDRA). The same version will be applied to all study parts. Previous and concomitant medication and vaccines will be coded according to the latest version of the WHO Drug Reference List and Anatomical Therapeutic Chemical Classification System.

19.4 Investigator File

19.4.1 Maintenance

The Investigator will maintain complete and accurate study documentation in a separate file (i.e. Investigator File) provided during the initiation visit. The Investigator is responsible for maintaining complete, up to date and accurate study records to enable the conduct of the study to be fully documented. The records should include the clinical protocol as well as any amendments, study approval letters, all original ICFs, drug dispensing and accountability logs, and all relevant correspondence pertaining to the study.

19.4.2 Archiving and destruction

All study-related documents should be kept by the Investigator for the maximum period of time required by local regulations. No study document should be destroyed without prior written agreement between the Investigator and the Sponsor. Should the Investigator elect to assign the study documents to another party, or move them to another location, the Sponsor must be notified.

19.4.3 Provision of Additional Information

On request, the Investigator will supply the Sponsor with additional data relating to the study or copies of relevant source records, duly anonymized. In case of particular issues or governmental queries, it is also necessary to have access to the complete study records, provided that the participant's confidentiality is protected in accordance with applicable regulations.

20. PUBLICATION POLICY

All results generated in this study will be considered to be strictly confidential. The Investigator may not submit the results for publication or presentation without prior written permission of the Sponsor. Authorship for any publication will be determined in mutual agreement. Within the scope of publication, co-authorship may be offered, at the sole discretion of the Sponsor, on a case-by-case basis taking scientific contribution into consideration. This is according to uniform requirements for manuscripts submitted to biomedical journals proposed by the International Committee of Medical Journal Editors.

21. LIABILITIES AND INSURANCE

In case of any damage or injury occurring to a participant in association with the participation in the study, insurance has been contracted.

The name, address and the insurance policy number will be given to both the Investigator prior to enrolment. Moreover, a copy of the insurance conditions will be filed on site.

The Investigator is responsible for dispensing the investigational product according to this protocol, and for its secure storage and safe handling throughout the study.

22. SUPPLEMENTS

22.1 Schedule of Study Procedures and Assessments (Adults)

The Investigator may schedule visits (unplanned visits in addition to those listed in the table), in order to conduct evaluations or assessments required to protect the well-being of the participant.

**Procedures/Assessments**	**Visit 0 Screening** (Day -7 to Day 1) [p]	**Visit 1** Day 1<br>**Baseline** [q]	**Visit 2** Day 8<br>(only for participants in the immunogenicity subgroup) (±1d)	**Visit 3** Day 29 (±2d) [q]	**Visit 4** Day 43 (±4d)	**Visit 5** Day 71 (±7d)	**Visit 6** Day 208 M7 (±14d)	**Visit 7** [t] M12 (±28d)<br>**End of Treatment**<br>**OR**<br>**Early Termination Visit**	**COVID-19 Verification Visit** [l]	**COVID-19 Illness Visit**	**Ad hoc Safety Call**<br>in case of (Grade 3 AE eDiary entry or SAE)	**Unscheduled Visit** [o]
Informed consent [a]	X											
Inclusion/Exclusion criteria check	X	X (Review)		X (Review)								
Vaccination Delay criteria check		X		X								
Demographics [b]	X											
Medical history [c] (including vaccination history)	X	X (Update)										
Prior/Concomitant medications	X	X	X	X	X	X	X	X		X	X	X
Physical/Symptom-driven examination [d]	X	X	X	X	X	X	X	X		X		X
Vital signs [e]	X	X		X								X
Safety Lab [f]	X		X [r]									X
Urine Pregnancy test [g]		X		X								
SARS-CoV-2 Antibody test [h]	X											
SARS-CoV-2 rapid Antigen test [i]		X										
Randomisation		X										
**VACCINATION** [q]		**X**		**X**								

Procedures/Assessments	Visit 0 Screening (Day -7 to Day 1) [p]	Visit 1 Day 1 Baseline [q]	Visit 2 Day 8 (only for participants in the immunogenicity subgroup) (±1d)	Visit 3 Day 29 (±2d) [q]	Visit 4 Day 43 (±4d)	Visit 5 Day 71 (±7d)	Visit 6 Day 208 M7 (±14d)	Visit 7 [t] M12 (±28d) End of Treatment OR Early Termination Visit	COVID-19 Verification Visit [l]	COVID-19 Illness Visit	Ad hoc Safety Call in case of (Grade 3 AE eDiary entry or SAE)	Unscheduled Visit [o]
Immunogenicity sample [j] [20 ml]		X	X [s]	X	X	X	X	X[t]		X		
Blood for PBMC Isolation [k] [50 mL]		X		X	X	X	X	X[t]				
SARS-CoV-2 (RT-PCR)									X			
SARS-CoV-2 strain identification [m]										X		
Electronic Participant Diary		E	R	R/E	R							
AE/AESI/SAE Assessment		X	X	X	X	X	X	X		X	X	X

AE=adverse event; AESI=adverse event of special interest; aPTT=activated partial thromboplastin time; BMI=body mass index; CRP=C-reactive protein; ELISA=enzyme-linked immunosorbent assay; ET=early termination; PBMC=peripheral blood mononuclear cells; PCR=polymerase chain reaction; R=review; SAE=serious adverse event

[a] Occurs at enrolment before Screening.

[b] Demographics include year of birth, height, weight, BMI, gender and ethnicity.

[c] Symptoms noted at Visit 1 (prior to first vaccination) are not considered AEs but will be recorded as medical history.

[d] At the screening visit, a **physical examination** will be performed on the following body systems being described as normal or abnormal: general appearance, head and neck, eyes and ears, nose and throat, chest, lungs, heart, abdomen, extremities and joints, lymph nodes, skin, and neurological. At all other study visits, a symptom-driven physical examination will be performed if the participant reports a symptom. In addition, at visits, where a vaccination is given, a symptom-driven physical examination shall be performed before the vaccination is given. Another symptom-driven examination may be necessary if the participant has complaints/symptoms within the observation time after vaccination including an assessment of the affected body system(s).

[e] **Vital signs** will include systolic and diastolic blood pressure and pulse rate while seated and at rest, and body temperature measured orally before vaccination. In addition, after an observation period of 30 minutes following vaccination (60 minutes for the first 10 participants aged 55+), pulse rate as well as blood pressure while seated and at rest will again be assessed.

[f] Safety labs assessment includes: blood sample for standard clinical chemistry (i.e. creatinine, sodium, potassium, calcium, aspartate aminotransferase, alanine aminotransferase, alkaline phosphatase, bilirubin, CRP), haematology (i.e. haemoglobin, haematocrit, erythrocyte count, white blood count, differential white blood cell count, platelets, erythrocyte sedimentation rate , coagulation panel (i.e. prothrombin time, aPTT, fibrinogen) [EDTA blood: 30 mL] and urinalysis (i.e. pH, specific gravity, leucocytes, nitrite, protein, glucose, ketones, urobilinogen, bilirubin, erythrocytes.

[g] A urine **pregnancy** test will be done prior to vaccination at Visit 1 and Visit 3.

[h] A rapid antibody test will be performed to check if a participant has previously been exposed and is seropositive or seronegative for SARS-CoV-2 virus.

[i] A rapid antigen test will be performed at baseline before randomization and vaccination to check if a participant is currently infected with SARS-CoV-2 virus. If the rapid antigen test is positive, a confirmatory RT-PCR test will be performed. If the RT-PCR test is positive, the participant will be excluded from the study in line with exclusion criteria 3.

i An **immunogenicity sample** will be drawn [20mL] for all participants. Just for participants in the immunogenicity population serum samples will be used for SARS-CoV-2-specific neutralising antibody titre evaluation of the primary endpoint, The samples will also be used for anti S Protein-ELISA and may be used for development or performance of further assays related to development of this vaccine candidate. For retrospective safety analysis if deemed necessary by the Data Safety Monitoring Board upon clinical indication or on demand if requested later by regulatory authorities.

[k] A blood sample [approx. 50mL] will be collected to isolate PBMCs from a subset of participants in the immunogenicity population for future investigation of cellular immune responses to VLA2001. PBMCs may also be used for further research activities considered relevant to SARS-CoV-2 virus.

[l] In case of an initial negative PCR test result, participants will be invited for a second confirmatory PCR test on site (i.e. COVID-19 verification visit see section 22.1) after approximately 2 days. In the event the result is still negative, the participant will continue with scheduled visits.

[m] Either a nasal swab or gargle (saliva) sample will be collected for genetic sequencing and identification of SARS-CoV-2 virus strain.

[o] The Investigator should decide on which tests or assessments to perform during an unscheduled visit depending on the reason for the visit.

[p] Visit 0 and Visit 1 may happen on the same day.

[q] Before vaccination, all applicable procedures of the day such as physical examinations, laboratory sample collections, should be done.

[r] Haematology assessments only

[s] Only for participants of the immunogenicity subset who are above 55 years of age

[t] For participants not eligible to receiving a booster or who do not wish to receive a booster dose with VLA2001 visit 7 will be done without any immunogenicity blood sample nor PBMC collection.

22.1 Schedule of Study Procedures and Assessments (Adults – Booster Phase)

The Investigator may schedule visits (unplanned visits in addition to those listed in the table), in order to conduct evaluations or assessments required to protect the well-being of the participant.

	Adults who receive 1 booster vaccination with VLA2001			For all participants			
**Procedures/Assessments**	**Visit B1** [q]<br>Booster Vacc. with VLA2001	**Visit B2**<br>Visit B1 +14 days (±2d)	**Visit B3**<br>Visit B1+180 days (±14d)<br>**End of Treatment**<br>**OR**<br>**Early Termination Visit**	**COVID-19 Verification Visit** [l]	**COVID-19 Illness Visit**	**Ad hoc Safety Call**	**Unscheduled Visit** [o]
Informed consent for Booster	X						
**VACCINATION** [q]	**X**						
Inclusion/Exclusion criteria check	X (Review)						
Vaccination Delay criteria check	X						
Prior/Concomitant medications	X	X	X		X	X	X
Physical/Symptom-driven examination [d]	X	X	X		X		X
Vital signs [e]	X	X	X				X
Safety Lab [f] [30 ml]	X						X
Urine Pregnancy test [g]	X						
Immunogenicity sample [j] [20 ml]	X	X	X		X		

	Adults who receive 1 booster vaccination with VLA2001			For all participants			
**Procedures/Assessments**	**Visit B1** [q]<br>Booster Vacc. with VLA2001	**Visit B2**<br>Visit B1 +14 days (±2d)	**Visit B3**<br>Visit B1+180 days (±14d)<br>**End of Treatment**<br>**OR**<br>**Early Termination Visit**	**COVID-19 Verification Visit** [l]	**COVID-19 Illness Visit**	**Ad hoc Safety Call**	**Unscheduled Visit** [o]
Blood for PBMC Isolation [k] [50 mL]	X	X	X				
SARS-CoV-2 (RT-PCR)				X			
SARS-CoV-2 strain identification [m]					X		
Electronic Participant Diary	E	R					
AE/AESI/SAE Assessment	X	X	X		X	X	X

AE=adverse event; AESI=adverse event of special interest; aPTT=activated partial thromboplastin time; BMI=body mass index; CRP=C-reactive protein; ELISA=enzyme-linked immunosorbent assay; ET=early termination; PBMC=peripheral blood mononuclear cells; PCR=polymerase chain reaction; E = explain; R=review; SAE=serious adverse event

[d] At all indicated visits and prior to discharge after vaccination at B1, B2 and B3 a **symptom-driven physical examination** will be performed, i.e. only in case symptom is reported by the participant, an assessment of the affected body system(s) will be performed.

[e] **Vital signs** will include systolic and diastolic blood pressure and pulse rate while seated and at rest, and body temperature measured orally before vaccination. In addition, after an observation period of 30 minutes following vaccination, pulse rate as well as blood pressure while seated and at rest will again be assessed.

[f] Safety labs assessment includes: blood sample for standard clinical chemistry (i.e. creatinine, sodium, potassium, calcium, aspartate aminotransferase, alanine aminotransferase, alkaline phosphatase, bilirubin, CRP), haematology (i.e. haemoglobin, haematocrit, erythrocyte count, white blood count, differential white blood cell count, platelets, erythrocyte sedimentation rate , coagulation panel (i.e. prothrombin time, aPTT, fibrinogen) [EDTA blood: 30 mL] and urinalysis (i.e. pH, specific gravity, leucocytes, nitrite, protein, glucose, ketones, urobilinogen, bilirubin, erythrocytes.

[g] A urine **pregnancy** test will be done prior to vaccination at Visit B1(WOCBP only).

[j] Only for participants of the Booster Immunogenicity population, an **immunogenicity sample** will be drawn [20mL] for all adult participants, who are boostered. The samples will be used for anti S-Protein-ELISA and may be used for development or performance of further assays related to development of this vaccine candidate. For retrospective safety analysis if deemed necessary by the Data Safety Monitoring Board upon clinical indication or on demand if requested later by regulatory authorities.

[k] Only for participants of the Booster Immunogenicity population, a blood sample [approx. 50mL] will be collected to isolate PBMCs from a subset of adult participants receiving a booster dose for future investigation of cellular immune responses. PBMCs may also be used for further research activities considered relevant to SARS-CoV-2 virus.

[l] In case of an initial negative PCR test result, participants will be invited for a second confirmatory PCR test on site (i.e. COVID-19 verification visit see section 22.1) after approximately 2 days. In the event the result is still negative, the participant will continue with scheduled visits.

[m] Either a nasal swab or gargle (saliva) sample will be collected for genetic sequencing and identification of SARS-CoV-2 virus strain.

[o] The Investigator should decide on which tests or assessments to perform during an unscheduled visit depending on the reason for the visit.

[q] Before vaccination, all applicable procedures of the day such as physical examinations, laboratory sample collections, should be done.

22.2 Schedule of Study Procedures and Assessments (Adolescents – Blinded part)

The Investigator may schedule visits (unplanned visits in addition to those listed in the table), in order to conduct evaluations or assessments required to protect the well-being of the participant.

**Procedures/Assessments**	**Visit V0a Screening** (Day -7 to Day 1) [o]	**Visit V1a** Day 1 **Baseline** [p]	**Visit V2a** Day 29 (±2d) [p]	**Visit V3a** Day 43 (±4d)	**COVID-19 Verification Visit** [l]	**COVID-19 Illness Visit**	**Ad hoc Safety Call**	**Unscheduled Visit** [n]
Informed assent and guardian consent [a]	X							
Inclusion/Exclusion criteria check	X	X (Review)	X (Review)					
Vaccination Delay criteria check		X	X					
Demographics [b]	X							
Medical history [c] (including vaccination history)	X	X (Update)						
Prior/Concomitant medications	X	X	X	X		X	X	X
Physical/Symptom-driven examination [d]	X	X	X	X		X		X
Vital signs [e]	X	X	X					X
Safety Lab [f] [30 ml]	X							X
Urine Pregnancy test [g]		X	X					
SARS-CoV-2 Antibody test [h]	X							
SARS-CoV-2 rapid Antigen test [i]		X						
Randomisation		X						
Unblinding								
**VACCINATION** [p]		**X**	**X**					

Procedures/Assessments	**Visit V0a Screening** (Day -7 to Day 1) [o]	**Visit V1a** Day 1 **Baseline** [p]	**Visit V2a** Day 29 (±2d) [p]	**Visit V3a** Day 43 (±4d)	**COVID-19 Verification Visit** [l]	**COVID-19 Illness Visit**	**Ad hoc Safety Call**	**Unscheduled Visit** [n]
Immunogenicity sample [j] [10 ml]		X	X [j]	X		X		
Blood for PBMC Isolation [k] [30 mL]		X		X				
SARS-CoV-2 (RT-PCR)					X			
SARS-CoV-2 strain identification [m]						X		
Electronic Participant Diary		X	X					
AE/AESI/SAE Assessment		X	X	X		X	X	X

AE=adverse event; AESI=adverse event of special interest; aPTT=activated partial thromboplastin time; BMI=body mass index; CRP=C-reactive protein; ELISA=enzyme-linked immunosorbent assay; ET=early termination; PBMC=peripheral blood mononuclear cells; PCR=polymerase chain reaction; E = explain; R=review; SAE=serious adverse event

[a] Occurs at enrolment before Screening.

[b] Demographics include year of birth, height, weight, BMI, gender and ethnicity.

[c] Symptoms noted at Visit V1a (prior to first vaccination) are not considered AEs but will be recorded as medical history.

[d] At the screening visit, a **physical examination** will be performed on the following body systems being described as normal or abnormal: general appearance, head and neck, eyes and ears, nose and throat, chest, lungs, heart, abdomen, extremities and joints, lymph nodes, skin, and neurological. At all other study visits, a symptom-driven physical examination will be performed if the participant reports a symptom. In addition, at visits, where a vaccination is given, a symptom-driven physical examination shall be performed before the vaccination is given. Another symptom-driven examination may be necessary if the participant has complaints/symptoms within the observation time after vaccination including an assessment of the affected body system(s).

[e] **Vital signs** will include systolic and diastolic blood pressure and pulse rate while seated and at rest, and body temperature measured orally before vaccination. In addition, after an observation period of 30 minutes following vaccination, pulse rate as well as blood pressure while seated and at rest will again be assessed.

[f] Safety labs assessment includes: blood sample for standard clinical chemistry (i.e. creatinine, sodium, potassium, calcium, aspartate aminotransferase, alanine aminotransferase, alkaline phosphatase, bilirubin, CRP), haematology (i.e. haemoglobin, haematocrit, erythrocyte count, white blood count, differential white blood cell count, platelets, erythrocyte sedimentation rate, coagulation panel (i.e. prothrombin time, aPTT, fibrinogen) [EDTA blood: 30 mL] and urinalysis (i.e. pH, specific gravity, leucocytes, nitrite, protein, glucose, ketones, urobilinogen, bilirubin, erythrocytes.

[g] A urine **pregnancy** test will be done prior to vaccination at Visits V1a, V2a (WOCBP only).

[h] A rapid antibody test will be performed to check if a participant has previously been exposed and is seropositive or seronegative for SARS-CoV-2 virus.

[i] A rapid antigen test will be performed at baseline before randomization and vaccination to check if a participant is currently infected with SARS-CoV-2 virus. If the rapid antigen test is positive, a confirmatory RT-PCR test will be performed. If the RT-PCR test is positive, the participant will be excluded from the study in line with exclusion criteria 3.

[j] An **immunogenicity sample** will be drawn [10mL] for all adolescent participants. The samples will also be used for anti S-Protein-ELISA and may be used for development or performance of further assays related to development of this vaccine candidate. For retrospective safety analysis if deemed necessary by the Data Safety Monitoring Board upon clinical indication or on demand if requested later by regulatory authorities.

[k] A blood sample [approx. 30mL] will be collected to isolate PBMCs from a subset of adolescent participants for future investigation of cellular immune responses. PBMCs may also be used for further research activities considered relevant to SARS-CoV-2 virus.

[l] In case of an initial negative PCR test result, participants will be invited for a second confirmatory PCR test on site (i.e. COVID-19 verification visit see section 22.1) after approximately 2 days. In the event the result is still negative, the participant will continue with scheduled visits.

[m] Either a nasal swab or gargle (saliva) sample will be collected for genetic sequencing and identification of SARS-CoV-2 virus strain.

[n] The Investigator should decide on which tests or assessments to perform during an unscheduled visit depending on the reason for the visit.

[o] Visit V0a and Visit V1a may happen on the same day.

[p] Before vaccination, all applicable procedures of the day such as physical examinations, laboratory sample collections, should be done.

22.3 Schedule of Study Procedures and Assessments (Adolescents – Unblinded part)

The Investigator may schedule visits (unplanned visits in addition to those listed in the table), in order to conduct evaluations or assessments required to protect the well-being of the participant.

	Adolescent who received placebo will now receive 2 vaccinations with VLA2001					Adolescent who received VLA2001 will now receive 1 booster vaccination with VLA2001				For all participants			
**Procedures/Assessments**	**Visit 4p**<br>Day 71 (±7d) [a]<br>1st Vacc. with VLA2001	**Visit 5p**<br>V4p + 28 days (±2d) [a]<br>2nd Vacc. with VLA2001	**Visit 6p**<br>V5p + 14 days (±2d)	**Visit 7p** [j]<br>V5p + 208 days (±7d)	**Visit 8p**<br>M12 (±14d)<br>**End of Treatment**<br>**OR**<br>**Early Termination Visit**	**Visit V4ab**<br>Day 71 (±7d)	**Visit V5ab**<br>Day 208 (±7d) [a]<br>Booster Vacc. with VLA2001	**Visit V6ab**<br>V5ab + 14 days (±2d)	**Visit V7ab**<br>M12 (±14d)<br>**End of Treatment**<br>**OR**<br>**Early Termination Visit**	**COVID-19 Verification Visit** [k]	**COVID-19 Illness Visit**	**Ad hoc Safety Call**	**Unscheduled Visit** [l]
**Unblinding**	**X**					**X**							
**VACCINATION** [a]	**X**	**X**					**X**						
Inclusion/Exclusion criteria check	X (Review)	X (Review)					X (Review)						
Vaccination Delay criteria check	X	X					X						
Prior/Concomitant medications	X	X	X	X	X	X	X	X	X		X	X	X
Physical/Symptom-driven examination [b]	X	X	X	X	X	X	X	X	X		X		X
Vital signs [c]	X	X					X						X
Safety Lab [d] [30 ml]													X
Urine Pregnancy test [e]	X	X					X						

	Adolescent who received placebo will now receive 2 vaccinations with VLA2001					Adolescent who received VLA2001 will now receive 1 booster vaccination with VLA2001				For all participants			
**Procedures/Assessments**	**Visit 4p**<br>Day 71<br>(±7d) [a]<br>1st Vacc. with VLA2001	**Visit 5p**<br>V4p + 28 days<br>(±2d) [a]<br>2nd Vacc. with VLA2001	**Visit 6p**<br>V5p + 14 days<br>(±2d)	**Visit 7p** [j]<br>V5p + 208 days<br>(±7d)	**Visit 8p**<br>M12<br>(±14d)<br>**End of Treatment**<br>**OR**<br>**Early Termination Visit**	**Visit V4ab**<br>Day 71<br>(±7d)	**Visit V5ab**<br>Day 208<br>(±7d) [a]<br>Booster Vacc. with VLA2001	**Visit V6ab**<br>V5ab + 14 days<br>(±2d)	**Visit V7ab**<br>M12<br>(±14d)<br>**End of Treatment**<br>**OR**<br>**Early Termination Visit**	**COVID-19 Verification Visit** [k]	**COVID-19 Illness Visit**	**Ad hoc Safety Call**	**Unscheduled Visit** [l]
Immunogenicity sample [f] [10 ml]	X	X	X		X	X	X	X	X		X		
Blood for PBMC Isolation [g] [30 mL]	X	X	X		X	X	X	X	X				
SARS-CoV-2 (RT-PCR)										X			
SARS-CoV-2 strain identification [h]											X		
Electronic Participant Diary	X	X					X						
AE/AESI/SAE Assessment [i]	X	X	X	X	X	X	X	X	X		X	X	X

AE=adverse event; AESI=adverse event of special interest; aPTT=activated partial thromboplastin time; BMI=body mass index; CRP=C-reactive protein; ELISA=enzyme-linked immunosorbent assay; ET=early termination; PBMC=peripheral blood mononuclear cells; PCR=polymerase chain reaction; E = explain; R=review; SAE=serious adverse event

[a] Before vaccination, all applicable procedures of the day such as physical examinations, laboratory sample collections, should be done.

[b] At all indicated study visits, a symptom-driven physical examination will be performed if the participant reports a symptom. In addition, at visits, where a vaccination is given, a symptom-driven physical examination shall be performed before the vaccination is given. Another symptom-driven examination may be necessary if the participant has complaints/symptoms within the observation time after vaccination including an assessment of the affected body system(s).

[c] **Vital signs** will include systolic and diastolic blood pressure and pulse rate while seated and at rest, and body temperature measured orally before vaccination. In addition, after an observation period of 30 minutes following vaccination, pulse rate as well as blood pressure while seated and at rest will again be assessed.

[d] Safety labs assessment includes: blood sample for standard clinical chemistry (i.e. creatinine, sodium, potassium, calcium, aspartate aminotransferase, alanine aminotransferase, alkaline phosphatase, bilirubin, CRP), haematology (i.e. haemoglobin, haematocrit, erythrocyte count, white blood count, differential white blood cell count, platelets, erythrocyte sedimentation rate , coagulation panel (i.e. prothrombin time, aPTT, fibrinogen) [EDTA blood: 30 mL] and urinalysis (i.e. pH, specific gravity, leucocytes, nitrite, protein, glucose, ketones, urobilinogen, bilirubin, erythrocytes.

[e] A urine **pregnancy** test will be done prior to vaccination at Visits V4p, V5p and V5ab (WOCBP only).

[f] An **immunogenicity sample** will be drawn [10mL] for all adolescent participants. The samples will also be used for anti S-Protein-ELISA and may be used for development or performance of further assays related to development of this vaccine candidate. For retrospective safety analysis if deemed necessary by the Data Safety Monitoring Board upon clinical indication or on demand if requested later by regulatory authorities.

[g] A blood sample [approx. 30mL] will be collected to isolate PBMCs from a subset of adolescent participants for future investigation of cellular immune responses. PBMCs may also be used for further research activities considered relevant to SARS-CoV-2 virus.

[h] Either a nasal swab or gargle (saliva) sample will be collected for genetic sequencing and identification of SARS-CoV-2 virus strain.

[i] AE, AESIs and SAEs will be reported in the eCRF up till the end of study.

[j] Visit may be performed remotely (i.e. over the phone) if no symptoms are present.

[k] In case of an initial negative PCR test result, participants will be invited for a second confirmatory PCR test on site (i.e. COVID-19 verification visit see section 22.1) after approximately 2 days. In the event the result is still negative, the participant will continue with scheduled visits.

[l] The Investigator should decide on which tests or assessments to perform during an unscheduled visit depending on the reason for the visit.

22.4 Toxicity Grading Scale for Abnormal Laboratory Assessments

	**Mild (Grade 1)** [a]	**Moderate (Grade 2)**	**Severe (Grade 3)**	**Potentially life threatening (Grade 4)** [b,e]
Haematology Parameters				
Haemoglobin (Female) – gm/dL	11.0–12.0	9.5–10.9	8.0–9.4	<8.0
Haemoglobin (Male) – gm/dL	12.5–13.5	10.5–12.4	8.5–10.4	<8.5
Haematocrit	Outside normal range [c]			
Erythrocyte count	Outside normal range [c]			
WBC Increase – cell/mm^3	10,800–15,000	15,001–20,000	20,001–25,000	>25,000
WBC Decrease – cell/mm^3	2,500–3,500 [d]	1,500–2,499	1,000–1,499	<1,000
Neutrophils Decrease – cell/mm^3	1,500–2,000	1,000–1,499	500–999	<500
Lymphocytes Decrease – cell/mm^3	750–1,000	500–749	250–499	<250
Monocytes	Outside normal range [c]			
Eosinophils – cell/mm^3	650–1500[4]	1501–5000	>5000	Hyper-eosinophilic
Basophils	Outside normal range [c]			
Platelets Decreased – cell/mm^3	125,000 – 140,000 [d]	100,000 – 124,000	25,000–99,000	<25,000
ESR	Outside normal range [c]			
Clinical Chemistry Parameters				
Creatinine – mg/dL	1.5–1.7 [d]	1.8–2.0	2.1–2.5	>2.5 or requires dialysis
Sodium – Hyponatremia mEq/L	132–134	130–131	125–129	<125
Sodium – Hypernatremia mEq/L	144–145 [d]	146–147	148–150	>150

	**Mild (Grade 1) [a]**	**Moderate (Grade 2)**	**Severe (Grade 3)**	**Potentially life threatening (Grade 4) [b,e]**
Potassium – Hyperkalaemia mEq/L	5.1–5.2 [d]	5.3–5.4	5.5–5.6	>6
Potassium – Hypokalaemia mEq/L	3.5–3.6 [d]	3.3–3.4	3.1–3.2	<3.1
Calcium – Hypocalcaemia mg/dL	8.0–8.4 [d]	7.5–7.9	7.0–7.4	<7.0
Calcium – Hypercalcaemia mg/dL	10.5–11.0	11.1–11.5	11.6–12.0	>12.0
**AST** – increase by factor	1.1–2.5 x ULN	2.6–5.0 x ULN	5.1–10 x ULN	>10 x ULN
**ALT** – increase by factor	1.1–2.5 x ULN	2.6–5.0 x ULN	5.1–10 x ULN	>10 x ULN
Alkaline phosphatase – increase by factor	1.1–2.0 x ULN	2.1–3.0 x ULN	3.1– 10 x ULN	>10 x ULN
**Bilirubin** – when accompanied by any increase in Liver Function Test increase by factor	1.1–1.25 x ULN	1.26–1.5 x ULN	1.51–1.75 x ULN	>1.75 x ULN
**Bilirubin** – when Liver Function Test is normal; increase by factor	1.1–1.5 x ULN	1.6–2.0 x ULN	2.0–3.0 x ULN	>3.0 x ULN
CRP	Outside normal range [c]			
Coagulation Factors				
**PT** – increase by factor	1.0–1.10 x ULN	1.11–1.20 x ULN	1.21–1.25 x ULN	>1.25 ULN
**PTT (aPTT)** – increase by factor	1.0–1.2 x ULN	1.21–1.4 x ULN	1.41–1.5 x ULN	>1.5 x ULN
Fibrinogen increase - mg/dL	400–500 [d]	501–600	>600	–
Fibrinogen decrease - mg/dL	150–200 [d]	125–149	100–124	<100 or associated with gross bleeding or disseminated

	**Mild (Grade 1)** [a]	**Moderate (Grade 2)**	**Severe (Grade 3)**	**Potentially life threatening (Grade 4)** [b,e]
				intravascular coagulation

AE=adverse event; ALT=alanine aminotransferase; AST=aspartate aminotransferase; aPTT=activated partial thromboplastin time; CRP=C reactive protein; ESR=erythrocyte sedimentation rate; FDA=Food and Drug Administration; PT=prothrombin time; SAE=serious adverse event; ULN=upper limit of normal; WBC=white blood cell

[a] In case the laboratory's normal ranges and absolute Grade 1 limits overlap, Grade 1 limits will prevail, i.e. the value will be classified as Grade 1 abnormality even if it is within central laboratory normal ranges. Values between the central laboratory normal ranges and absolute Grade 1 limits will be reported as no abnormality (Grade 0).

[b] The clinical signs or symptoms associated with laboratory abnormalities might result in characterization of the laboratory abnormalities as potentially life threatening (Grade 4). For example, a low sodium value that falls within a grade 3 parameter (125–129 mE/L) should be recorded as a grade 4 hyponatremia unsolicited AE if the participant had a new seizure associated with the low sodium value.

[c] As neither the FDA Scale nor the Division of AIDS Table for Grading the Severity of Adult and Pediatric Adverse Events (December 2004) provide any grading for haematocrit, erythrocyte count, monocytes, Basophils, ESR and CRP, these will only be analysed as 'outside normal range', as determined by central laboratory standards and graded as described in Section 15.2.2 upon Investigator's judgement.

[d] Central laboratory values should be adjusted to FDA toxicity grading scale. Specifically, if central laboratory reference range is more stringent than FDA toxicity grading scale the central laboratory values should be reported as no abnormality (Grade 0). Similarly, if laboratory values are within the central laboratory normal reference range, but fall into FDA toxicity grading scale, the values should be reported as indicated by the FDA toxicity grading scale.

[e] Any Grade 4 abnormal laboratory value should be reported as an SAE (see Section 16.1.2).

22.5 Adverse Events of Special Interest

Neuroinflammatory Disorders	Acute disseminated encephalomyelitis (including site specific variants: e.g., non-infectious encephalitis, encephalomyelitis, myelitis, myeloradiculomyelitis), anosmia, ageusia, cranial nerve disorders including paralyses/paresis (e.g., Bell's palsy), generalized convulsion, Guillain-Barre syndrome (including Miller Fisher syndrome and other variants), immune-mediated peripheral neuropathies and plexopathies (including chronic inflammatory demyelinating polyneuropathy, multifocal motor neuropathy and polyneuropathies associated with monoclonal gammopathy), meningoencephalitis, myasthenia gravis, multiple sclerosis, narcolepsy, optic neuritis, transverse myelitis, uveitis
Musculoskeletal and Connective Tissue Disorders	Antisynthetase syndrome, dermatomyositis, juvenile chronic arthritis (including Still's disease), mixed connective tissue disorder, polymyalgia rheumatic, polymyositis, psoriatic arthropathy, relapsing polychondritis, rheumatoid arthritis, scleroderma (including diffuse systemic form and CREST syndrome), spondyloarthritis (including ankylosing spondylitis, reactive arthritis [Reiter's Syndrome] and undifferentiated spondyloarthritis), systemic lupus erythematosus, systemic sclerosis, Sjogren's syndrome
Vasculitides	Large vessels vasculitis (including giant cell arteritis such as Takayasu's arteritis and temporal arteritis), medium sized and/or small vessels vasculitis (including polyarteritis nodosa, Kawasaki's disease, microscopic polyangiitis, Wegener's granulomatosis, Churg–Strauss syndrome [allergic granulomatous angiitis], Buerger's disease [thromboangiitis obliterans], necrotizing vasculitis and anti-neutrophil cytoplasmic antibody [ANCA] positive vasculitis [type unspecified], Henoch-Schonlein purpura, Behcet's syndrome, leukocytoclastic vasculitis)
Gastrointestinal Disorders	Crohn's disease, celiac disease, liver injury, ulcerative colitis, ulcerative proctitis
Hepatic Disorders	Autoimmune hepatitis, autoimmune cholangitis, primary sclerosing cholangitis, primary biliary cirrhosis
Renal Disorders	Acute kidney injury, autoimmune glomerulonephritis (including IgA nephropathy, glomerulonephritis rapidly progressive, membranous glomerulonephritis, membranoproliferative glomerulonephritis, and mesangioproliferative glomerulonephritis,
Cardiac Disorders	Autoimmune myocarditis/cardiomyopathy<br><br>Acute cardiac injury including:<br>• Microangiopathy<br>• Heart failure and cardiogenic shock<br>• Stress cardiomyopathy<br>• Coronary artery disease

	• Arrhythmia<br>• Myocarditis, pericarditis
Skin Disorders	Alopecia areata, psoriasis, vitiligo, Raynaud's phenomenon, erythema nodosum, autoimmune bullous skin diseases (including pemphigus, pemphigoid and dermatitis herpetiformis), cutaneous lupus erythematosus, morphoea, lichen planus, Stevens-Johnson syndrome, Sweet's syndrome
Haematologic Disorders	Thrombosis with thrombocytopenia<br>Autoimmune haemolytic anaemia, autoimmune thrombocytopenia, antiphospholipid syndrome, thrombocytopenia<br>Coagulation disorder<br>• Deep vein thrombosis<br>• Pulmonary embolus<br>• Cerebrovascular stroke<br>• Limb ischemia<br>• Haemorrhagic disease
Metabolic Disorders	Autoimmune thyroiditis, Grave's or Basedow's disease, Hashimoto´s thyroiditis, diabetes mellitus type 1, Addison's disease
Other Disorders	Goodpasture syndrome, idiopathic pulmonary fibrosis, pernicious anaemia, sarcoidosis
Immunologic	Enhanced disease following immunization, cytokine release syndrome related to COVID-19 disease
Respiratory	Acute respiratory distress syndrome (ARDS)
Dermatologic	Chilblain-like lesions, single organ cutaneous vasculitis, erythema multiforme

In addition, for adolescents, the occurrence of PIMS-TS/ MIS-C and vaccine enhanced disease, is considered as a AESI given this is an inactivated adjuvanted vaccine.

23. REFERENCES

Arabi YM, Alothman A, Balkhy HH et al. Treatment of Middle East Respiratory Syndrome with a combination of lopinavir-ritonavir and interferon-beta1b (MIRACLE trial): study protocol for a randomized controlled trial. Trials 2018;19:81

Arabi YM, Mandourah Y, Al-Hameed F et al. Corticosteroid therapy for critically Ill patients with Middle East respiratory syndrome. Am J Respir Crit Care Med. 2020;197:757–767

Astuti I, Ysrafil. Severe Acute Respiratory Syndrome Coronavirus 2 (SARS-CoV-2): An overview of viral structure and host response. Diabetes Metab Syndr 2020;14(4):407-412

Chaolin H, Yeming W, Xingwang L. Clinical features of patients infected with 2019 novel coronavirus in Wuhan, China. Lance. 2020;(395):497–506

Gao Q, Bao L, Mao H et al. Development of an inactivated vaccine candidate for SARS-CoV-2. Science 369(6499):77–81

Guan W, Ni Z, Hu Y et al. Clinical Characteristics of Coronavirus Disease 2019 in China. N Eng J Med 2020;382:1708–1720

HEPLISAV-B Summary of product characteristics https://rsc.niaid.nih.gov/sites/default/files/1-14-2-2-package-insert-heplisav-b.pdf (Accessed 28 Aug 2020)

IXIARO Clinical Review Memorandum, 2018 https://www.fda.gov/files/vaccines,%20blood%20&%20biologics/published/Clinical-Review--IXIARO.pdf (accessed 29 Aug 2020)

John Hopkins Resource Center https://coronavirus.jhu.edu/ (accessed 28 Aug 2020)

Li Q, Guan X, Wu P et al. Early transmission dynamics in Wuhan, China, of novel coronavirus-infected pneumonia. N Engl J Med 2020;382(13): 1199–1207

Joshua G. Liang, Danmei Su, Tian-Zhang Song et al. S-Trimer, a COVID-19 subunit vaccine candidate, induces protective immunity in nonhuman primates https://doi.org/10.1101/2020.09.24.311027

Munoz FM, Cramer JP, Dekker CL et al. Vaccine-associated Enhanced Disease: Case Definition and Guidelines for Data Collection, Analysis, and Presentation of Immunization Safety Data. Submitted.

Sinovac Press Release from 13.06.2020; Sinovac Announces Positive Preliminary Results of Phase I/II Clinical Trials for Inactivated Vaccine Candidate Against COVID-19; http://www.sinovac.com/?optionid=754&auto_id=904 (accessed 28 Aug 2020)

Stefanelli P., Faggioni G., Lo Presti A et al. Whole genome and phylogenetic analysis of two SARS-CoV-2 strains isolated in Italy in January and February 2020: additional clues on multiple introductions and further circulation in Europe; Eur Surveill. 2020 Apr;25(13).doi: 10.2807/1560-7917.ES.2020.25.13.2000305

Wang D., Hu B., Hu C. Clinical characteristics of 138 hospitalized patients with 2019 novel coronavirus-infected pneumonia in Wuhan, China. JAMA 2020;323(11): 1061-1069

Williamson E, Walker AJ, Bhaskaran KJ et al. OpenSAFELY: factors associated with COVID-19-related hospital death in the linked electronic health records of 17 million adult NHS patients.medRxiv2020.05.06.20092999;doi: https://doi.org/10.1101/2020.05.06.20092999

World Health Organisation. Report of the WHO: China Joint Mission on Coronavirus Disease 2019 (COVID-19); 16–24 February 2020. https://www.who.int/docs/default-source/coronaviruse/who-china-joint-mission-on-covid-19-final-report.pdf (accessed 28 Aug 2020)

World Health Organization. Draft landscape of COVID-19 candidate vaccines. July 20, 2020 https://www.who.int/publications/m/item/draft-landscape-of-covid-19-candidate-vaccines (accessed 28 Aug 2020)

World Health Organisation. A Coordinated Global Research Roadmap: 2019 Novel Coronavirus. 2020 https://www.who.int/blueprint/priority-diseases/key-action/Coronavirus_Roadmap_V9.pdf (accessed 28 Aug 2020)

Xia et al. Effect of an Inactivated Vaccine Against SARS-CoV-2 on Safety and Immunogenicity Outcomes Interim Analysis of 2 Randomized Clinical Trials JAMA 2020; doi:10.1001/jama.2020.15543

Xia et al. Safety and immunogenicity of an inactivated SARS-CoV-2 vaccine, BBIBP-CorV: a randomised, double-blind, placebo-controlled, phase 1/2 trial Lancet 2020; DOI:https://doi.org/10.1016/S1473-3099(20)30831-8

Yang D, Leibowitz JL. The structure and functions of coronavirus genomic 3' and 5' ends. Virus Res. 2015;206:120–133

Zumla A, Hui DS, Azhar EI, Memish ZA, Maeurer M. Reducing mortality from 2019-nCoV: host-directed therapies should be an option. Lancet. 2020;395:e35–e36

Agrawal AS, Tao X, Algaissi a, et al Immunization with inactivated Middle East Respiratory Syndrome coronavirus vaccine leads to lung immunopathology on challenge with live virus. Hum Vaccin Immunother. 2016;12(9):2351-2356

Hondo-okubo Y, Barnard D, Ong CH, Peng BH; Tseng CT Severe acute respiratory syndrome associated vaccines formulated with delta inulin adjuvants provide enhances protection while ameliorating lung eosinophilic immunopathology. J. Virol. 2015;89(6):2995-3007.

*MHRA Guidance:

https://www.gov.uk/guidance/managing-clinical-trials-during-coronavirus-covid-19#remote-monitoring-for-trials

MHRA Blog:

https://mhrainspectorate.blog.gov.uk/2020/03/12/advice-for-management-of-clinical-trials-in-relation-to-coronavirus/

**HRA Guidance:

https://www.hra.nhs.uk/covid-19-research/covid-19-guidance-sponsors-sites-and-researchers/

Regulation 174 Information for UK healthcare professionals:

https://www.gov.uk/government/publications/regulatory-approval-of-covid-19-vaccine-astrazeneca/information-for-healthcare-professionals-on-covid-19-vaccine-astrazeneca
