# Supplementary material for: Immunogenicity and safety of an inactivated whole-virus COVID-19 vaccine (VLA2001) compared with the adenoviral vector vaccine ChAdOx1-S in adults in the UK (COV-COMPARE): interim analysis of a randomised, controlled, phase 3, immunobridging trial
Source: Lancet Infect Dis. 2022 Dec;22(12):1716–27. doi: 10.1016/S1473-3099(22)00502-3 (PMC9444237; doi:10.1016/S1473-3099(22)00502-3)
Supplement: Supplementary appendix 2 [file mmc2.pdf]

THE LANCET
Infectious Diseases

Supplementary appendix 2

This appendix formed part of the original submission and has been peer reviewed. We post it as supplied by the authors.

Supplement to: Lazarus R, Querton B, Corbic Ramljak I, et al. Immunogenicity and safety of an inactivated whole-virus COVID-19 vaccine (VLA2001) compared with the adenoviral vector vaccine ChAdOx1-S in adults in the UK (COV-COMPARE): interim analysis of a randomised, controlled, phase 3, immunobridging trial. *Lancet Infect Dis* 2022; published online Sept 5. https://doi.org/10.1016/S1473-3099(22)00502-3.

Supplementary Tables

Table S1 - Solicited Injection Site Reactions within 7 Days after the 1st Vaccination (Safety Population)

	Open-label VLA2001 group (age 18–29 years; N=1040)	Randomised VLA2001 group (age ≥30 years; N=1977)	ChAdOx1-S group (age ≥30 years; N=995)	Overall (N=3964)
**After 1st Vaccination**				
Participants with at least one Solicited Injection Site Reaction				
n (%)	696 (66·9)	1180 (59·7)	877 (88·1)	2753 (68·6)
95% CI [A]	(63·97, 69·78)	(57·49, 61·86)	(85·97, 90·08)	(67·16, 70·05)
p value [B]	··	··	··	<0·0001
Injection Site Tenderness				
n (%)	631 (60·7)	1025 (51·8)	831 (83·5)	2487 (62·0)
95% CI [A]	(57·63, 63·66)	(49·62, 54·07)	(81·06, 85·77)	(60·47, 63·49)
p value [B]	··	··	··	<0·0001
Injection Site Pain				
n (%)	398 (38·3)	597 (30·2)	622 (62·5)	1617 (40·3)
95% CI [A]	(35·30, 41·30)	(28·18, 32·27)	(59·42, 65·53)	(38·78, 41·84)
p value [B]	··	··	··	<0·0001
Injection Site Itching				
n (%)	41 (3·9)	60 (3·0)	64 (6·4)	165 (4·1)
95% CI [A]	(2·84, 5·31)	(2·32, 3·89)	(4·99, 8·14)	(3·52, 4·77)
p value [B]	··	··	··	<0·0001
Injection Site Induration				
n (%)	12 (1·2)	29 (1·5)	40 (4·0)	81 (2·0)
95% CI [A]	(0·60, 2·01)	(0·98, 2·10)	(2·89, 5·43)	(1·61, 2·50)
p value [B]	··	··	··	<0·0001
Injection Site Swelling				
n (%)	11 (1·1)	21 (1·1)	39 (3·9)	71 (1·8)
95% CI [A]	(0·53, 1·88)	(0·66, 1·62)	2·80, 5·32)	(1·38, 2·23)
p value [B]	··	··	··	<0·0001
Injection Site Redness				
n (%)	10 (1·0)	11 (0·6)	40 (4·0)	61 (1·5)
95% CI [A]	(0·46, 1·76)	(0·28, 0·99)	(2·89, 5·43)	(1·16, 1·95)
p value [B]	··	··	··	<0·0001

CI=confidence interval

Notes: Table presents solicited injection site reactions from participant diary

A Exact 95% Clopper-Pearson CI for proportion.

B p-value compares the VLA2001 (age ≥30) group with the ChAdOx1-S group.

Table S2 - Solicited Injection Site Reactions within 7 Days after the 2nd Vaccination (Safety Population)

	Open-label VLA2001 group (age 18–29 years; N=1026)	Randomised VLA2001 group (age ≥30 years; N=1949)	ChAdOx1-S group (age ≥30 years; N=989)	Overall (N=3964)
**After 2nd Vaccination**				
Participants with at least one Solicited Injection Site Reaction				
n (%)	667 (65·0)	1102 (56·5)	548 (55·4)	2317 (58·5)
95% CI [A]	(62·00, 67·93)	(54·31, 58·76)	(52·25, 58·54)	(56·90, 59·99)
p value [B]	··	··	··	0·5589
Injection Site Tenderness				
n (%)	608 (59·3)	982 (50·4)	490 (49·5)	2080 (52·5)
95% CI [A]	(56·18, 62·28)	(48·14, 52·63)	(46·38, 52·71)	(50·90, 54·04)
p value [B]	··	··	··	0·6670
Injection Site Pain				
n (%)	398 (38·8)	605 (31·0)	288 (29·1)	1291 (32·6)
95% CI [A]	(35·80, 41·85)	(28·99, 33·15)	(26·30, 32·06)	(31·11, 34·05)
p value [B]	··	··	··	0·2847
Injection Site Itching				
n (%)	30 (2·9)	61 (3·1)	30 (3·0)	121 (3·1)
95% CI [A]	(1·98, 4·15)	(2·40, 4·00)	(2·06, 4·30)	(2·54, 3·64)
p value [B]	··	··	··	0·8866
Injection Site Swelling				
n (%)	6 (0·6)	28 (1·4)	13 (1·3)	47 (1·2)
95% CI [A]	(0·21, 1·27)	(0·96, 2·07)	(0·70, 2·24)	(0·87, 1·57)
p value [B]	··	··	··	0·7896
Injection Site Induration				
n (%)	10 (1·0)	20 (1·0)	7 (0·7)	37 (0·9)
95% CI [A]	(0·47, 1·79)	(0·63, 1·58)	(0·29, 1·45)	(0·66, 1·28)
p value [B]	··	··	··	0·3928
Injection Site Redness				
n (%)	9 (0·9)	15 (0·8)	10 (1·0)	34 (0·9)
95% CI [A]	(0·40, 1·66)	(0·43, 1·27)	(0·49, 1·85)	(0·59, 1·20)
p value [B]	··	··	··	0·5007

CI=confidence interval

Notes: Table presents solicited injection site reactions from participant diary

A Exact 95% Clopper-Pearson CI for proportion.

B p-value compares the VLA2001 (age ≥30) group with the ChAdOx1-S group.

Table S3 - Solicited Injection Site Reactions within 7 Days after any Vaccination (Safety Population)

After any Vaccination				
	**Open-label VLA2001 group (age 18–29 years; N=1040)**	**Randomised VLA2001 group (age ≥30 years; N=1977)**	**ChAdOx1-S group (age ≥30 years; N=995)**	**Overall (N=4012)**
Participants with at least one Solicited Injection Site Reaction				
n (%)	841 (80·9)	1448 (73·2)	906 (91·1)	3195 (79·6)
95% CI [A]	(78·34, 83·21)	(71·23, 75·18)	(89·11, 92·76)	(78·36, 80·87)
p value [B]				<0·0001
Injection Site Tenderness				
n (%)	795 (76·4)	1320 (66·8)	871 (87·5)	2986 (74·4)
95% CI [A]	(73·74, 78·99)	(64·64, 68·84)	(85·32, 89·53)	(73·05, 75·77)
p value [B]	··	··	··	<0·0001
Injection Site Pain				
n (%)	550 (52·9)	900 (45·5)	671 (67·4)	2121 (52·9)
95% CI [A]	(49·80, 55·95)	(43·31, 47·75)	(64·43, 70·34)	(51·31, 54·42)
p value [B]	··	··	··	<0·0001
Injection Site Itching				
n (%)	64 (6·2)	107 (5·4)	89 (8·9)	260 (6·5)
95% CI [A]	(4·77, 7·79)	(4·46, 6·50)	(7·24, 10·89)	(5·74, 7·29)
p value [B]	··	··	··	0·0003
Injection Site Induration				
n (%)	20 (1·9)	45 (2·3)	44 (4·4)	109 (2·7)
95% CI [A]	(1·18, 2·95)	(1·66, 3·03)	(3·23, 5·89)	(2·24, 3·27)
p value [B]	··	··	··	0·0012
Injection Site Swelling				
n (%)	16 (1·5)	40 (2·0)	46 (4·6)	102 (2·5)
95% CI [A]	(0·88, 2·49)	(1·45, 2·75)	(3·40, 6·12)	(2·08, 3·08)
p value [B]	··	··	··	<0·0001
Injection Site Redness				
n (%)	17 (1·6)	25 (1·3)	48 (4·8)	90 (2·2)
95% CI [A]	(0·96, 2·60)	(0·82, 1·86)	(3·58, 6·35)	(1·81, 2·75)
p value [B]	··	··	··	<0·0001

CI=confidence interval

Notes: Table presents solicited injection site reactions from participant diary

A Exact 95% Clopper-Pearson CI for proportion

B p-value compares the VLA2001 (age ≥30) group with the ChAdOx1-S group

Table S4 –Solicited Systemic Reactions within 7 Days after the 1st Vaccination (Safety Population)

After 1st Vaccination				
	**Open-label VLA2001 group (age 18–29 years; N=1040)**	**Randomised VLA2001 group (age ≥30 years; N=1977)**	**ChAdOx1-S group (age ≥30 years; N=995)**	**Overall (N=3964)**
Participants with at least one Solicited Systemic Reaction				
n (%)	699 (67·2)	1187 (60·0)	876 (88·0)	2762 (68·8)
95% CI [A]	(64·26,70·06)	(57·84, 62·21)	(85·86, 89·99)	(67·38, 70·27)
p-value [B]	··	··	··	<0·0001
Fatigue				
n (%)	477 (45·9)	802 (40·6)	711 (71·5)	1990 (49·6)
95% CI [A]	(42·80, 48·95)	(38·39, 42·77)	(68·54, 74·25)	(48·04, 51·16)
p-value [B]	··	··	··	<0·0001
Headache				
n (%)	325 (31·3)	627 (31·7)	620 (62·3)	1572 (39·2)
95% CI [A]	(28·44, 34·17)	(29·67, 33·82)	(59·22, 65·33)	(37·67, 40·71)
p-value [B]	··	··	··	<0·0001
Muscle Pain				
n (%)	360 (34·6)	541 (27·4)	593 (59·6)	1494 (37·2)
95% CI [A]	(31·72, 37·60)	(25·41, 29·39)	(56·47, 62·66)	(35·74, 38·76)
p-value [B]	··	··	··	<0·0001
Nausea/Vomiting				
n (%)	115 (11·1)	168 (8·5)	189 (19·0)	472 (11·8)
95% CI [A]	(9·22, 13·12)	(7·31, 9·81)	(16·60, 21·57)	(10·78, 12·80)
p-value [B]	··	··	··	<0·0001
Fever/Body Temperature				
n (%)	4 (0·4)	17 (0·9)	143 (14·4)	164 (4·1)
95% CI [A]	(0·10, 0·98)	(0·50, 1·37)	(12·25, 16·71)	(3·50, 4·75)
p-value [B]	··	··	··	<0·0001

CI=confidence interval
Notes: Table presents solicited systemic reactions from participant diary.
A Exact 95% Clopper-Pearson CI for proportion.
B p-value compares the VLA2001 (age ≥30) group with the ChAdOx1-S group.

Table S5 –Solicited Systemic Reactions within 7 Days after the 2nd Vaccination (Safety Population)

After 2nd Vaccination				
	**Open-label VLA2001 group (age 18–29 years; N=1026)**	**Randomised VLA2001 group (age ≥30 years; N=1949)**	**ChAdOx1-S group (age ≥30 years; N=989)**	**Overall (N=3964)**
Participants with at least one Solicited Systemic Reaction				
n (%)	526 (51·3)	908 (46·6)	501 (50·7)	1935 (48·8)
95% CI [A]	(48·16, 54·37)	(44·35, 48·83)	(47·49, 53·82)	(47·25, 50·38)
p-value [B]	··	··	··	0·0369
Fatigue				
n (%)	355 (34·6)	597 (30·6)	340 (34·4)	1292 (32·6)
95% CI [A]	(31·69, 37·60)	(28·59, 32·73)	(31·42, 37·43)	(31·13, 34·08)
p-value [B]	··	··	··	0·0395
Muscle pain				
n (%)	257 (25·0)	410 (21·0)	224 (22·6)	891 (22·5)
95% CI [A]	(22·42, 27·82)	(19·25, 22·91)	(20·07, 25·39)	(21·19, 23·81)
p-value [B]	··	··	··	0·3153
Headache				
n (%)	235 (22·9)	392 (20·1)	255 (25·8)	882 (22·3)
95% CI [A]	(20·37, 25·60)	(18·35, 21·96)	(23·08, 28·63)	(20·96, 23·58)
p-value [B]	··	··	··	0·0005
Nausea/Vomiting				
n (%)	63 (6·1)	106 (5·4)	66 (6·7)	235 (5·9)
95% CI [A]	(4·75, 7·79)	(4·47, 6·54)	(5·20, 8·41)	(5·21, 6·71)
p-value [B]	··	··	··	0·1779
Fever/Body Temperature				
n (%)	7 (0·7)	12 (0·6)	15 (1·5)	34 (0·9)
95% CI [A]	(0·27, 1·40)	(0·32, 1·07)	(0·85, 2·49)	(0·59, 1·20)
p-value [B]	··	··	··	0·0156

CI=confidence interval
Notes: Table presents solicited systemic reactions from participant diary.
A Exact 95% Clopper-Pearson CI for proportion.
B p-value compares the VLA2001 (age ≥30) group with the ChAdOx1-S group.

Table S6 – Solicited Systemic Reactions within 7 Days after any Vaccination (Safety Population)

After any Vaccination				
	**Open-label VLA2001 group (age 18–29 years; N=1040)**	**Randomised VLA2001 group (age ≥30 years; N=1977)**	**ChAdOx1-S group (age ≥30 years; N=995)**	**Overall (N=4012)**
Participants with at least one Solicited Systemic Reaction				
n (%)	800 (76·9)	1387 (70·2)	906 (91·1)	3093 (77·1)
95% CI [A]	(74·24, 79·45)	(68·09, 72·17)	(89·11, 92·76)	(75·76, 78·39)
p-value [B]	··	··	··	<·0001
Fatigue				
n (%)	596 (57·3)	1012 (51·2)	767 (77·1)	2375 (59·2)
95% CI [A]	(54·24, 60·34)	(48·96, 53·41)	(74·35, 79·66)	(57·66, 60·72)
p-value [B]	··	··	··	<·0001
Headache				
n (%)	422 (40·6)	787 (39·8)	674 (67·7)	1883 (46·9)
95% CI [A]	(37·57, 43·63)	(37·64, 42·00)	(64·73, 70·64)	(45·38, 48·49)
p-value [B]	··	··	··	<·0001
Muscle pain				
n (%)	458 (44·0)	732 (37·0)	639 (64·2)	1829 (45·6)
95% CI [A]	(40·99, 47·12)	(34·89, 39·20)	(61·15, 67·20)	(44·04, 47·14)
p-value [B]	··	··	··	<·0001
Nausea/Vomiting				
n (%)	154 (14·8)	231 (11·7)	227 (22·8)	612 (15·3)
95% CI [A]	(12·70, 17·11)	(10·30, 13·18)	(20·24, 25·55)	(14·15, 16·40)
p-value [B]	··	··	··	<·0001
Fever/Body Temperature				
n (%)	11 (1·1)	29 (1·5)	154 (15·5)	194 (4·8)
95% CI [A]	(0·53, 1·88)	(0·98, 2·10)	(13·28, 17·88)	(4·19, 5·55)
p-value [B]	··	··	··	<·0001

CI=confidence interval
Notes: Table presents solicited systemic reactions from participant diary.
A Exact 95% Clopper-Pearson CI for proportion.
B p-value compares the VLA2001 (age ≥30) group with the ChAdOx1-S group.

Table S7 - Overall Summary of Unsolicited Adverse Events (Safety Population)

	Open-label VLA2001 group (age 18–29 years; N=1040)	Randomised VLA2001 group (age ≥30 years; N=1977)	ChAdOx1-S group (age ≥30 years; N=995)	Overall (N=4012)
Participants with any unsolicited AE until Day 43				
n (%)	300 (28·8)	566 (28·6)	349 (35·1)	1215 (30·3)
95% CI [A]	(26·11, 31·70)	(26·64, 30·68)	(32·11, 38·13)	(28·86, 31·37)
p-value [B]	··	··	··	0·0003
Participants with any unsolicited AE until interim analysis				
n (%)	423 (40·7)	793 (40·1)	443 (44·5)	1659 (41·4)
95% CI [A]	(37·67, 43·73)	(37·94, 42·31)	(41·40, 47·67)	(39·82, 42·89)
p-value [B]	··	··	··	0·0213
Participants with any serious unsolicited AE until Day 43				
n (%)	2 (0·2)	6 (0·3)	3 (0·3)	11 (0·3)
95% CI [A]	(0·02, 0·69)	(0·11, 0·66)	(0·06, 0·88)	(0·14, 0·49)
p-value [B]	··	··	··	0·9926
Participants with any serious unsolicited AE until interim analysis				
n (%)	7 (0·7)	14 (0·7)	10 (1·0)	31 (0·8)
95% CI A	(0·27, 1·38)	(0·39, 1·19)	(0·48, 1·84)	(0·53, 1·09)
p-value B	··	··	··	0·3034
Participants with any AESI until Day 43				
n (%)	2 (0·2)	1 (0·1)	2 (0·2)	5 (0·1)
95% CI [A]	(0·02, 0·69)	(0·00, 0·28)	(0·02, 0·72)	(0·04, 0·29)
p-value [B]	··	··	··	0·2230
Participants with any AESI until interim analysis				
n (%)	3 (0·3)	4 (0·2)	2 (0·2)	9 (0·2)
95% CI [A]	(0·06, 0·84)	(0·06, 0·52)	(0·02, 0·72)	(0·10, 0·43)
p-value [B]	··	··	··	0·9940
Participants with any treatment related unsolicited AE until Day 43				
n (%)	110 (10·6)	218 (11·0)	177 (17·8)	505 (12·6)
95% CI [A]	(8·77, 12·61)	(9·68, 12·49)	(15·46, 20·31)	(11·58, 13·65)
p-value [B]	··	··	··	<·0001
Participants with any medically attended unsolicited AE until Day 43				
n (%)	75 (7·2)	137 (6·9)	68 (6·8)	280 (7·0)
95% CI [A]	(5·71, 8·96)	(5·85, 8·14)	(5·35, 8·58)	(6·21, 7·81)
p-value [B]	··	··	··	0·9277
Participants with any treatment related unsolicited AE until interim analysis				
n (%)	113 (10·9)	227 (11·5)	178 (17·9)	518 (12·9)
95% CI [A]	(9·04, 12·92)	(10·11, 12·97)	(15·56, 20·42)	(11·89, 13·99)
p-value [B]				<·0001
Participants with any medically attended unsolicited AE until interim analysis				
n (%)	132 (12·7)	243 (12·3)	114 (11·5)	489 (12·2)
95% CI [A]	(10·73, 14·87)	(10·88, 13·82)	(9·54, 13·60)	(11·19, 13·24)
p-value [B]				0·5092

AE=adverse event; AESI=adverse event of special interest; CI=confidence interval
A Exact 95% Clopper-Pearson CI for proportion
B P-value tests difference in proportions between VLA2001 age ≥30 and ChAdOx1-S

Table S8 - Summary of Serious Unsolicited Adverse Events until interim analysis cut-off date (Safety Population)

System Organ Class<br>Preferred Term	Open-label VLA2001 group (age 18–29 years; N=1040)	Randomised VLA2001 group (age ≥30 years; N=1977)	ChAdOx1-S group (age ≥30 years; N=995)	Overall (N=4012)
Participants with at least one unsolicited serious AE	7 (0·7)	14 (0·7)	10 (1·0)	31 (0·8)
**Infections and infestations**	**4 (0·4)**	**3 (0·2)**	**2 (0·2)**	**9 (0·2)**
Abscess intestinal	1 (0·1)	0	0	1 (0·0)
Appendicitis perforated	0	1 (0·1)	0	1 (0·0)
Gastroenteritis	0	1 (0·1)	0	1 (0·0)
Gastroenteritis salmonella	0	0	1 (0·1)	1 (0·0)
Gastroenteritis viral	1 (0·1)	0	0	1 (0·0)
Meningitis viral	1 (0·1)	0	0	1 (0·0)
Pelvic inflammatory disease	0	0	1 (0·1)	1 (0·0)
Pyelonephritis	1 (0·1)	0	0	1 (0·0)
Sepsis	0	1 (0·1)	0	1 (0·0)
**Nervous system disorders**	**1 (0·1)**	**3 (0·2)**	**4 (0·4)**	**8 (0·2)**
Headache	0	0	2 (0·2)	2 (0·0)
Migraine	0	0	2 (0·2)	2 (0·0)
Cerebrospinal fluid leakage	0	1 (0·1)	0	1 (0·0)
Idiopathic intracranial hypertension	0	1 (0·1)	0	1 (0·0)
Nerve compression	0	1 (0·1)	0	1 (0·0)
Subarachnoid haemorrhage	0	0	1 (0·1)	1 (0·1)
Vestibular migraine	1 (0·1)	0	0	1 (0·0)
**Gastrointestinal disorders**	**0**	**2 (0·1)**	**1 (0·1)**	**3 (0·1)**
Abdominal pain	0	1 (0·1)	0	1 (0·0)
Inflammatory bowel disease	0	0	1 (0·1)	1 (0·0)
Mallory-Weiss syndrome	0	1 (0·1)	0	1 (0·0)
**Metabolism and nutritional disorders**	**1 (0·1)**	**1 (0·1)**	**0**	**2 (0·0)**
Hypercalcaemia	0	1 (0·1)	0	1 (0·0)
Type 1 diabetes mellitus	1 (0·1)	0	0	1 (0·0)
**General disorders and administration site conditions**	**0**	**0**	**1 (0·1)**	**1 (0·0)**
Non-cardiac chest pain	0	0	1 (0·1)	1 (0·0)
**Immune system disorders**	**0**	**1 (0·1)**	**0**	**1 (0·0)**
Food allergy	0	1 (0·1)	0	1 (0·0)
**Injury, poisoning and procedural complications**	**0**	**0**	**1 (0·1)**	**1 (0·0)**
Accidental overdose	0	0	1 (0·1)	1 (0·0)
Road traffic accident	0	1 (0·1)	0	1 (0·0)
**Renal and urinary disorder**	**0**	**0**	**1 (0·1)**	**1 (0·0)**
Renal colic	0	0	1 (0·1)	1 (0·0)

**Respiratory, thoracic and mediastinal disorders**	**0**	**1 (0·1)**	**0**	**1 (0·0)**
Dyspnoea	0	1 (0·1)	0	1 (0·0)
**Vascular disorders**	**0**	**1 (0·1)**	**0**	**1 (0·0)**
Embolism	0	1 (0·1)	0	1 (0·0)
**Musculoskeletal and connective tissue disorders**	**0**	**2 (0·2)**	**0**	**2 (0·2)**
Intervertebral disc protrusion	0	2 (0·2)	0	2 (0·2)
**Renal and urinary disorders**	**0**	**1 (0·1)**	**1 (0·1)**	**2 (0·2)**
Nephrolithiasis	0	1 (0·1)	0	1 (0·0)
Renal colic	0	0	1 (0·1)	1 (0·0)
**Blood and lymphatic system disorders**	**0**	**0**	**1 (0·1)**	**1 (0·0)**
Anaemia	0	0	1 (0·1)	1 (0·0)
**Reproductive system and breast disorders**	**1 (0·1)**	**0**	**0**	**1 (0·0)**
Ovarian cyst	1 (0·1)	0	0	1 (0·0)

AE=Adverse event; PT=preferred term; SOC=system organ class
Note: Participants with multiple unsolicited AEs within an SOC or SOC/PT combination are counted only once for that SOC or SOC/PT combination

Table S9 – Geometric Mean Titre of SARS-CoV-2 neutralising antibodies at Day 1 and Day 43 in Participants Aged 18-29 Years *vs* ≥30 Years who were MNA Baseline Seropositive

Visit<br>Statistic	Open-label VLA2001 group (age 18–29 years; N=67)	Open-label VLA2001 group (age ≥30 years; N=84)
**Visit 1 - Day 1**		
n	67	84
GMT (95% CI) [B]	418·3 (340·9, 513·2)	269·2 (226·4, 320·0)
Median	440·0	279·0
Min, Max	67·0, 6884·0	62·0, 6738·0
**Visit 4 - Day 43**		
n [A]	67	81
GMT (95% CI) [B]	2425·7 (2072·6, 2839·0)	1478·6 (1245·6, 1755·1)
Median	2400·0	1494·0
Min, Max	440·0, 12800·0	85·0, 12800·0

CI=confidence interval; GMT=geometric mean titre; Max=maximum; Min=minimum, n=number of participants with non-missing result; Q=quartile; SARS-CoV-2=Severe Acute Respiratory Syndrome Coronavirus-2.

A Eligible is defined as having a sample available for Day 43.

B CI calculated using a two-sided t-test applied to log10 transformed data.

Note: Values below the quantitation limit of the MNA (62) are replaced by 31. Values below the lower dilution limit are also replaced by 31. Values above the upper dilution limit are replaced by the upper dilution limit.

The Baseline Seropositive Analysis Set 18-29 years consists of all vaccinated participants 18-29 years of age who were seropositive at Day 1 and have at least one evaluable antibody titre measurement after vaccination.

Table S10 - IgG Antibody Titres against SARS-Cov-2 S-protein (ELISA) Over Time Points - (Immunogenicity IMM Population)

Visit<br>Statistic	Open-label VLA2001 group (age 18–29 years; N=210)	Randomised VLA2001 group (age ≥30 years; N=492)	ChAdOx1-S group (age ≥30 years; N=498)
**Day 1**			
n	210	489	496
GMT (95% CI) [A]	25·0<br>25·0, 25·0	25·2<br>25·03, 25·41	25·6<br>25·16, 25·96
Median	25·0	25·0	25·0
Min, Max	25·0, 25·0	25·0, 118·2	25·0, 763·7
Q1, Q3	25·0, 25·0	25·0, 25·0	25·0, 25·0
**Day 29**			
n	–	484	489
GMT (95% CI) [A]	–	44·3 (41·32, 47·46)	740·8 (680·90, 805·96)
Median	–	25·0	716·2
Min, Max		25·0, 15798.0	25·0, 15798·0
Q1, Q3	–	25·0, 69·5	367·5, 1628·1
**Day 43**			
n	210	492	493
GMT (95% CI) [A]	3121·5<br>(2699·3, 3609·7)	2361·7<br>(2171·08, 2569·11)	2126·4<br>(1992·42, 2269·45)
Median	3724·1	2898·7	2112·4
Min, Max	25·0, 15798·0	25·0, 15798·0	25·0, 15798·0
Q1, Q3	1776·5, 6762·0	1305·9, 5195·5	1216·6, 3846·6

CI=confidence interval; ELISA=enzyme-linked immunosorbent assay; GMT=geometric mean titre; Max=maximum; Min=minimum
A: CI calculated using log10 transformed

Table S11 - S-Protein Specific IgG Antibodies Seroconversion Rate (PP Population)

Visit	Open-label VLA2001 group (age 18–29 years; N=210)	Randomised VLA2001 group (age ≥30 years; N=489)	ChAdOx1-S group (age ≥30 years; N=498)
**Day 29**			
**Participants with seroconversion (≥4-fold increase)**			
n (%)	–	76 (15·5)	466 (93·6)
95% [A]	–	(12·6, 19·3)	(93·0, 97·0)
p-value for comparison VLA2001 Age ≥30 years vs. AZD1222 Age ≥30 years [B]	–	–	<0·0001
**Day 43**			
**Participants with seroconversion (≥4-fold increase)**			
n	208	456	450
n (%)	205 (98·6)	447 (98·0)	445 (98·9)
95% CI [A]	95·8, 99·7	96·3, 99·1	97·4, 99·6
p-value for comparison VLA2001 Age ≥30 years vs. ChAdOx1-S [B]	–	–	0·2914
95% CI for difference VLA2001 Age ≥30 years vs. ChAdOx1-S [A]	–	–	-0·025, 0·007

Note: Seroconversion was defined as ≥4-fold increase in SARS-CoV-2 S-protein binding IgG levels between Day 1 and post-vaccination sample collection time points.
A: Exact 95% Clopper-Person CI for proportion.
B: P value or 2-sided CI is for the difference in proportions (VLA2001 Age ≥30 years vs. ChAdOx1-S) of participants with seroconversion at each particular visit.

Table S12 - Cellular Immune Response (Reactogenicity Against Stimulation Panel) – Reactive Percentages on Day 1 and Day 43 (PBMC Population)

Visit<br>Panel	Randomised VLA2001 group (age ≥30 years; N=101)	ChAdOx1-S group (age ≥30 years; N=103)	Overall (N=990)
**Panel 1: spike protein N terminus**			
**Day 1**			
n	77	79	156
Reactive (%)	0	0	0
**Day 43**			
n	74	74	148
Reactive (%)	38 (51·4)	58 (78·4)	96 (64·9)
**Panel 2: spike protein C terminus**			
**Day 1**			
n	77	79	156
Reactive (%)	1 (1·3)	1 (1·3)	2 (1·3)
**Day 43**			
n	74	74	148
Reactive (%)	32 (43·2)	43 (58·1)	75 (50·7)
**Panel 3: nucleocapsid protein**			
**Day 1**			
n	77	79	156
Reactive (%)	0	0	0
**Day 43**			
n	74	74	148
Reactive (%)	34 (45·9)	1 (1·4)	35 (23·6)
**Panel 4: membrane protein**			
**Day 1**			
n	77	79	156
Reactive (%)	1 (1·3)	0	1 (0·6)
**Day 43**			
n	74	74	148
Reactive (%)	15 (20·3)	0	15 (10·1)
**Panel 14: spike protein, full sequence**			
**Day 1**			
n	77	79	156
Reactive (%)	2 (2·6)	3 (3·8)	5 (3·2)
**Day 43**			
N	74	74	148
Reactive (%)	55 (74·3)	64 (86·5)	119 (80·4)

n=the number of samples available for analysis; PBMC=peripheral blood mononuclear cells; SFU=spot forming units. Note: The sample was considered reactive against an individual stimulation panel if normalized SFU≥6 (SFU in unstimulated control wells (medium only control) subtracted). The denominators for the percentages are the number of available samples at each visit within each parameter. Data are based on spot forming units per 2.5×10^5 PBMC.

Table S13 – Exploratory endpoint: Hazard Ratio Analysis for evaluable COVID-19 cases

	**Randomised VLA2001 group (age ≥30 years) n (%)**	**Randomised VLA2001 group (age ≥30 years) n (%)**	**ChAdOx1-S group (age ≥30 years) n (%)**	**All participants n (%)**
**Hazard Ration Analysis for evaluable COVID-19 cases (14 days after Second Vaccination)**				
yes	69 (7·3)	88 (4·9)	42 (4·5)	199 (5·4)
no	882 (92·7)	1706 (95·1)	899 (95·5)	3487 (94·6)
Total	951	1794	941	3686

Maximum Likelihood Parameter Estimates	**Pr > Chi-Square**	**Hazard Ratio [95% Wald CI]**
**Group: 'VLA2001 ≥30 yrs' vs. ChAdOx1-S**	**0·9276**	**0·98 [0·68, 1·42]**

Infections after non-study COVID-19 vaccination are not considered. Only data up to data cut 14-OCT-2021 is considered. Participants with infection or non-study COVID-19 vaccination earlier than 2 weeks after 2nd vaccination are not analyzed. One participant from group VLA2001 ≥30 years with reported infection could not be included in this analysis, since the date of infection was not reported. Percentages are based on the number of non-missing observations (total).

Figure S1 – Cellular Immune response: reactogenicity against stimulation panel

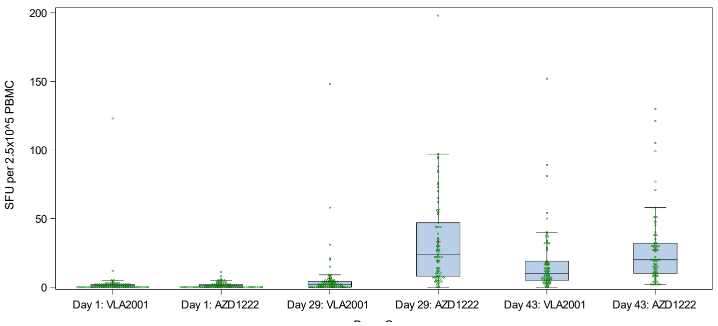

Panel A - Plot of Interferon Gamma Spot Forming Units per 2·5 × 10^5 PBMC by Dose Groups and Assessment Days for Panel 14 Spike Protein, Full Sequence.

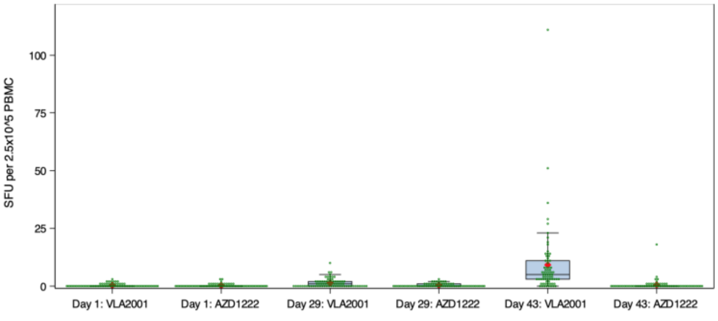

Panel B - Plot of Interferon Gamma Spot Forming Units per 2·5 × 10^5 PBMC by Dose Groups and Assessment Days for Panel 3 Nucleocapsid Protein, .

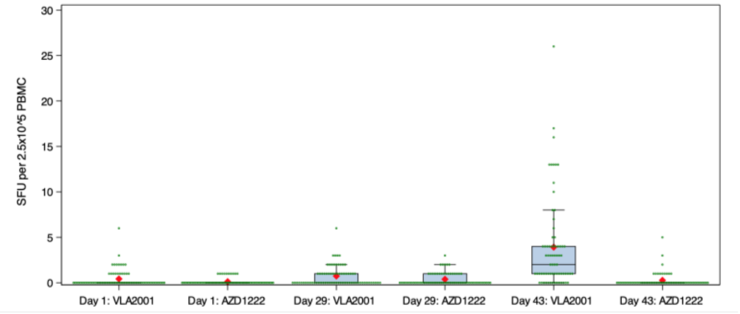

Panel C - Plot of Interferon Gamma Spot Forming Units per 2·5 × 10^5 PBMC by Dose Groups and Assessment Days for Panel 4 Membrane Protein.

Note Boxplots show median, lower quartile and upper quartile; the horizontal line within each bar is the median and red diamond represents mean value for each group. Values have been normalized to the respective unstimulated control (SFU in medium only control was substracted). Green scatter dots are the actual distribution of SFU per 2·5×10^5 PBMC within each group.
